# A regulatory compliant short-term oral toxicity study of soluble [60]fullerenes in rats

**DOI:** 10.17179/excli2024-7084

**Published:** 2024-05-15

**Authors:** Christopher Burres, Robert Wong, Fabio Pedreira, Maria Da Silva Pimenta, Fathi Moussa

**Affiliations:** 1 SES Research Inc., Houston, TX/USA; 2 Institut de Chimie Physique, CNRS - UMR 8000, Université Paris-Saclay; Gif-sur-Yvette 13 avenue des Sciences, 91190, France

**Keywords:** fullerene, C60, acute oral toxicity, regulation

## Abstract

Thirty-eight years after its discovery, the safety of [60]fullerene (C60), the most abundant fullerene with many potential applications, particularly in oxidative stress-related medicine, remains controversial. This is mainly due to the alleged dangers of C60 nanomaterial, which are regularly supported by some publications. While several academic studies have confirmed the safety of C60 in various experimental models, it is well known that C60 aggregates can carry toxic elements. Meanwhile, countless websites offer C60-oily solutions to consumers, without any regulatory consideration. Therefore, an officially certified toxicity study is urgently needed to avoid any public health problems. In this context, we report on the first certified short-term oral toxicity study of soluble C60, designed according to the guidelines of the Organization for Economic Cooperation and Development, with a deviation in the duration (2 weeks instead of 4 weeks) accepted by the U.S. Food and Drug Administration. The results of this study, conducted in an independent accredited European Laboratory, clearly show that C60 in soluble form (0.8 mg/ml of extra virgin olive oil), administered at the highest possible dose of 3.8 mg/kg body weight/day, did not cause any adverse effects in rats after 14 days of daily oral administration. This report should settle the debate on the acute oral toxicity of C60 and pave the way for further preclinical studies. The study is accompanied by a comprehensive report that includes documentation of the raw data.

## Introduction

**Figure 1:**
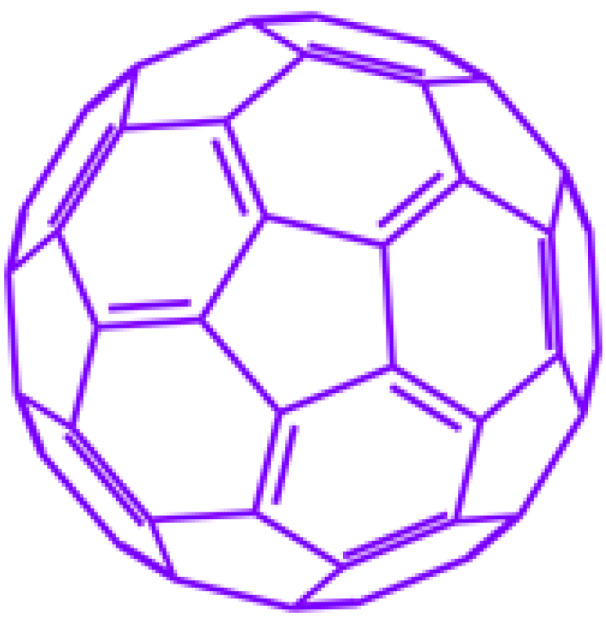
[60]Fullerene or C60

Since its discovery in 1985, [60]Fullerene (or C60) (Figure 1(Fig. 1)), the most abundant fullerene (Kroto et al., 1985[28]), has offered great potential for numerous applications in several fields including the biomedical field (Jensen et al., 1996[21]). This potential is based on the unique physicochemical properties of this pure carbon-based molecule, including its spherical shape and size (Haddon, 1993[17]; Friedman et al., 1993[10]; Cagle et al., 1999[5]), its ability to sensitize singlet oxygen formation under light exposure (Arbogast et al., 1991[2]; Tokuyama et al., 1993[49]), and its unmatched affinity for free radicals, thanks to its 30 double bonds (Krusic et al., 1991[29]).

Despite its insolubility in water, the rapid progress in C60 chemistry (Diederich and Thilgen, 1996[7]) allowed for dozens of well-characterized water-soluble C60-derivatives intended for many biomedical applications (Jensen et al., 1996[21]). The earliest review on this topic published 26 years ago described all potential biomedical applications of C60 thus predicting a large panel of fullerene-based drug candidates (Friedman et al.,1993[10]; Jensen et al., 1996[21]; Dugan et al., 1997[8]). Since then, most publications and general reviews published to date, including the most recent (Geng et al., 2017[13]; Kerna et al., 2020[24]; Nozdrenko et al., 2021[38]; Stetska et al., 2021[48]; Ye et al., 2021[53]; Chen et al., 2021[6]; Martínez-Herrera et al., 2022[33]; Grebinyk et al., 2023[15]; Sinegubova et al., 2023[46]; Li et al., 2023[31]; Beyaz et al., 2023[4]; Heflich, 2023[18]), have only repeated these predictions.

To date, countless publications have reported on the beneficial effects of C60 and its derivatives on health. To cite only the earliest publications, these beneficial effects range from anti-allergic and anti-inflammatory effects (Ryan et al., 2007[42]; Shershakova et al., 2016[44]) to life extension (Quick et al., 2008[40]; Gao et al., 2011[12]; Baati et al., 2012[3]; Galvan et al., 2017[11]), theranostic (Kawasaki et al., 2022[22]), protection of the nervous system (Dugan et al., 1997[8]; Stetska et al., 2021[48]; Martínez-Herrera et al., 2022[33]) and liver (Gharbi et al., 2005[14]), protection against ionizing radiation (Andrievsky et al., 2009[1]), and even prevention of acne vulgaris (Inui et al., 2011[20]) and hair growth potentiation (Zhou et al., 2009[54]). However, none of these promising applications have materialized and there are no fullerene-based medicines on the legal market (Kepley, 2012[23]). The main obstacle is related to the alleged danger of C60 nanoparticles raised by some studies since the advent of the US national nanotechnology initiative (US NNI, 2003[52]; Kipen and Laskin, 2005[26]).

Since 1995, pioneering works have shown that pure micro- and nano-crystals of C60 can cross the cell membranes (Moussa et al., 1995[36]) and when administered to mice at doses up to 5 g/kg body weight (bw) had no acute or subacute toxic effects in this rodent species (Moussa et al., 1996[37]). The safety of pure C60 has since been confirmed by numerous independent international teams in various experimental models encompassing different clades, as previously detailed in a general review (Kolosnjaj et al., 2007[27]). Nonetheless, some papers continue to propagate doubt by claiming that this fullerene is harmful (Đurašević et al., 2020[9]; Masyutin et al., 2020[34]; Grohn et al., 2021[16]; Malhotra et al., 2021[32]; Ren et al., 2022[41]), thus fueling the distrust of public health institutions (SCCS, 2023[43]) as well as the disinterest of the pharmaceutical industry. Yet, it has been clearly established that toxic effects observed for some C60 preparations are exclusively due to impurities or light exposure (Henry et al., 2007[19]; Kolosnjaj et al., 2007[27]; Spohn et al., 2009[47]). The main problem is that under certain conditions, linked to the presence of impurities or to light exposure, certain C60-based preparations and some C60-derivatives can be highly toxic (Kolosnjaj et al., 2007[27]). In fact, any time C60 is tested for toxicity without checking its purity and the purity of the solvent or adjuvant used, while avoiding light exposure, deleterious effects are usually observed (Henry et al., 2007[19]; Kolosnjaj et al., 2007[27]; Spohn et al., 2009[47]; Grohn et al., 2021[16]). But, as expected, reporting toxic effects for a new material like C60, often erroneously referred to as C60 nanoparticles, has much more impact than the absence of toxicity, notably because of certain fears related to the development of nanotechnology (Kipen and Laskin, 2005[26]; Keykhosravi et al., 2019[25]). In a recent report, the experts of the Scientific Committee on Consumer Safety (SCCS, 2023[43]) of the European Commission wrote the following: “*Having assessed the information provided by the notifiers, and the information available from the published literature, the SCCS cannot conclude on the safety of fullerenes and (hydrated) hydroxylated forms of fullerenes due to a number of uncertainties and data gaps in regard to physicochemical, toxicokinetic and toxicological aspects*”.

Meanwhile, since the publication of a paper in 2012 reporting on the effects of repeated administration of C60 on the lifespan of rats (Baati et al., 2012[3]), myriad startups have started selling this product online as a dietary supplement without any toxicity test (Keykhosravi et al., 2019[25]; Kerna et al., 2020[24]). Alas, C60 consumption without regulation could cause a major public health issue. Obviously, this would be detrimental to the health of consumers, but such an issue could also compromise a product that seems so promising for health. Hence, in order to avoid any public health issue and in order to provide a clear answer to the suspicions of toxicity, preclinical studies are urgently needed. In any case, in order to give C60 a chance to fulfill the hopes and expectations it raises in the biomedical field, it is mandatory to first carry out certified toxicity studies (Keykhosravi et al., 2019[25]; Kerna et al., 2020[24]).

Here we report on the first certified 14-day repeated-dose oral (by gavage) toxicity study in rats of C60 dissolved in extra-virgin olive oil (EVO). The aim of this study was to obtain first-hand information on the potential toxicity of C60 in soluble form.

## Materials and Methods

All experimental protocols were approved by the Hungarian Good Laboratory Practice Regulation: 42/2014 (VIII. 19.) EMMI decree of the Minister of Human Capacities which corresponds to the OECD GLP, ENV/MC/CHEM(98)17) [OECD Principles of GLP as revised in 1997, published in ENV/MC/CHEM (98)17); OECD, Paris, 1998] (supplementary information, Appendix 16). The study was performed according to GLP at the Sponsor's request because of authority purposes. The Principles of Good Laboratory Practice as specified by Hungarian and international legislations were followed (except for formulation analysis).

The study followed the procedures indicated by the following internationally accepted guidelines and recommendations: - OECD Guidelines for Testing of Chemicals[39], Section 4 Health Effects; No. 407, “*Repeated Dose 28-Day Oral Toxicity Study in Rodents*” (adopted 03 October 2008), and - US FDA Toxicological Principles for the Safety Assessment of Food Ingredients[51], Redbook 2000, IV.C. 3.a. *Short-Term Toxicity Studies with Rodents *(2007). All methods are reported in accordance with ARRIVE guidelines.

All procedures described in the study plan are based on Standard Operating Procedures (SOPs) detailed in the SOP manuals of the operational departments of Toxi-Coop Zrt. QA has reviewed the study plan, the various phases of the study, and certain recurring activities, and the report has been reviewed in accordance with internal SOPs.

Institutional Animal Care and Use Committee (IACUC) of Toxi-Coop Zrt. Permitted the conduct of the study by signature on the Study Plan. (SOP: ALT 023 - Instructions for animal protection). The study was conducted in accordance with the National Research Council Guide for the Care and Use of Laboratory Animals and in compliance with the principles of Hungarian Act 2011 CLVIII (amendment of Hungarian Act 1998 XXVIII) and Government Decree 40/2013 on the Protection of Animals.

The study documents and samples are archived according to the OECD GLP and to the Toxi-Coop Zrt.'s SOP-s in the archives of Toxi-Coop Zrt. Berlini utca 47-49. H-1045 Budapest, Hungary: - the Study Plan for 15 years, - one original Final Report for 15 years, - one sample of the test item for 5 years, - all raw data for 15 years, - biological samples for 5 or 12 years, - organs and tissues preserved in 4 % buffered formaldehyde solution for 5 years, - blocks and slides of organs and tissues for 12 years, - correspondence for 15 years. 

Raw data, and legal attestations related to the study are included in the additional information (supplementary information, Appendices 9 - 16). In fact, the supplementary information only consists of the original report as provided by the accredited European Laboratory who performed the entire study.

## Experimental Design

### Test system

Extra-virgin olive oil (EVO) and C60 (purity 99.98 %) were obtained from SES Research Inc. (USA) (SI, Appendix 1, Certificates of analysis, in the copy of the study plan, Appendix 9). We have previously detailed the physicochemical properties of the C60 used (purity: 99.98 %) by using various analytical methods, both in the solid state and in solution (Keykhosravi et al., 2019[25]).

EVO was chosen as the solvent because of its nutritional properties and especially the solubility of C60, which is extremely hydrophobic, in this oil (Baati et al., 2012[3]). In addition, the Olive oil-C60 product is offered online for human consumption in several countries, including the U.S. (Keykhosravi et al., 2019[25]; Kerna et al., 2020[24]).

The solution of EVO-C60 was prepared as previously described (Baati et al., 2012[3]) with minor modification. Briefly, 0.09 g of C60 was dissolved in 100 ml of olive oil with stirring for 2 weeks in the dark at room temperature. The resulting mixture was then centrifuged at 5,000 g for 60 min and the supernatant was filtered through a Millipore 0.25 µm pore size filter. The resulting solution is a transparent reddish-brown liquid with a faint oily odor containing 800 - 870 mg C60 per kg EVO as determined by liquid chromatography (supplementary information, Appendix 9, Certificate of Analysis).

### Test animals

We chose the rat for this study because it is the preferred rodent species for this type of study according to OECD guidelines (2008[39]). In addition, basic data on the absorption, pharmacokinetics, and biodistribution of the test substance in the rat are already available in the scientific literature (Kubota et al., 2011[30]; Baati et al., 2012[3]) whereas that data remain unknown in other animals including mice (Grohn et al., 2021[16]; Shytikov et al., 2021[45]).

*Number and groups*. Twenty specific pathogen-free Wistar rats (Han:WIST rat of Wistar origin, from Toxi-Coop Zrt, Budapest, Hungary), including ten males (42 - 45 days old, weighing 170 - 184 g) and ten nulliparous, non-pregnant females (41 - 43 days old, weighing 124 - 140 g) were used in the study, in accordance with OECD guidelines (2008[39]).

Animals were selected for this study on the basis of adequate body weight, a body weight within ± 20 % of the mean within a sex and free from clinical signs of disease or injury (OECD, 2008[39]). Selected rats were randomly divided into two groups (1 test item treated group + 1 control group) of 10 individuals (5 males and 5 females) per group according to body weight stratification such that there was no statistically significant difference between group body weight means within a sex. Grouping was controlled by the SPSS/PC+ computer program on the basis of actual body weight, checking for homogeneity and differences between groups.

Animals were identified by unique numbers. Individual identification was done with a marker pen on the tail. Identification numbers were assigned to each animal based on Toxi-Coop Zrt.'s master file, and numbers were re-marked as necessary to ensure correct identification.

*Husbandry*. Rats were housed in individual polypropylene/polycarbonate type III cages and maintained in an air-conditioned room where temperature (22 ± 3 °C) and relative humidity (30 to 70 %) were controlled and recorded daily during the study, on a 12-hour artificial light/dark cycle (6 am to 6 pm, except on days of ophthalmic examinations). Cages were labeled with identification cards containing the study number, control or test name, group number, test serial number, sex, cage number and individual animal numbers, treatment start date, and necropsy date. Cages were arranged to minimize potential effects of cage placement.

Rats were given ad libitum access to tap water and food (ssniff® SM R/M-Z+H complete diet for rats and mice, from ssniff Spezialdiäten GmbH, D-59494 Soest, Germany), except for overnight food deprivation prior to blood collection and they were acclimated for 5 days prior to treatment. The diets were considered to be free of contaminants that could reasonably be expected to interfere with the purpose or integrity of the study (supplementary information, Appendix 11). The Government Office of the Capital City of Budapest, Department of Public Health and Medical Service (Budapest, H-1138 Hungary) controls the water quality every six months. The results of the quality control are recorded in the archives of Toxi-Coop Zrt. (supplementary information, Appendix 12).

### Test details

*Rationale for route of administration and dose levels****. ***The oral route of administration is the intended route of human exposure. The test substance is already offered online for human consumption as a dietary supplement (Keykhosravi et al., 2019[25]; Kerna et al., 2020[24]).

The OECD (2008[39]) and US FDA (2007[51]) guidelines recommend using a minimum of three dose levels of the test substance and a concurrent control group in toxicity studies. These test guidelines suggest that the highest dose level should be carefully selected to induce toxic responses in test animals without leading to fatalities or severe suffering. However, the OECD guidelines (2008[39]) specify that: “Generally, at least three test groups and a control group should be used, but if from assessment of other data, no effects would be expected at a dose of 1000 mg/kg bw/day, a limit test may be performed.” 

In fact, it has been demonstrated for a number of years that doses in excess of 1000 mg/kg bw do not result in toxic effects in rodents (Moussa et al., 1996[37]; Gharbi et al., 2005[14]). These results have since been confirmed by other independent laboratories (Kolosnjaj et al., 2007[27]), notably in Japan (Mori et al., 2006[35]) and Switzerland (Spohn et al., 2009[47]). Therefore, in this case, it was logical and obvious to choose a single dose limit test (OECD, 2008[39]). 

The objective of this study was to obtain preliminary information on the toxic potential of C60 dissolved in EVO in groups of male and female rats, likely to result from repeated exposure. In this case, since it was not possible to select the highest dose level to induce toxic responses in test animals without causing death or severe suffering, as suggested by OECD guidelines (2008[39]), we selected the highest dose level that could be administered to rodents for oily solutions. The highest dose level of EVO-C60 that can be administered to rodents is 3.8 mg/kg bw/day. This dose level is based on the solubility of C60 in olive oil (Baati et al., 2012[3]) and the maximum volume to be administered to rodents according to the official recommendations (US FDA, 2007[51]). This dose level was previously used in a pharmacokinetic study and showed effective absorption and good biodistribution (Baati et al., 2012[3]).

*Duration*. While OECD guidelines (2008[39]) suggest 28 days for short-term acute toxicity studies, FDA guidelines (2007[51]) suggest 2 to 4 weeks. After discussion and agreement with the FDA staff responsible for this guidance, it was deemed sufficient to conduct this preliminary repeated oral dose study for only 2 weeks. In fact, this study was conducted as part of an FDA submission process.

## Experimental Procedure

After 5 days of acclimatization, the test substance (EVO-C60) and the vehicle (EVO) were administered orally by gavage at approximately the same time each morning within 2-3 hours from day 0 to day 13 (14 days). Animals were not treated on the day of gross pathology. The first treatment day was study day 0, and necropsy was performed on day 14. 

The control group (group 1) received EVO only at a dose of 5 ml/kg bw/day, and the experimental group (group 2) received 5 ml/kg bw/day of EVO-C60, equivalent to 4 (3.8) mg C60/kg bw/day. Actual treatment volume was calculated based on final body weight.

### Mortality and clinical observations

Animals were examined twice daily, at the beginning and end of each working day, for signs of morbidity and mortality. At approximately the same time after treatment, clinical observations were made once daily in the cage. On the day before the first treatment and then once a week, on days when the animals were weighed and food consumption was measured, detailed observations were made during handling.

The signs that are examined and recorded focus on the condition of the skin and coat, eyes and mucous membranes; the presence of secretions and excretions; autonomic activity (lacrimation, piloerection, pupil condition, unusual respiratory rhythm, etc.). Changes in gait, posture, and response to handling, as well as the presence of abnormal movements, stereotyped activities (e.g., excessive grooming, repetitive turning), or bizarre behaviors (e.g., self-mutilation, walking upside down) are also considered.

### Body weight and body weight gain

Animals were weighed twice during the acclimation period. Body weight was measured to the nearest 1 g on day 0 (before the start of the study) and twice weekly (i.e., on days 0, 3, 7, 10, and 13). Animals were also weighed immediately before sacrifice to calculate organ-to-body weight ratios. Individual changes in body weight were calculated according to the days of measurement and for the study as a whole.

### Food consumption measurement and feed efficiency

Food consumption was determined by measuring food consumed and food not consumed to an accuracy of 1 g once a week to coincide with body weight measurements (days 0, 7, and 13). Food consumption was evaluated and reported by weekly interval for each group. Feed efficiency was calculated based on weekly body weight gain and food consumption. All animals were fasted overnight (approximately 16 hours) before blood sampling.

### Clinical pathology examinations

Clinical pathology, including hematology, coagulation, and clinical chemistry, was performed one day after the last treatment (day 14). After isoflurane CP® anesthesia, three blood samples were collected from the retro-orbital venous plexus of each animal for hematology, clotting times, and clinical chemistry.

*Hematology*. ^Blood samples were collected in tubes containing K3EDTA (MiniCollect® 0.5 ml, Greiner Bio-One International AG, Kremsmünster, Austria) to the indicated final volume. ^Analysis was performed immediately after sampling. The parameters studied and the methods used, as measured by a Siemens ADVIA120 hematology system, are summarized in Table 1(Tab. 1) (Reference in Table 1: OECD, 2008[39]).

*Blood coagulation. *For clotting times (APTT: activated partial thromboplastin time and PT: prothrombin time), blood samples were collected in tubes containing 9NC Coagulation 3.8 % (MiniCollect® 1 mL; Greiner Bio-One International AG, Kremsmünster, Austria). After centrifugation at 2500 rpm for 15 minutes (within 20-30 minutes after collection), the plasma supernatants were measured (optical method) immediately by an AMAX Destiny Plus Coagulation Analyzer (Trinity Biotech PLC).

*Clinical chemistry*. Clinical chemistry samples (a minimum of 1.0 mL) were collected in non-anticoagulant tubes (Vacuette 2.5 mL Z Serum Sep C/A, Greiner Bio-One International AG, Kremsmünster, Austria). After centrifugation (4500 rpm for 15 minutes), serum samples were stored and analyzed at 2-8 °C.

Table 2(Tab. 2) (Reference in Table 2: OECD, 2008[39]) summarizes the parameters measured and methods, using a Cobas C311 (*Roche* Diagnostics International Ltd.).

### Pathology

*Necropsy*. Gross pathology was performed on each experimental animal one day after the last treatment on day 14 of the study. Animals were anesthetized with Isoflurane CP® and exsanguinated through the abdominal aorta after confirmation of anesthesia.

After examining the external appearance (body and orifices) and opening the skull and the thoracic and abdominal cavities, the appearance of the tissues and organs was observed and the observations were recorded in detail (location, color, shape and size).

The organs/tissues listed In Table 3(Tab. 3) (Reference in Table 3: OECD, 2008[39]) were removed and preserved in 4 % formaldehyde solution, except for testes and epididymides, which were preserved in modified Davidson's solution and then stored in 4 % formaldehyde solution for histopathological examination. 

Thyroid and parathyroid glands were preserved with the larynx, but the larynx was not processed for histology.

After excision, the organs and tissues were cleaned of any adherent tissue, weighed, and stored as described above.

*Organ weight. *The following organs were weighed and recorded with an accuracy of 0.01 g for liver, kidneys, testes, epididymides, prostate, seminal vesicles with coagulation glands as a whole, uterus and fallopian tubes, thymus, spleen, brain and heart, and with an accuracy of 0.001 g for adrenals, ovaries. Paired organs were weighed together.

*Histopathology*. Preserved organs and tissues from each animal were subjected to complete histologic examination.

After paraffin embedding, the fixed tissues were sectioned using a 2-4 µm microtome. After mounting on glass slides, the sections were stained with hematoxylin and eosin and examined by light microscopy.

## Statistics and Data Evaluation

SPSS PC+ software was used for statistical analysis of all data collected.

Bartlett's test was used to test for heterogeneity of variance between groups. If positive, Duncan's multiple range test was used to assess the significance of the differences between groups. If no significant heterogeneity was found, a one-way analysis of variance was performed. 

The Kolmogorov-Smirnov test was used to check the normal distribution of the data, in case of significant heterogeneity. The Kruskal-Wallis method was used in case of non-normal distribution. Finally, the Mann-Whitney U test was used to compare groups, for any positive results.

The frequencies of clinical signs, pathological and histopathological findings by sex and dose were calculated.

## Results

### Mortality, clinical signs and growth

No mortality was observed in any of the tested groups during the entire observation period. Likewise, throughout the observation period, no clinical signs related to the test item were observed and the behavior and physical state of all animals remained normal (supplementary information, Appendices 1.1, 1.2, 1.3, and 1.4).

**Figure 2:**
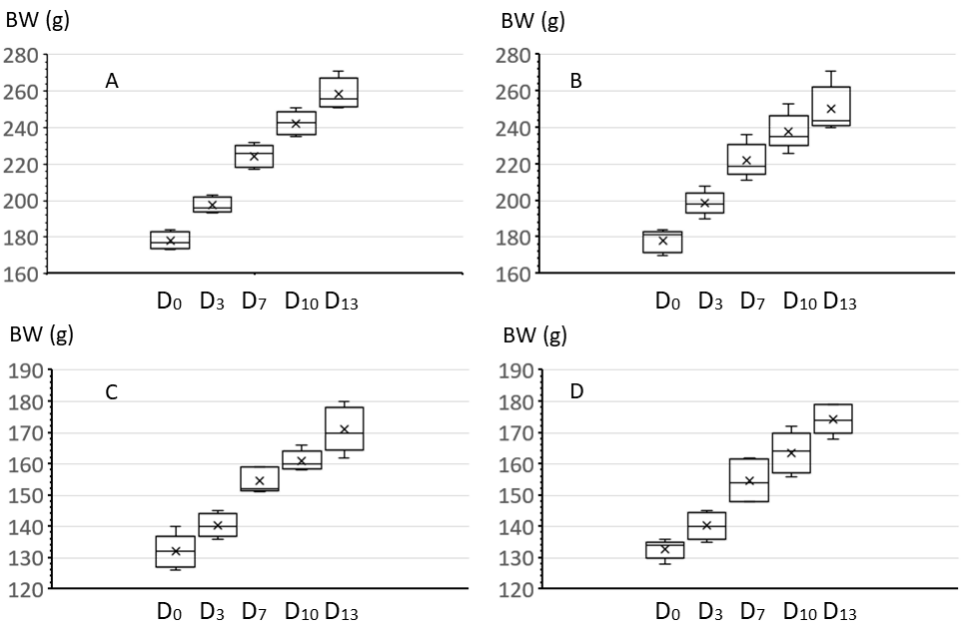
Body weight (BW) development of rats from day 0 (D_0_) to 13 (D_13_), after oral administration of extra virgin olive oil (control) or EVO infused with C60 (C60). (A): Male control; (B) male C60; (C) female control; and (D) female C60. Each box represents the 5 animals of the considered group.

The bw development was undisturbed In male and female animals of OO-C60 treated group as compared to the control group during the entire study (Figure 2(Fig. 2), and supplementary information, Appendices 1). Also, the main food consumption and the feed efficiency were comparable in female and male animals in all groups during the entire study (supplementary information, Appendices 2 and 3).

### Hematology, blood coagulation, and clinical chemistry data

Test items related adverse effects were not identified in the examined hematological or blood coagulation parameters in the male or female animals in the EVO-C60 treated group as compared to the control group (supplementary information, Appendices 4.1 and 4.2).

Specific pathologic changes were not detected in the examined clinical chemistry parameters (supplementary information, Appendices 5.1 and 5.2). Statistically significant mean concentration of glucose (GLU) was detected in male animals in the EVO-C60 treated group. However, these statistically significant differences with respect to the control in glucose (supplementary information, Appendices 5.1 and 5.2) were probably not related to the test item. The glucose levels of male animals remained well within the historical control range (supplementary information, Appendix 14). Therefore, these minor changes were judged to be indicative of biological variation and not related to any of the test items.

### Necropsy - histopathology - organ weight

At the necropsy, EVO or test item treatments did not induce specific macroscopic alterations in the tissues or organs of female or male animals. There were not statistically or biologically significant differences with respect to the control in the mean weight of the examined organs at the end of the study (supplementary information, Appendices 6 and 7). However, some species-specific changes (scar on the skin, pyelectasia, hydrometra and cyst in the uterus) and individual lesions (thymic hemorrhage, reddish-brown spot on the lung lobe) were detected in female and male animals as summarized in Table 4(Tab. 4).

Alveolar emphysema and acute hemorrhages in the thymus and lungs occurred sporadically in control and treated animals. These findings could be related with the hypoxia, dyspnea and circulatory disturbance, developed during the exsanguinations as frequently observed in experimental rats. Pyelectasia is a common observation in experimental rats of this strain occurring also in not treated animals. In female rats, the dilation of the uterine horns is a neurohormonal phenomenon linked to the sexual function (proestrus phase) of the internal genital organs. Also, in experimental rats, hydrometra is also a frequent observation linked to the female sexual cycle. In the absence of inflammatory, necrotic or other pathological lesions, these results are considered toxicologically irrelevant.

Finally, the observation of the following organs and systems: gastrointestinal tract, liver, pancreas, cardiovascular system, urinary system, lymphoid system, hematopoietic system, skeleton, muscular system, male and female reproductive system, central or peripheral nervous system, eyes, lacrimal glands, and integumentary system did not reveal any morphological signs of acute or subacute lesion (degeneration, proliferation, inflammation, necrosis, etc.). Also, the structure and the cell morphology of the endocrine glands were identical in the control and treated animals (supplementary information, Appendices 7.1 and 7.2).

## Discussion

The results of this limit test conducted according to OECD guidelines, with a deviation of 2 weeks instead of 28 days as accepted by the FDA, are consistent with previous results showing the safety of pure C60 in rodents as confirmed by several independent research laboratories (Moussa et al., 1996[37]; Mori et al., 2006[35]; Henry et al., 2007[19]; Spohn et al., 2009[47]; Baati et al., 2012[3]). 

Taken together these results clearly show that oral administration for 14 consecutive days of C60 dissolved in EVO at approximately 4 (3.8) mg/kg bw/day dose did not cause any adverse effects in male or female Han:WIST rats. This dose level has already been the subject of a pharmacokinetic study in rats (Baati et al., 2012[3]), but to our knowledge, its acute oral toxicity has never been investigated in an accredited laboratory.

The NOAEL for C60 in soluble form cannot be determined under these conditions because of the solubility limit of C60 in olive oil (Baati et al., 2012[3]) and the maximum volume that can be administered to a rodent according to OECD (2008[39]) and US FDA guidelines (2007[51]). We can only learn from the results of this study is that the No Observed Adverse Effect Level (NOAEL) for EVO-C60 is necessarily higher than 3.8 mg/kg bw/day.

In order to administer higher doses of C60, the only option is to administer C60 in solid form, such as aqueous or oily suspensions (Moussa et al., 1996[37]; Gharbi et al., 2005[14]; Mori et al., 2006[35]). However, although it has been shown that nanoparticles or even microparticles of C60 can cross membrane cells after intraperitoneal administration of high doses (> 2000 mg/kg bw) to rodents (Moussa et al., 1996[37]; Gharbi et al., 2005[14]), the pharmacokinetics and biodistribution of C60 suspensions after oral administration of such high doses remain unknown to date (Mori et al., 2006[35]). Furthermore, this type of concentrated C60 suspensions in solid, micro- or nanoparticulate form is not intended for human consumption and it is unlikely that such preparations would be absorbed orally.

In this study, we focused only on the soluble form of C60 as it is offered online for human consumption (Keykhosravi et al., 2019[25]; Kerna et al., 2020[24]). In addition, we already have some basic information on the pharmacokinetics and biodistribution of soluble C60 in rats. In particular, it has been shown that C60 in soluble form can be absorbed through the gastrointestinal tract (Baati et al., 2012[3]). It has also been shown that C60 in soluble form, i.e. when its unsaturated bonds are accessible, is at least 100 times more active against oxidative stress (Baati et al., 2012[3]) than in solid form (Gharbi et al., 2005[14]).

The data from this certified toxicity study should put an end to the debate about the acute toxicity of pure naked C60, and, above all, pave the way for further preclinical trials. 

The outcome of this study is only a first step in addressing the uncertainties surrounding the safety of C60 (Kerna et al., 2020[24]; SCCS, 2023[43]) and obtaining regulatory approval before considering the use of this product for human consumption. The resulting dose of 3.8 mg/kg should serve as a starting point for completing preclinical studies, including genotoxicity and long-term effects of C60 in soluble form, before considering clinical trials with thorough dose translation from rodents to humans (US CDER, 2005[50]). This is the only way to know if these molecules, which have been so promising for almost 30 years, deserve to be considered in the biomedical and/or veterinary fields.

## Declaration

### Author contributions

CB, RW, and FM conceived the design of the study. CB, RW, FP, MDSP, and FM analyzed the data. CB, RW, and FM wrote the paper.

### Funding

Provided by SES Research Inc.

### Competing interest

Christopher Burres and Robert Wong are co-owners of SES Research, a company that profits from the sale of ESS60. Fabio Pedreira and Maria Da Silva Pimenta are employed at SES Research.

Fathi Moussa declares that his collaboration with the company SES Research in this toxicity study was free of charge and without any compensation.

### Data and materials availability

All data are available in the main text or the supplementary information.

### Supplementary information

Supplementary information includes the official report from the certified European Laboratory that conducted the entire study. This includes the raw data, details of the experimental procedures, and legal certifications related to the study.

## Supplementary Material

Supplementary information

## Figures and Tables

**Table 1:**
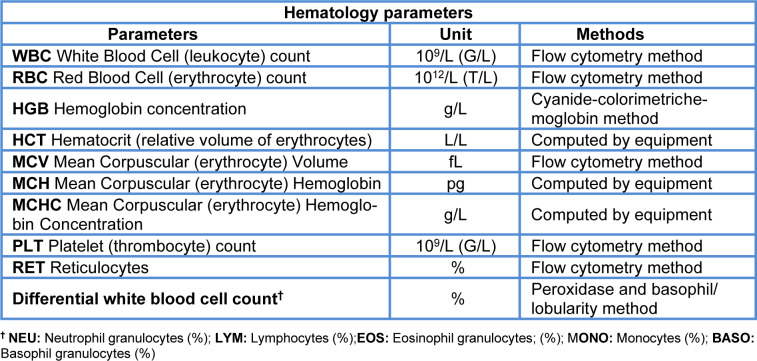
Hematology parameters (OECD, 2008)

**Table 2:**
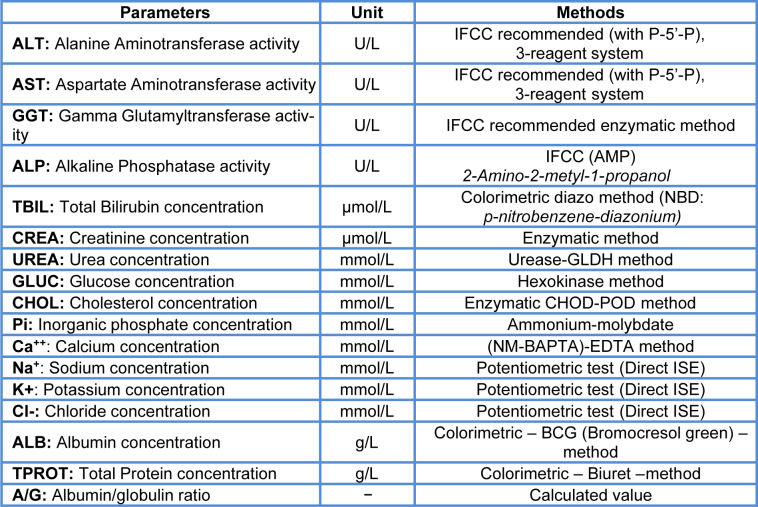
Clinical chemistry parameters (OECD, 2008) and methods

**Table 3:**
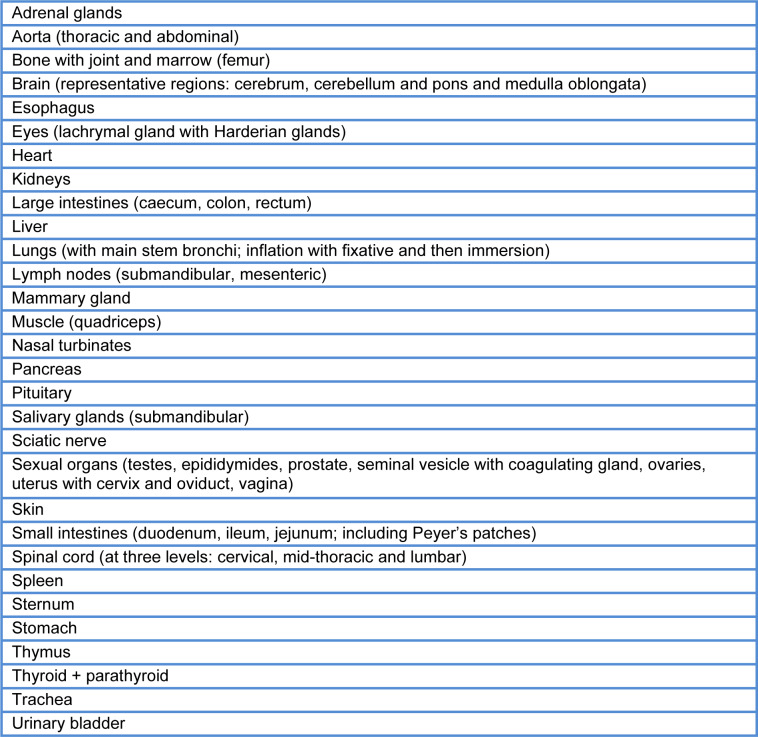
List of organs and tissues according to OECD guidelines (2008)

**Table 4:**
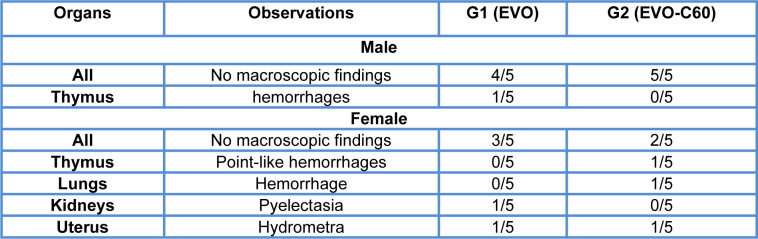
Summary of necropsy observations

## References

[1] Andrievsky GV, Bruskov VI, Tykhomyrov AA, Gudkov SV (2009). Peculiarities of the antioxidant and radioprotective effects of hydrated C60 fullerene nanostuctures in vitro and in vivo. Free Radic Biol Med.

[2] Arbogast JW, Darmanyan AO, Foote CS, Rubin Y, Diederich FN, Alvarez MM (1991). Photophysical properties of sixty atom carbon molecule (C60). J Phys Chem.

[3] Baati T, Bourasset F, Gharbi N, Njim L, Abderrabba M, Kerkeni A (2012). The prolongation of the lifespan of rats by repeated oral administration of [60]fullerene. Biomaterials.

[4] Beyaz S, Aslan A, Gok O, Ozercan IH, Agca CA (2023). Fullerene C60 protects against 7,12-dimethylbenz [a] anthracene (DMBA) induced-pancreatic damage via NF-?B and Nrf-2/HO-1 axis in rats. Toxicol Res.

[5] Cagle DW, Kennel SJ, Mirzadeh S, Alford JM, Wilson LJ (1999). In vivo studies of fullerene-based materials using endohedral metallofullerene radiotracers. Proc Natl Acad Sci U S A.

[6] Chen D, Liu S, Chen D, Liu J, Wu J, Wang H (2021). A two-pronged pulmonary gene delivery strategy: a surface-modified fullerene nanoparticle and a hypotonic vehicle. Angew Chem Int Ed.

[7] Diederich F, Thilgen C (1996). Covalent fullerene chemistry. Science.

[8] Dugan LL, Turetsky DM, Du C, Lobner D, Wheeler M, Almli CR (1997). Carboxyfullerenes as neuroprotective agents. Proc Natl Acad Sci U S A.

[9] Đurašević S, Nikolić G, Todorović A, Drakulić D, Pejić S, Martinović V (2020). Effects of fullerene C60 supplementation on gut microbiota and glucose and lipid homeostasis in rats. Food Chem Toxicol.

[10] Friedman SH, DeCamp DL, Sijbesma RP, Srdanov G, Wudl F, Kenyon GL (1993). Inhibition of the HIV-1 protease by fullerene derivatives: Model building studies and experimental verification. J Am Chem Soc.

[11] Galvan YP, Alperovich I, Zolotukhin P, Prazdnova E, Mazanko M, Belanova A (2017). Fullerenes as anti-aging antioxidants. Curr Aging Sci.

[12] Gao J, Wang Y, Folta KM, Krishna V, Bai W, Indeglia P (2011). polyhydroxy fullerenes (fullerols or fullerenols): beneficial effects on growth and lifespan in diverse biological models. PloS One.

[13] Geng H, Chang YN, Bai X, Liu S, Yuan Q, Gu W (2017). Fullerenol nanoparticles suppress RANKL-induced osteoclastogenesis by inhibiting differentiation and maturation. Nanoscale.

[14] Gharbi N, Pressac M, Hadchouel M, Szwarc H, Wilson SR, Moussa F (2005). [60]Fullerene is an in vivo powerful antioxidant with no acute or sub-acute toxicity. Nano Lett.

[15] Grebinyk A, Prylutska S, Grebinyk S, Prylutskyy Y, Ritter U, Matyshevska O, Kesharwani P (2023). Toward photodynamic cancer chemotherapy with C60-Doxorubicin nanocomplexes. Nanomaterials for photodynamic therapy. A volume in Woodhead Publishing Series in Biomaterials.

[16] Grohn KJ, Moyer BS, Wortel DC, Fisher CM, Lumen E, Bianchi, AH (2021). C60 in olive oil causes light-dependent toxicity and does not extend lifespan in mice. Geroscience.

[17] Haddon RC (1993). Chemistry of the fullerenes: the manifestation of strain in a class of continuous aromatic molecules. Science.

[18] Heflich LW (2023). Carbon 60: Vision for the future. Acta Sci Nutr Health.

[19] Henry TB, Menn FM, Fleming JT, Wilgus J, Compton RN, Sayler GS (2007). Attributing effects of aqueous C60 nano-aggregates to tetrahydrofuran decomposition products in larval zebrafish by assessment of gene expression. Environ Health Perspect.

[20] Inui S, Aoshima H, Nishiyama A, Itami S (2011). Improvement of acne vulgaris by topical fullerene application: unique impact on skin care. Nanomedicine.

[21] Jensen AW, Wilson SR, Schuster DI (1996). Biological applications of fullerenes. Bioorg Med Chem.

[22] Kawasaki R, Kondo K, Miura R, Yamana K, Isozaki H, Shimada R (2022). Theranostic agent combining fullerene nanocrystals and gold nanoparticles for photoacoustic imaging and photothermal therapy. Int J Mol Sci.

[23] Kepley CL (2012). Fullerenes in medicine;will it ever occur?. J Nanomed Nanotechol.

[24] Kerna NA, Flores JV, Pruitt KD, Nwokorie U, Holets H (2020). The application of fullerene derivatives in human nutrition: brain health, immunity, longevity, quality of life, skin tone, sports performance, vitality, and weight loss. EC Nutrition.

[25] Keykhosravi S, Rietveld IB, Couto D, Tamarit JL, Barrio M, Céolin R (2019). [60]Fullerene for medicinal purposes, a purity criterion towards regulatory considerations. Materials.

[26] Kipen HM, Laskin DL (2005). Smaller is not always better: nanotechnology yields nanotoxicology. Am J Physiol Lung Cell Mol Physiol.

[27] Kolosnjaj J, Szwarc H, Moussa F (2007). Toxicity studies of fullerenes and derivatives. Adv Exp Med Biol.

[28] Kroto HW, Heath JR, O’Brien SC, Curl RF, Smalley RE (1985). C60: Buckminsterfullerene. Nature.

[29] Krusic PJ, Wasserman E, Keizer PN, Morton JR, Preston KF (1991). Radical reactions of C60. Science.

[30] Kubota R, Tahara M, Shimizu K, Sugimoto N, Hirose A, Nishimura T (2011). Time dependent variation in the biodistribution of C60 in rats determined by liquid chromatography tandem mass spectrometry. Toxicol Lett.

[31] Li X, Deng R, Li J, Li H, Xu Z, Zhang L (2023). Oral [60]fullerene reduces neuroinflammation to alleviate Parkinson’s disease via regulating gut microbiome. Theranostics.

[32] Malhotra N, Audira G, Castillo AL, Siregar P, Ruallo JMS, Roldan MJ (2021). An update report on the biosafety and potential toxicity of fullerene-based nanomaterials toward aquatic animals. Oxid Med Cell Longev.

[33] Martínez-Herrera M, Figueroa-Gerstenmaier S, López-Camacho PY, Millan-Pacheco C, Balderas-Altamirano MA, Mendoza-Franco G (2022). Multiadducts of C60 modulate amyloid-β fibrillation with dual acetylcholinesterase inhibition and antioxidant properties: in vitro and in silico studies. J Alzheimers Dis.

[34] Masyutin AG, Erokhina MV, Shipelin VA, Gmoshinsky IV, Onishchenko GE (2020). Short-term introduction of fullerene C60 nanoparticles in rat small intestine induces the rapid development of hepatocyte pathology. Nanotechnol Russia.

[35] Mori T, Takada H, Ito S, Matsubayashi K, Miwa N, Sawaguchi T (2006). Preclinical studies on safety of fullerene upon acute oral administration and evaluation for no mutagenesis. Toxicology.

[36] Moussa F, Chrétien P, Dubois P, Chuniaud L, Dessante M, Trivin F (1995). The influence of C60 powders on cultured human leukocytes. Fullerene Sci Technol.

[37] Moussa F, Trivin F, Céolin R, Hadchouel M, Sizaret PY, Greugny V (1996). Early effects of C60 administration in Swiss mice: a preliminary account for in vivo C60 toxicity. Fullerene Sci Techno.

[38] Nozdrenko D, Matvienko T, Vygovska O, Bogutska K, Motuziuk O, Nurishchenko N (2021). Protective effect of water-soluble C60 fullerene nanoparticles on the ischemia-reperfusion injury of the muscle soleus in rats. Int J Mol Sci.

[39] OECD (2008). OECD Guidelines for Testing of Chemicals, Section 4 Health Effects;No 407,”Repeated dose 28-day oral toxicity study in rodents” (adopted 03 October 2008). https://www.oecd.org/chemicalsafety/testing/Revision-OECD-TG408-repeated-dose-90-day-oral-toxicity-study-in-rodents.pdf.

[40] Quick KL, Ali SS, Arch R, Xiong C, Wozniak D, Dugan LL (2008). A carboxyfullerene SOD mimetic improves cognition and extends the lifespan of mice. Neurobiol Aging.

[41] Ren L, Jing Z, Xia F, Zhang JZ, Li Y (2022). Toxic effect of fullerene and its derivatives upon the transmembrane β2 -adrenergic receptors. Molecules.

[42] Ryan J, Bateman HR, Stover A, Gomez G, Lenk R, Kepley CL (2007). Fullerene nanomaterials inhibit the allergic response. J Immunol.

[43] SCCS, Scientific Committee on Consumer Safety (2023). Opinion on Fullerenes, hydroxylated fullerenes and hydrated forms of hydroxylated fullerenes (nano). SCCS/1649/23. 2023. https://health.ec.europa.eu/system/files/2023-11/sccs_o_271.pdf.

[44] Shershakova N, Baraboshkina E, Andreev S, Purgina D, Struchkova I, Kamyshnikov O (2016). Anti-inflammatory effect of fullerene C60 in a mice model of atopic dermatitis. J Nanobiotechnol.

[45] Shytikov D, Shytikova I, Rohila D, Kulaga A, Dubiley T, Pishel I (2021). Effect of long-term treatment with C 60 fullerenes on the lifespan and health status of CBA/Ca mice. Rejuvenation Res.

[46] Sinegubova EO, Kraevaya OA, Volobueva AS, Zhilenkov AV, Shestakov AF, Baykov SV, el al (2023). Water-soluble fullerene C60 derivatives are effective inhibitors of influenza virus replication. Microorganisms.

[47] Spohn P, Hirsch C, Hasler F, Bruinink A, Krug HF, Wick P (2009). C60 fullerene: a powerful antioxidant or a damaging agent? The importance of an in-depth material characterization prior to toxicity assays. Environ Pollut.

[48] Stetska VO, Dovbynchuk TV, Makedon YS, Dziubenko NV (2021). The effect of water-soluble pristine C60 fullerene on 6-OHDA-induced Parkinson’s disease in rats. Regul Mech Biosyst.

[49] Tokuyama H, Yamago S, Nakamura E (1993). Photoinduced biochemical activity of fullerene carboxylic. J Am Chem Soc.

[50] US CDER. Department of Health and Human Services, Food and Drug Administration, Center for Drug Evaluation and Research (2005). Pharmacology and toxicology guidance for industry estimating the maximum safe starting dose in initial clinical trials for therapeutics in adult healthy volunteers. https://www.fda.gov/regulatory-information/search-fda-guidance-documents/estimating-maximum-safe-starting-dose-initial-clinical-trials-therapeutics-adult-healthy-volunteers.

[51] US FDA (2007). Toxicological principles for the safety assessment of food ingredients, Redbook 2000. Rev. July 2007. https://www.fda.gov/files/food/published/Toxicological-Principles-for-the-Safety-Assessment-of-Food-Ingredients.pdf.

[52] US NNI (2003). National Nanotechnology Initiative. https://www.nano.gov/national-nanotechnology-initiative.

[53] Ye L, Kollie L, Liu X, Guo W, Ying X, Zhu J (2021). Antitumor activity and potential mechanism of novel fullerene derivative nanoparticles. Molecules.

[54] Zhou Z, Lenk R, Dellinger A, MacFarland D, Kumar K, Wilson SR (2009). Fullerene nanomaterials potentiate hair growth. Nanomedicine.

