# Supplementary material for: A regulatory compliant short-term oral toxicity study of soluble [60]fullerenes in rats
Source: EXCLI J. 2024 May 15;23:772–86. doi: 10.17179/excli2024-7084 (PMC11231456; doi:10.17179/excli2024-7084)
Supplement: Supplementary information [file EXCLI-23-772-s-001.pdf]

EXCLI Journal 2024;23:772-786 – ISSN 1611-2156
Received: February 15, 2024, accepted: April 26, 2024, published: May 15, 2024

Supplementary information to:

Original article:

A REGULATORY COMPLIANT SHORT-TERM ORAL TOXICITY STUDY OF SOLUBLE [60]FULLERENES IN RATS

Christopher Burres[1], Robert Wong[1], Fabio Pedreira[1], Maria Da Silva Pimenta[1],
Fathi Moussa[2*]

[1] SES Research Inc., Houston, TX/USA
[2] Institut de Chimie Physique, CNRS – UMR 8000, Université Paris-Saclay; Gif-sur-Yvette 13 avenue des Sciences, 91190, France

* **Corresponding author:** Fathi Moussa, Institut de Chimie Physique, CNRS – UMR 8000,
Université Paris-Saclay; Gif-sur-Yvette 13 avenue des Sciences, 91190, France;
E-mail: fathi.moussa@universite-paris-saclay.fr

https://dx.doi.org/10.17179/excli2024-7084

This is an Open Access article distributed under the terms of the Creative Commons Attribution License (http://creativecommons.org/licenses/by/4.0/).

Preamble. This supplementary information includes the original official report of the certified European Laboratory that performed the entire study. This includes the details of the experimental procedures and all raw data as well as the legal attestations related to the study. While the study plan also includes C70 and a C60/C70 mixture, only the data concerning C60 must be considered.

TC

TOXI-COOP ZRT.

TOXI-COOP ZRT

Address: Berlini utca 47-49.
H-1045 Budapest
Hungary
Phone: +36-1-920-1228

Study Report

14-Day Repeated Dose Oral Gavage Toxicity Study of Fullerene C60 in Olive Oil in Rats

Study no: **842-400-5742**

Study Director: **Ilona Pasics Szakonyiné**

Date of Study Report: **April 14, 2021**

(Study Report including Appendices total pages 143)

Sponsor:

SES RESEARCH Inc.
5999 West 34th Street
Suite 106
Houston, TX 77079
USA

Test facility

Toxi-Coop Zrt.
Berlini utca 47-49.
H-1045 Budapest
Hungary

Table of Contents Page

Statement of the Study Director 6
Statement of the Management 7
Statement of the Quality Assurance GLP 8
General statements 9
Experimental schedule 10
Responsibilities 11
Regulatory guidelines and test methods 12
GLP compliance 12
Archiving 12
1.0 Summary 14
2.0 Study objective and introduction 16
3.0 Materials and methods 16
3.1 Test items 16
3.1.1 Characteristics of test items 16
3.1.2 Identification, receipt 17
3.1.3 Formulation 17
3.1.4 Concentration check of the formulated test item 17
3.2 Control item 17
3.3 Characteristics of anesthetic 17
3.4 Test system 17
3.4.1 Animals 17
3.4.2 Reason for selection of species 18
3.4.3 Husbandry 18
3.4.3.1 Housing conditions 18
3.4.3.2 Food and water supply 18
3.4.4 Identification of animals 19
3.5 Experimental design 20
3.5.1 Route of administration and reason for the selection 20
3.5.2 Randomization 20
3.5.3 Dose levels 20
3.5.4 Justification of dose level selection 21
3.6 Duration of the experimental period 21
4.0 Description of the test procedure 21
4.1 Selection of animals 21
4.2 Administration of test item 21
4.3 Mortality 21
4.4 Clinical observations 21
4.5 Body weight and body weight gain 22

4.6 Food consumption measurement and feed efficiency ... 22
4.7 Clinical pathology examinations ... 22
4.7.1 Hematology ... 22
4.7.2 Blood coagulation ... 23
4.7.3 Clinical chemistry ... 23
4.8 Pathology ... 25
4.8.1 Necropsy ... 25
4.8.2 Organ weight ... 26
4.8.3 Histopathology ... 26

5.0 Evaluation of experimental data ... 26

6.0 Animal welfare ... 27

7.0 Amendment and deviation to the Study Plan ... 27
7.1 Amendment to the Study Plan ... 27
7.2 Deviations to the Study Plan ... 27

8.0 References ... 28

9.0 Results ... 29
9.1 Mortality ... 29
9.2 Clinical observations ... 29
9.3 Body weight and body weight gain ... 29
9.4 Food consumption ... 29
9.5 Feed efficiency ... 30
9.6 Hematology and blood coagulation ... 30
9.7 Clinical chemistry ... 30
9.8 Necropsy ... 31
9.9 Organ weight ... 31
9.10 Histopathology ... 32

10.0 Conclusion ... 32

Abbreviations ... 33

APPENDICES ... 34

Table of Contents of Appendices Page

**APPENDIX 1**	1.1 Summary of daily clinical observations....2 pages
	1.2 Summary of weekly clinical observations....2 pages
	1.3 Individual daily clinical observations....2 pages
	1.4 Individual weekly clinical observations....2 pages
**APPENDIX 2**	2.1 Summary of body weight and body weight gain....2 pages
	2.2 Individual body weight and body weight gain....2 pages
**APPENDIX 3**	3.1 Summary of food consumption....2 pages
	3.2 Summary of feed efficiency....2 pages
	3.3 Individual food consumption....2 pages
	3.4 Individual feed efficiency....2 pages
**APPENDIX 4**	4.1 Summary of hematology and blood coagulation....2 pages
	4.2 Individual data of hematology blood coagulation....2 pages
**APPENDIX 5**	5.1 Summary of clinical chemistry....2 pages
	5.2 Individual data of clinical chemistry....2 pages
**APPENDIX 6**	6.1 Summary of necropsy findings....2 pages
	6.2 Individual necropsy findings....2 pages
**APPENDIX 7**	7.1 Summary of organ weight....6 pages
	7.2 Individual organ weight....6 pages
**APPENDIX 8**	8.1 Summary of histopathology findings....2 pages
	8.2 Individual histopathology findings....8 pages
**APPENDIX 9**	Copy of the Certificates of Analysis....4 pages
**APPENDIX 10**	Copy of the Health Certificate of animals....2 pages
**APPENDIX 11**	Quality control of the diet....5 pages
**APPENDIX 12**	Quality control of the drinking water....3 pages
**APPENDIX 13**	Copy of the Certificate of Quality of the bedding material....1 page
**APPENDIX 14**	Historical control data....10 pages
**APPENDIX 15**	Copy of the Study Plan....29 pages
**APPENDIX 16**	Copy of the Good Laboratory Practice (GLP) Certificate....1 page

The following prints of this report are issued:

Paper prints:

Original 1 of 2	Archived at Toxi-Coop Zrt.
Original 2 of 2	Released to the sponsor

Electronic copy:

Electronic copy 1 of 1	An electronic copy in PDF format is released to the sponsor. The electronic file is an unaudited copy, generated after finalization of the report. The sponsor is reminded that PDF files are not sufficiently protected against modification. Therefore, Toxi-Coop Zrt. cannot take any responsibility for the content of the electronic copy. The sponsor is using the electronic copy on his own responsibility.

Statement of the Study Director

This study has been performed in accordance with the study plan and the regulations laid down in the Principles of Good Laboratory Practice (Hungarian Good Laboratory Practice Regulation: 42/2014 (VITI. 19.) EMMI decree of the Minister of Human Capacities which corresponds to the OECD GLP, ENV/MC/CHEM(98)17) except for formulation analysis.

Analytical control of dosing formulations was not performed within the scope of this study because the control and test items were administered in "ready to use" form and the sponsor provided the analytical certificates for each substance.

The following guidelines were taken into account when the study was designed:

- OECD Guidelines for Testing of Chemicals, Section 4 Health Effects; No. 407, *"Repeated Dose 28-Day Oral Toxicity Study in Rodents"* (adopted 03 October 2008)
- US FDA Toxicological Principles for the Safety Assessment of Food Ingredients, Redbook 2000, IV.C. 3. a. *Short-Term Toxicity Studies with Rodents* (2003).

I, the undersigned study director declare that this report constitutes a true record of the actions undertaken and the results obtained in the course of this study.

Signature: Szakonyiné Pasics Ilona
Ilona Pasics Szakonyiné

Date: April 14, 2021

Statement of the Management

According to the conditions of the research and development assignment between SES RESEARCH Inc. (as Sponsor) and Toxi-Coop Zrt. (as Test Facility) "14-Day Repeated Dose Oral Gavage Toxicity Study of three Fullerenes in Olive Oil - C60/C70, C60, C70 - in Rats" has been performed in laboratory of Toxi-Coop Zrt. as a GLP study.

Signature: ______________________ Date: April 14, 2021

Dr. Gábor Hirka

Statement of the Quality Assurance GLP

Study no: **842-400-5742**

Study title: **14-Day Repeated Dose Oral Gavage Toxicity Study of three Fullerenes in Olive Oil - C60/C70, C60, C70 - in Rats**

Test Items: **Olive Oil infuse with Carbon C60/C70**
Olive Oil infuse with Carbon C60
Olive Oil infuse with Carbon C70

This study as well as the final report was inspected by the Quality Assurance in compliance with the Principles of Good Laboratory Practice. This final report reflects the raw data obtained during the performance of the study.
Properly signed reports of the performed inspections were submitted to the study director and to the test facility management. The dates of such inspections and the dates of reporting inspection results are given below.

Date	Phase inspected	Date of report to the study director	Date of report to the management
January 29, 2021	Study Plan	January 29, 2021	January 29, 2021
February 09, 2021	Handling of Test Item	February 09, 2021	February 09, 2021
January 12, 2021	Hematology and blood coagulation (process based)	January 12, 2021	January 12, 2021
September 15, 2020	Clinical chemistry (process based)	September 15, 2020	September 15, 2020
February 01, 02, 08, 09, 2021	Histopathological processing (process based)	February 09, 2021	February 09, 2021
April 09, 2021	Draft Report	April 09, 2021	April 09, 2021
April 14, 2021	Final Report	April 14, 2021	April 14 , 2021

Signature: ____________________
Anett Szegner
Quality Assurance GLP

April 14, 2021
Date

General statements

Study title: **14-Day Dose Range Finding Oral Gavage Toxicity Study of Fullerene C60 in Olive Oil in Rats**

Study number: **842-400-5742**

Sponsor:
SES RESEARCH Inc.
5999 West 34th Street
Suite 106
Houston, TX 77079 USA

Sponsor's scientific monitor:
John R. Endres, ND
Chief Scientific Officer
Natural and Medicinal Products Research
AIBMR Life Sciences, Inc.
2800 E. Madison St. Suite 202
Seattle WA 98112

Test facility:
Toxi-Coop Zrt.
Berlini utca 47-49.
H-1045 Budapest
Hungary

Arácsi út 97.
H-8230 Balatonfüred,
Hungary

Experimental schedule

**Date of start of experimental phase:**	February 09, 2021
**Date of end of in-life phase:**	February 23, 2021
**Date of end of experimental phase:**	April 02, 2021

Pre-experimental period

Animal arrival:	February 04, 2021
Veterinary control/acclimatization:	February 04 – 08, 2021
Animal identification:	February 04, 2021
Body weight measurement:	February 05, 08, 2021
Clinical observations:	February 05, 08, 2021
Randomization:	February 08, 2021

Experimental period

Treatment period:	February 09 – 22, 2021
Body weight measurement:	February 09, 12, 16, 19, 22, 2021
	Before the necropsy: February 23, 2021
Food consumption measurement:	February 09, 16, 22, 2021
Clinical observation:	February 09 – 22, 2021, daily
	February 09, 16, 23, 2021, weekly
Blood sampling:	February 23, 2021
Necropsy:	February 23, 2021

Date of Draft Report:	April 09, 2021
Date of Final Report:	April 14, 2021

Responsibilities

Test facility management: **Dr. Gábor Hirka**
Phone: +36-1-920-1228
E-mail: gabor.hirka@toxicoop.com

Study director: **Ilona Pasics Szakonyiné**
Phone: +36-30-846-2665
E-mail: ilona.pasics@toxicoop.com

Head of Quality assurance unit: **Ildikó Hermann**
Phone: +36-1-920-1228
E-mail: ildiko.hermann@toxicoop.com

Sponsor's scientific monitor: **John R. Endres, ND**
Phone: (253) 286-2888
john@aibmr.com
http://www.aibmr.com

Histopathology: **Róbert Glávits, D.V.M., Ph.D., D.Sc.**
Histopathologist

Responsible personnel: Anett Szegner – QA
Anita Mayer – QA
Anikó Renkó – clinical pathology

The following additional staff members were involved in the study:
Marcell Madár, Tímea Csörge, Ibolya Bogdán, Irén Somogyi Háriné, Istvánné Horváth, Bálint Zsolt Juhari, Judit Kálmán, Aranka Kiss, Klára Fritz Kovácsné, Máté Madár, Anikó Légrádi-Maurer, Éva Láng-Szabó, Olga Szász, Erika Misku Vargáné

Regulatory guidelines and test methods

The study followed the procedures indicated by the following internationally accepted guidelines and recommendations:

- OECD Guidelines for Testing of Chemicals, Section 4 Health Effects; No. 407, "*Repeated Dose 28-Day Oral Toxicity Study in Rodents*" (adopted 03 October 2008)
- US FDA Toxicological Principles for the Safety Assessment of Food Ingredients, Redbook 2000, IV.C. 3.a. *Short-Term Toxicity Studies with Rodents* (2003).

GLP compliance

The study was performed according to GLP at the Sponsor's request because of authority purposes. The Principles of Good Laboratory Practice as specified by Hungarian and international legislations were followed (except for formulation analysis):

- Hungarian Good Laboratory Practice Regulation: 42/2014 (VIII. 19.) EMMI decree of the Minister of Human Capacities which corresponds to the OECD GLP, ENV/MC/CHEM(98)17).
- OECD Principles of GLP as revised in 1997, published in ENV/MC/CHEM (98)17); OECD, Paris, 1998

All procedures mentioned in the Study Plan are the subject of detailed standard operating procedures (SOPs), which are contained in the SOP manuals of the operating departments of Toxi-Coop Zrt.
The Quality Assurance conducted inspections of the study plan, various phases of the study, certain repetitive operations and the report is audited according to internal Standard Operating Procedures.

Archiving

The study documents and samples as listed below are archived according to the OECD GLP and to the Toxi-Coop Zrt.'s SOP-s in the archives of Toxi-Coop Zrt. Berlini utca 47-49. H-1045 Budapest, Hungary:

- the Study Plan for 15 years,
- one original Final Report for 15 years,
- one sample of the test item for 5 years,
- all raw data for 15 years,
- biological samples for 5 or 12 years,
  - organs and tissues preserved in 4 % buffered formaldehyde solution for 5 years
  - blocks and slides of organs and tissues for 12 years
- correspondence for 15 years,

For the first 5 years, archiving is included. Thereafter archiving occurs at additional costs of the Sponsor. After this period, the Sponsor will be notified to decide on further archiving to comply with current legal requirements.
After the retention time, all the archived materials listed above will be returned to the Sponsor or retained for a further period if agreed by a contract or destroyed on their behalf. None of the above cited documents or material will be discarded without the explicit written consent of the Sponsor.

At the end of the study, any remaining test item will be returned to the Sponsor or will be discarded, unless otherwise instructed by the Sponsor.

1.0 Summary

The objective of this study was to obtain first information on the toxic potential of fullerene C60, test item Olive Oil infuse with Carbon C60 in the groups of male and female rats likely to arise from repeated exposure to the test item over a 14-Day repeat-dose test period.

Four groups of Han:WIST rats consisting of five animals per group and sex were administered orally (by gavage) once daily with EVO Olive Oil (0 mg/mL fullerene), and Olive Oil Infuse with Carbon C60 (3.8 mg/kg bw/day, 0.76 mg/mL) approximately at a dose 4 mg/kg bw/day and at a concentration of 0.8 mg/mL, corresponding to a dosing volume of 5 mL/kg bw, for each group.

Analytical control of dosing formulations was not performed within the scope of this study as each substance was administered in “ready to use” form and the sponsor provided the analytical certificate for control and test items.

Animals were observed twice and the body weight was determined twice for each animal during the acclimatization period.

Animals were observed for mortality twice a day during the course of the study. General clinical observations were performed daily after the treatment and detailed clinical observations were conducted on Days 0, 7 and 14. Body weights were recorded twice weekly. The food consumption was determined weekly to coincide with body weight measurements during the study. Feed efficiency was calculated by weekly interval. Clinical pathology and gross pathology examinations were conducted on all animals one day after the last treatment on Day 14 (male and female). Selected organs were weighed. Full histopathological examinations were performed on all animals of the control and test item treated groups (Groups 1, 2, 3 and 4).

The results of this study were summarized as follows:

Mortality: There was no mortality in any groups.

Clinical observations: Test item related clinical signs were not detected in male or female animals in any group – control, and Olive Oil infuse with Carbon C60. The animals exhibited normal behavior and physical condition in all groups at the daily and weekly clinical observations.

Body weight and body weight gain: The body weight development was not affected by the treatment of test items. The body weight and body weight gain were comparable in all groups.

Food consumption and feed efficiency: The mean daily food consumption and feed efficiency were similar in male and female in the control and all test items treated groups.

Hematology and blood coagulation: Test items related adverse effects were not identified in the examined hematological parameters in the male or female animals in any groups Olive Oil infuse with Carbon C60 with respect to their control.

Clinical chemistry: Pathological test items effects were not detected upon the evaluation of the clinical chemistry parameters in groups of Olive Oil infuse with Carbon C60.

Gross pathology: Specific macroscopic alterations indicative of test item effects were not observed in the organs and tissues of animals in any dosed groups.

Organ weight: Test item related effects were not detected in the weights of the examined organs in male or female animals in any test item administered dose groups.

Histopathology: No microscopic lesions related to test item were detected in any organs or tissues of animals subjected to histopathological examination (male and female) in groups of Olive Oil Infuse with Carbon C60 approximately at an approximately dose of 4 mg/kg bw/day

Conclusion

Under the condition of the present study, the fullerene C60 in Olive Oil caused no adverse effects in male or female Han:WIST rats after the consecutive 14-day oral (by gavage) administration at approximately 4 mg/kg bw/day dose.

Based on the observations made in this toxicity study the No Observed Adverse Effect Level (NOAEL) was determined for Olive Oil infuse with Carbon C60 as follows:

NOAEL: 4 mg/kg bw/day – male Han:WIST rats.

NOAEL: 4 mg/kg bw/day – female Han:WIST rats.

2.0 Study objective and introduction

The objective of this study was to obtain first information on the toxic potential of fullerene C60 i.e, test item Olive Oil infuse with Carbon C60 in male and femalerats likely to arise from repeated exposure to the test item over a 14-day repeat-dose test period.

3.0 Materials and methods

3.1 Test items

3.1.1 Characteristics of test items

Name of test item:	Olive Oil infuse with Carbon C60
Product Code:	SE20-142
Lot#:	V01561
Fullerene (C60) content:	830 mg/kg
Relative density:	0.91 g/mL
Appearance:	Reddish-brown- liquid
Odor:	Faint oil odor
Manufacturing date:	April 17, 2020
Expiry date:	April 17, 2023
Storage conditions:	At room temperature

3.1.2 Identification, receipt

The test item of a suitable chemical purity, analytical certificate, safety data sheet and specification of the product was supplied by the Sponsor.
All precautions required in the handling and disposal of the test item was outlined by the Sponsor. Identification of test item was made in Toxi-Coop Zrt. on the basis of the information included in the analytical certificate (see Appendix 9) and MSDS.

3.1.3 Formulation

Formulation of the test items was not necessary. The fullerene (Olive Oil infuse with Carbon C60 as well as the control item (EVO Olive Oil) were provided by the Sponsor in "ready to use" form.

3.1.4 Concentration check of the formulated test item

Analytical control of dosing formulations was not performed within the scope of this study. The control and test items were applied in "ready to use" form and the sponsor provided the analytical certificates for each substance.

3.2 Control item

Name:	EVO Olive Oil
Product Code:	SE20-EVOO
Lot#:	V100
Appearance:	Golden-greenish liquid
Odor:	Faint oil odor
Manufacturing date:	April 17, 2020
Expiry date:	April 17, 2023
Storage conditions:	At room temperature

3.3 Characteristics of anesthetic

Name:	Isoflurane CP®
Batch number:	G150G19A
Expiry date:	June 2024
Supplier:	Medicus Partner Kft.<br>2051 Biatorbágy, Tormásrét u. 12.<br>Hungary
Storage conditions:	Below 30 °C
Purpose of use:	Anesthesia during the blood collection and euthanasia

3.4 Test system

3.4.1 Animals

Species / Strain:	Han:WIST rat of Wistar origin
Source:	Toxi-Coop Zrt. 1103 Budapest, Cserkesz u. 90.

Hygienic level:	SPF (Specific pathogen-free) at arrival and kept in good conventional environment during the study.
Age of animals:	Male animals: 42 – 45 days,<br>Female animals: 41 – 43 days at start of the treatment;
Body weights:	170 – 184 g for male animals and<br>124 – 140 g for female animals at start of the treatment;<br>The weight variation did not exceed ± 20 percent of the mean weight.
Number and sex of animals:	20 rats (10 male and 10 female - nulliparous and non-pregnant animals)
Number of groups:	2 (1 test item treated groups + 1 control group)
Number of animals/groups:	10 (5 male; 5 female)
Animal health:	Only healthy animals were used for the study. Healthy status was certified by the breeder (Appendix 10).
Acclimatization time:	5 days

3.4.2 Reason for selection of species

The rat is commonly used species for toxicological studies in accordance with international recommendations.
The Wistar rat was the system of choice because it has been the preferred and mostcommonly used species for oral toxicity tests is a well-known laboratory model with sufficient historical data.

3.4.3 Husbandry

3.4.3.1 Housing conditions

Animal room no.:	18/1 and 18/2
Housing:	Individual caging
Cage type:	Type III polypropylene/polycarbonate
Bedding:	Certified laboratory wood bedding (SAFE 3/4-S-FASERN produced by J. Rettenmaier & Söhne GmbH+Co.KG; D-73494 Rosenberg Holzmühle 1 Germany; see Appendix 13). The cages and bedding were changed once or twice a week.
Illumination:	Artificial light, from 6 a.m. to 6 p.m. (except days of ophthalmology examinations)
Temperature:	22 ± 3 °C
Relative humidity:	30 - 70 %
Ventilation:	Above 10 air-exchanges/ hour by central air-condition system.

Environmental conditions were maintained by an air-condition system. Temperature and relative humidity were verified and recorded daily during the study.

3.4.3.2 Food and water supply

Animals received ssniff® SM R/M-Z+H complete diet for rats and mice produced by ssniff Spezialdiäten GmbH, D-59494 Soest Germany and tap water, as for human consumption, *ad libitum* except overnight food deprivation before the blood sampling.

The food was considered not to contain any contaminants that could reasonably be expected to affect the purpose or integrity of the study. The supplier provided an analytical certificate of the standard diet for the batch used. Contents of the standard diet for rats and mice guaranteed by the supplier are presented in Appendix 11.
Animals received tap water from watering bottles. Water quality control analysis and microbiological assessment are performed once in every six months by Government Office of Capital Budapest Department of Public Health and Medical Officer Service (Váci út 172-174. Budapest, H-1138 Hungary). The quality control results are available at Toxi-Coop Zrt.'s archives (see Appendix 12).

3.4.4 Identification of animals

Animals were identified by unique numbers. The individual identification was performed by a marker pen on the tail. Identification numbers were given for each animal on the basis of the master file of Toxi-Coop Zrt. and numbers were re-marked as necessary to ensure correct identification.
According to allocation into the treatment groups after randomization, animal numbers were as follows:

Table 1: Identification numbers of animals per groups

GROUPS	DOSE (mg/kg bw/day)	MALES	FEMALES
Group 1	0	9656<br>9657<br>9658<br>9662<br>9673	9681<br>9691<br>9697<br>9698<br>9701
Group 2	600	9655<br>9660<br>9666<br>9672<br>9679	9684<br>9689<br>9690<br>9692<br>9695

The cages were marked by identity cards, with information about the study number, control or test item name, group number, serial number of test item, sex, cage number and individual animal numbers, start of the treatment, date of the necropsy. Boxes were arrangedin such a way that possible effects due to cage placement are minimized.

3.5 Experimental design

3.5.1 Route of administration and reason for the selection

The test item was administered orally via gavage. The route of application was selected in compliance with international guidelines (See references in paragraph "Regulatory guidelines and test methods"). The oral route is the anticipated route of human exposure to the test item.

3.5.2 Randomization

Animals were randomly assigned to test groups. All animals were sorted according to body weight by computer and grouped according to weight ranges. There were an equal number of animals from each weight group in each of the experimental groups during the randomization.
The grouping was controlled by SPSS/PC+ computer program according to the actual body weight verifying the homogeneity and deviations among the groups.

3.5.3 Dose levels

A control and one dose groups were involved in the study. Table below contains the group number, doses, dosing volume and number of animals.

Table 2: Experimental design

Group number	Name and concentration † of control or test items	Dose†† (mg/kg bw/day)	Dose volume (mL/kg bw)	Number of animals	
				Male	Female
Group 1	EVO Olive Oil<br>0 mg/mL	0	5	5	5
Group 2	Olive Oil Infuse with Carbon C60<br>0.76 mg/ml	4 (3.8)	5	5	5

† Concentrations by C60;

††Doses calculated by C60 concentration, respectively.Animals in Group 1 only received the control item, EVO Olive Oil.

3.5.4 Justification of dose level selection

The Sponsor, in consultation with the Study Director, selected the dose levels to target approximate exposures of 0 and 4 mg/kg bw/day of control and test item C60.

3.6 Duration of the experimental period

The experimental period involved 5 days of acclimatization, 14 days treatment and observation periods and necropsy on the following day (Day 14).
The day of first treatment is considered as Day 0 of examination.

4.0 Description of the test procedure

4.1 Selection of animals

Twenty (20) healthy rats (ten males and ten females) were used in the study. Animals were selected for this study on the basis of adequate body weight, a body weight within ± 20% of the mean within a sex and free from clinical signs of disease or injury. Selected rats were distributed by randomization according to stratification by body weight so that there was no statistically significant difference among group body weight means within a sex.

4.2 Administration of test item

The control and test item were administered to the appropriate animals by once daily oral gavage approximately the same time each day morning within a 2-3 hours period from Day 0 up to Day 13 (for a period of 14 days).
The actual treatment volume was calculated according to the most recent body weight. Animals were not treated on the day of gross pathology.
A treatment volume of 5 mL/kg body weight was applied to animals of each group.

4.3 Mortality

Animals were inspected for signs of morbidity and mortality twice daily (at the beginning and end of each working day). There was no early death during the course of the study.

4.4 Clinical observations

General clinical observations were made cage-side once a day, after treatment at approximately the same time.
On the day prior to the first treatment with the test item, and approximately once weekly thereafter, detailed observations were conducted while handling the animal on days that the animals are weighed and food consumption measurements are taken. Potential signs noted included but were not limited to: changes in skin, fur, eyes, and mucous membranes, occurrence of secretions and excretions and autonomic activity (e.g., lacrimation,piloerection, pupil size, and unusual respiratory pattern).

Likewise, changes in gait, posture and response to handling as well as the presence of clonic or tonic movements, stereotype activities (e.g., excessive grooming, repetitive circling), or bizarre behavior (e.g., self-mutilation, walking backwards) were considered. All observations were recorded.

4.5 Body weight and body weight gain

Individual body weights were recorded twice during the acclimation. The body weight of animals involved in the study was determined on Day 0 (prior to study start) and twice weekly (i.e., on Days 0, 3, 7, 10 and 13) with a precision of 1 g. The animals were also weighed immediately prior to sacrifice in order to calculate organ to body weight ratios. Individual body weight changes were calculated according to the days of measurements and for the study overall.

4.6 Food consumption measurement and feed efficiency

Food consumption was determined with the measurement of given and non-consumed diet with an accuracy of 1 g once weekly to coincide with body weight measurements (Days 0, 7 and 13). Food consumption was evaluated and reported by weekly interval for each group.
Feed efficiency was calculated and reported. Feed efficiency was calculated on the basis of the weekly body weight gain and food consumption.
All animals were fasted overnight prior to blood sampling.

4.7 Clinical pathology examinations

Clinical pathology examinations – including hematology, blood coagulation and clinical chemistry – were conducted at termination of the treatment (i.e., one day after the last treatment; on Day 14; male and female).
Animals were food deprived overnight (for approximately 16 hours) prior to blood collection.
Blood samples were harvested from the retro orbital venous plexus under Isoflurane CP® anesthesia.
Three samples were taken from each animal: one for hematology, one for determination of blood clotting times and the third one to obtain serum samples for clinical chemistry.

4.7.1 Hematology

Blood samples for hematology measurements were collected in tubes containing K_3EDTA (spray-dried; MiniCollect® 0.5 mL, manufactured by Greiner Bio-One International AG, Kremsmünster, Austria) and tubes were filled up to the final volume marked on the tubes.
Analysis was performed immediately after sampling.
The parameters listed in Table 3 were measured by Siemens ADVIA120:

Table 3: Hematology parameters examined

PARAMETERS	UNIT	METHODS
**WBC**<br>White Blood Cell (leukocyte) count	10^9/L (G/L)	Flow cytometry method
**RBC**<br>Red Blood Cell (erythrocyte) count	10^{12}/L (T/L)	Flow cytometry method

Table 3: Hematology parameters examined

PARAMETERS	UNIT	METHODS
**HGB**<br>Hemoglobin concentration	g/L	Cyanide-colorimetric hemoglobin method
**HCT**<br>Hematocrit (relative volume of erythrocytes)	L/L	Computed by equipment
**MCV**<br>Mean Corpuscular (erythrocyte) Volume	fL	Flow cytometry method
**MCH**<br>Mean Corpuscular (erythrocyte) Hemoglobin	pg	Computed by equipment
**MCHC**<br>Mean Corpuscular (erythrocyte) Hemoglobin Concentration	g/L	Computed by equipment
**PLT**<br>Platelet (thrombocyte) count	10^9/L (G/L)	Flow cytometry method
**RET**<br>Reticulocytes,	%	Flow cytometry method
**Differential white blood cell count†**	%	Peroxidase and basophil/ lobularity method

* **NEU:** Neutrophil granulocytes (%); **LYM:** Lymphocytes (%);**EOS:** Eosinophil granulocytes; (%); M**ONO:** Monocytes (%); **BASO:** Basophil granulocytes (%);

4.7.2 Blood coagulation

Blood samples for determination of blood clotting times (APTT and PT) were collected in tubes containing 9NC Coagulation 3.8 % (MiniCollect® 1 mL; manufactured by Greiner Bio-One International AG, Kremsmünster, Austria). Tubes were filled up to the final volume marked on the tubes.
Blood was centrifuged at 2500 rpm for 15 minutes within 20 – 30 minutes after the sampling and supernatant plasma samples were measured immediately.
The following parameters were measured by AMAX Destiny Plus:

Table 4: Blood coagulation parameters examined

PARAMETERS	UNIT	METHODS
**APTT**<br>Activated partial Thromboplastin Time	sec	Optical
**PT**<br>Prothrombin Time	sec	Optical

4.7.3 Clinical chemistry

Blood samples collected for clinical chemistry measurements were drawn in tubes Vacuette 2.5 mL Z Serum Sep C/A (no anticoagulant; manufactured by Greiner Bio-One International AG, Kremsmünster, Austria). At least 1.0 mL blood was collected into clinical chemistry tubes. Samples were stored in a dark place at room temperature for 30-40 minutes and then centrifuged at 4500 rpm for 15 minutes. Serum samples were stored at 2-8 °C and measured.

The following parameters were measured in all animals by Cobas C311:

Table 5: Clinical chemistry parameters examined

PARAMETERS	UNIT	METHODS
**ALT**<br>Alanine Aminotransferase activity	U/L	IFCC recommended (with P-5'-P), 3-reagent system
**AST**<br>Aspartate Aminotransferase activity	U/L	IFCC recommended (with P-5'-P), 3-reagent system
**GGT**<br>Gamma Glutamyl transferase activity	U/L	IFCC recommended enzymatic method
**ALP**<br>Alkaline Phosphatase activity	U/L	IFCC (AMP) *2-Amino-2-metyl-1-propanol*
**TBIL**<br>Total Bilirubin concentration	µmol/L	Colorimetric diazo method (NBD: *p-nitrobenzene-diazonium)*
**CREA**<br>Creatinine concentration	µmol/L	Enzymatic method
**UREA**<br>Urea concentration	mmol/L	Urease-GLDH method
**GLUC**<br>Glucose concentration	mmol/L	Hexokinase method
**CHOL**<br>Cholesterol concentration	mmol/L	Enzymatic CHOD-POD method
**Pi**<br>Inorganic phosphate concentration	mmol/L	Ammonium-molybdate
**Ca^{++}**<br>Calcium concentration	mmol/L	(NM-BAPTA)-EDTA method
**Na^{+}**<br>Sodium concentration	mmol/L	Potentiometric test (Direct ISE)
**K^{+}**<br>Potassium concentration	mmol/L	Potentiometric test (Direct ISE)
**Cl^{-}**<br>Chloride concentration	mmol/L	Potentiometric test (Direct ISE)
**ALB**<br>Albumin concentration	g/L	Colorimetric - BCG (Bromocresol green) - method
**TPROT**<br>Total Protein concentration	g/L	Colorimetric – Biuret - method
**A/G**<br>Albumin/globulin ratio	–	Calculated value

4.8 Pathology

4.8.1 Necropsy

Gross pathology was performed on every experimental animal one day after the last treatment on Day 14 of the study.
Animals were anesthetized with Isoflurane CP® and were exsanguinated from the abdominal aorta after verification of narcosis.
The external appearance (surface of the body, all orifices) was examined, cranium, thoracic and abdominal cavities were opened and the appearance of the tissues and organs was observed macroscopically. All observations were recorded with details of the location, color, shape and size.
The following organs/tissues were removed and preserved in 4 % formaldehyde solution, except for testes and epididymides, which were preserved in modified Davidson solution and then stored in 4 % formaldehyde solution for histopathological examination:

Table 6: List of organs preserved

Adrenal glands
Aorta (thoracic and abdominal)
Bone with joint and marrow (femur)
Brain (representative regions: cerebrum, cerebellum and pons and medulla oblongata)
Esophagus
Eyes (lachrymal gland with Harderian glands)
Heart
Kidneys
Large intestines (caecum, colon, rectum)
Liver
Lungs (with main stem bronchi; inflation with fixative and then immersion;)
Lymph nodes (submandibular, mesenteric)
Mammary gland
Muscle (quadriceps)
Nasal turbinates
Pancreas
Pituitary
Salivary glands (submandibular)
Sciatic nerve
Sexual organs (testes, epididymides, prostate, seminal vesicle with coagulating gland, ovaries, uterus with cervix and oviduct, vagina)
Skin
Small intestines (duodenum, ileum, jejunum; including Peyer's patches)
Spinal cord (at three levels: cervical, mid-thoracic and lumbar)
Spleen
Sternum
Stomach
Thymus
Thyroid + parathyroid
Trachea
Urinary bladder

Thyroid and parathyroid were preserved together with larynx but larynx was not processed histologically.

Organs and tissues were excised, trimmed of any adherent tissue, as appropriate, weighed, and preserved as described above.

4.8.2 Organ weight

The following organs were weighed and recorded:

With precision of 0.01g:	Liver, kidneys, testes, epididymides, prostate, seminal vesicles with coagulating glands as a whole, uterus and fallopian tubes, thymus, spleen, brain and heart.
With precision of 0.001g:	Adrenal glands, ovaries

Paired organs were weighed together.

4.8.3 Histopathology

Full histological examinations were performed on the preserved organs and tissues of the animals from both the control and test item administered groups (Groups 1, and 2).

The fixed tissues were trimmed, processed, embedded in paraffin, sectioned with a microtome (at a thickness of 2-4 µm) placed on glass microscope slides, stained withhematoxylin and eosin and examined by light microscopy.

5.0 Evaluation of experimental data

Statistical analysis was done with SPSS PC+ software for the following data:

- Body weight
- Food consumption
- Feed efficiency
- Hematology
- Blood coagulation
- Clinical chemistry
- Organ weight

The heterogeneity of variance between groups was checked by Bartlett's homogeneity of variance test. Where no significant heterogeneity was detected, a one-way analysis of variance was carried out. If the obtained result was positive, Duncan's Multiple Range test was used to assess the significance of inter-group differences.

Where significant heterogeneity was found, the normal distribution of data was examined by Kolmogorov-Smirnov test. In case of a non-normal distribution, the non-parametric method of Kruskal-Wallis One-Way analysis of variance was used. If there was a positive result, the inter-group comparisons were performed using the Mann-Whitney U-test.

Frequency of clinical signs, pathological and histopathological findings by sex and dose was calculated.

The use of the word “significant” or “significantly” indicates a statistically significant difference between the control and the experimental groups. Significance was judged at a probability value of $p < 0.05$ and < 0.01. Male and female rats were evaluated separately.

6.0 Animal welfare

Institutional Animal Care and Use Committee (IACUC) of Toxi-Coop Zrt. permitted the conduct of the study by signature on the Study Plan. (SOP: ALT 023 - Instructions for animal protection)
The study was conducted according to the National Research Council. Guide for the Care and Use of Laboratory Animals and in compliance with the principles of the Hungarian Act 2011 CLVIII (modification of Hungarian Act 1998 XXVIII) and Government Decree 40/2013 regulating animal protection.

7.0 Amendment and deviation to the Study Plan

7.1 Amendment to the Study Plan

The Study Plan was not amended during the course of the study.

7.2 Deviations to the Study Plan

Item 1:	Experimental schedule
In the Study Plan:	Veterinary control/acclimatization: February 04 – 22, 2021<br>Body weight measurement: February 05 – 22, 2021<br>Clinical observations: February 05 – 22, 2021
Deviation:	Veterinary control/acclimatization: February 04 – 8, 2021<br>Body weight measurement: February 05 and 08, 2021<br>Clinical observations: February 05 – 8, 2021
Reason for the deviation:	Unrealized typographical errors regarding the date of the end of the pre- treatment period in the Study Plan.
Presumed effect on the study:	None.

8.0 References

1) OECD Principles of Good Laboratory Practice, adopted by Council on 26th November 1997; Environment Directorate, Organization for Economic Cooperation and Development, Paris 1998. (OECD Principles of GLP as revised in 1997, published in ENV/MC/CHEM (98)17); OECD, Paris, 1998)
2) Hungarian Good Laboratory Practice Regulation: 42/2014 (VIII. 19.) EMMI decree of the Minister of Human Capacities which corresponds to the OECD GLP, ENV/MC/CHEM(98)17).
3) OECD Guidelines for Testing of Chemicals, Section 4 Health Effects; No. 407, "*Repeated Dose 28-Day Oral Toxicity Study in Rodents*" (adopted 03 October 2008)
4) US FDA Toxicological Principles for the Safety Assessment of Food Ingredients, Redbook 2000, IV.C. 3. a. *Short-Term Toxicity Studies with Rodents* (2003).
5) National Research Council. Guide for the Care and Use of Laboratory Animals. Inst. Lab. Anim. Res., Comm. Life Sci., Natl. Acad. Press, 8th Edition, Washington, D.C., 2011.

9.0 Results

9.1 Mortality

There was no mortality at any of the tested groups – EVO Olive Oil (control), Olive Oil infuse with Carbon C60 – during the entire observation period.

(Appendices 1.1 and 1.2)

9.2 Clinical observations

Test item related clinical signs were not detected in animals of any groups (during the daily or weekly clinical observations.

Male animals in the control, Olive Oil infuse with Carbon C60 treated groups were symptom-free during the entire observation period.

There were no clinical signs in female animals in the control and Olive Oil infuse with Carbon C60 treated groups at the daily or at the detailed weekly clinical observations. The behavior and physical condition of these animals were considered to be normal at each dose level during the course of the 14-day observation period.

(Appendices 1.1, 1.2, 1.3 and 1.4)

9.3 Body weight and body weight gain

The body weight development was undisturbed in male and female animals in each group.

The mean body weight was comparable with their control in male and female animals in groups of Olive Oil infuse with Carbon C60 during the entire study.

(Appendices 2.1 and 2.2)

9.4 Food consumption

The mean daily food consumption was not affected by the fullerene treated groups – Olive Oil infuse with Carbon C60.

Statistical significance with respect to the control was detected at the slightly lower mean food consumption of male animals administered with Olive Oil infuse with Carbon C60 on week 2. This difference to control was minor therefore was considered to be toxicologically not relevant.

(Appendices 3.1 and 3.3)

9.5 Feed efficiency

The feed efficiency was comparable in male and female animals in the control and test item (Olive Oil infuse with Carbon C60) administered groups during the entire study.

(Appendices 3.2 and 3.4)

9.6 Hematology and blood coagulation

Test items related adverse effects were not identified in the examined hematological or blood coagulation parameters in the male or female animals in groups of Olive Oil infuse with Carbon C60.

(Appendices 4.1 and 4.2; historical control data are presented in Appendix 14)

9.7 Clinical chemistry

Specific pathologic changes were not detected in the examined clinical chemistry parameters.

Slightly lower mean concentration of glucose (GLUC) was detected in male animals in group 2 (Olive Oil infuse with Carbon C60).

In the female animals, the examined clinical chemistry parameters were comparable with the control in group 2 (Olive Oil infuse with Carbon C60).

These statistically significant differences with respect to the control in were probably not related to the test item. The glucose levels of male animalsin group 2 remained well within the historical control range. Therefore, these minor changes were judged to be indicative of biological variation and not related to the test item.

(Appendices 5.1 and 5.2; historical control data are presented in Appendix 14)

9.8 Necropsy

Fullerene C60 in Olive Oil did not induce specific macroscopic alterations in the tissues or organs of male or female animals.
Species specific changes (scar on the skin, pyelectasia, hydrometra and cyst in the uterus) and individual lesions (thymic hemorrhage, reddish-brown spot on the lung lobe) were detected in male and female animals as follows:

- lungs: hemorrhage: 1/5 female in group 2 – Olive Oil infuse with Carbon C60;
- hemorrhage in the thymus: 1/5 male in control group; 1/5 female in group 2 – Olive Oil infuse with Carbon C60;
- right or both sided pyelectasia:
  1/5 female in control group;
- moderate hydrometra: 1/5 female in control group; and 1/5 female in group 2 – Olive Oil infuse with Carbon C60;

The thymic hemorrhage and pulmonary findings were probably due to the exsanguination procedure and are frequently observed in experimental rats.

Pyelectasia is a common observation in experimental rats of this strain occurring also in not treated animals. This finding was observed in all dosed groups without any dose relevance.

Hydrometra related to the female sexual cycle, is a frequent observation in experimental rats.
In the lack of related histopathological alterations (inflammatory, necrotic or other pathological lesions) these findings were considered to be toxicologically not relevant in the present study.

(Appendices 6.1 and 6.2)

9.9 Organ weight

There were no test item related changes in the weights of the examined organs in the male or female animals in group of Olive Oil infuse with Carbon C60.

There were no statistically or biologically significant differences with respect to the control in the mean weight of the examined organs in male and female animals at the end of the 14-day observation period.

(Appendices 7.1 and 7.2; historical control data are presented in Appendix 14)

9.10 Histopathology

Histopathological examination did not reveal specific microscopic alterations related to the test item in the examined organs or tissues of animals (male or female).

The alveolar emphysema and the acute hemorrhages in the thymus and lungs occurred sporadically in control and treated animals (emphysema: 1/5 female in group 2; acute hemorrhage in the thymus 1/5 male in control group and 1/5 female in group 2; acute hemorrhage in the lungs: 1/5 female in group 2). These findings could be in connection with the hypoxia, dyspnea and circulation disturbance, developed during the exsanguinations.

The pyelectasia (one or both side) without other histological lesions (degeneration, inflammation, fibrosis etc.) is considered, as slight individual findings without pathological significance in laboratory rats and was observed in 1/5 female animals of the control group.

The dilatation of uterine horns in some female animals – 2/5 control and 1/5 in group 2 is a slight neuro-hormonal phenomenon in connection with the sexual function (pro-estrus phase) of the inner genital organs.

No morphological evidence of acute or subacute injury (degeneration, proliferation, inflammation, necrosis etc.) of the gastrointestinal tract, liver, pancreas, cardiovascular system, urinary system, lymphoid system, hematopoietic system, the skeleton, the muscular system, the male and female reproductive system or the central, or peripheral nervous system, the eyes, the lachrymal glands and the integumentary system was observed.

The structure and the cell morphology of the endocrine glands were identical in the control and treated animals.

(Appendices 8.1 and 8.2)

10.0 Conclusion

Under the condition of the present study, fullerene C60 in Olive Oil caused no adverse effects in male or female Han:WIST rats after the consecutive 14-day oral (by gavage) administration at approximately 4 mg/kg bw/day dose.

Based on the observations made in this toxicity study the No Observed Adverse Effect Level (NOAEL) was determined for Olive Oil infuse with Carbon C60 as follows:

NOAEL: 4 mg/kg bw/day – male Han:WIST rats.

NOAEL: 4 mg/kg bw/day – female Han:WIST rats.

Abbreviations

Ad libitum	at will
APP.	Appendix
Bw/bw	Body weight
°C	Degrees centigrade
CD	Compact disk
GLP	Good Laboratory Practice
h	hour
mL	Milliliter
µg	Microgram
g	Gram
kg	Kilogram
Ltd	Limited Liability Corporation/Company
mg	Milligram
cm	Centimeter
Zrt.	Zártkörűen működő Részvénytársaság (Private Limited Company)
h	Hour(s)
min.	Minute(s)
N or n	Size of a statistical population
no	Number
OECD	Organisation for Economic Co-operation and Development
PDF	Portable Document Format
PhD	Doctor of Philosophy
PLtd. Co	Public Limited Company
Ref	Reference
QA	Quality Assurance
QC	Quality Control
RT	Room temperature
SOP	Standard Operating Procedures
SPF	Specific Pathogen Free
u	Utca (street)
%	Percentage
±	Plus/minus

Abbreviations of clinical pathology parameters are explained in paragraph 4.7 of this report.

APPENDICES

APPENDIX 1.1
Summary of daily clinical observations
Male

Observations	Control<br>EVO Olive Oil	4 (3.8) mg/kg bw/day<br>Olive Oil Infuse wit Carbon C60
Normal	5/5	5/5
Thin faces	0/5	0/5
Skin: Scars - left ear, neck	0/5	0/5

Remark: Frequency of observations: number of animals with observation/number of animals examined

APPENDIX 1.2

Summary of daily clinical observations
Female

**Observations**	**Control**<br>EVO Olive Oil	**4 (3.8) mg/kg bw/day**<br>Olive Oil Infuse wit Carbon C60
Normal	5/5	5/5

Remark: Frequency of observations: number of animals with observation/number of animals examined

APPENDIX 1.2
Summary of weekly clinical observations
Male

**Day of observations**	**Observations**	**Control**<br>EVO Olive Oil	**4 (3.8) mg/kg bw/day**<br>Olive Oil Infuse wit Carbon C60
Day 0	Normal	5/5	5/5
Day 7	Normal	5/5	5/5
Day 14	Normal	5/5	5/5
	Skin: Scars - left ear, neck	0/5	0/5

Remark: Frequency of observations: number of animals with observation/number of animals examined

APPENDIX 1.2
Summary of weekly clinical observations
Female

**Day of observations**	**Observations**	**Control**<br>EVO Olive Oil	**4 (3.8) mg/kg bw/day**<br>Olive Oil Infuse wit Carbon C60
Day 0	Normal	5/5	5/5
Day 7	Normal	5/5	5/5
Day 14	Normal	5/5	5/5

Remark: Frequency of observations: number of animals with observation/number of animals examined

APPENDIX 1.3

Individual daily clinical observations

Male

Group	Animal number	Observations	Duration of observations	Frequency of observations
**Control**	9656	Normal	Day 0 - Day 13	14
EVO Olive Oil	9657	Normal	Day 0 - Day 13	14
	9658	Normal	Day 0 - Day 13	14
	9662	Normal	Day 0 - Day 13	14
	9673	Normal	Day 0 - Day 13	14
**4 (3.8)**	9655	Normal	Day 0 - Day 13	14
**mg/kg bw/day**	9660	Normal	Day 0 - Day 13	14
Olive Oil Infuse with	9666	Normal	Day 0 - Day 13	14
Carbon C60	9672	Normal	Day 0 - Day 13	14
	9679	Normal	Day 0 - Day 13	14

APPENDIX 1.3
Individual daily clinical observations
Female

Group	Animal number	Observations	Duration of observations	Frequency of observations
**Control**	9681	Normal	Day 0 - Day 13	14
EVO Olive Oil	9691	Normal	Day 0 - Day 13	14
	9697	Normal	Day 0 - Day 13	14
	9698	Normal	Day 0 - Day 13	14
	9701	Normal	Day 0 - Day 13	14
**4 (3.8)**	9684	Normal	Day 0 - Day 13	14
**mg/kg bw/day**	9689	Normal	Day 0 - Day 13	14
Olive Oil Infuse with	9690	Normal	Day 0 - Day 13	14
Carbon C60	9692	Normal	Day 0 - Day 13	14
	9695	Normal	Day 0 - Day 13	14

APPENDIX 1.4

Individual weekly clinical observations

Male

Group	Animal number	Observations	Day of observations	Frequency of observations
**Control** EVO Olive Oil	9656	Normal	Day 0, Day 7, Day 14	3
	9657	Normal	Day 0, Day 7, Day 14	3
	9658	Normal	Day 0, Day 7, Day 14	3
	9662	Normal	Day 0, Day 7, Day 14	3
	9673	Normal	Day 0, Day 7, Day 14	3
**4 (3.8) mg/kg bw/day** Olive Oil Infuse with Carbon C60	9655	Normal	Day 0, Day 7, Day 14	3
	9660	Normal	Day 0, Day 7, Day 14	3
	9666	Normal	Day 0, Day 7, Day 14	3
	9672	Normal	Day 0, Day 7, Day 14	3
	9679	Normal	Day 0, Day 7, Day 14	3

APPENDIX 1.4
Individual weekly clinical observations
Female

Group	Animal number	Observations	Day of observations	Frequency of observations
**Control**	9681	Normal	Day 0, Day 7, Day 14	3
EVO Olive Oil	9691	Normal	Day 0, Day 7, Day 14	3
	9697	Normal	Day 0, Day 7, Day 14	3
	9698	Normal	Day 0, Day 7, Day 14	3
	9701	Normal	Day 0, Day 7, Day 14	3
**4 (3.8)**	9684	Normal	Day 0, Day 7, Day 14	3
**mg/kg bw/day**	9689	Normal	Day 0, Day 7, Day 14	3
Olive Oil Infuse with	9690	Normal	Day 0, Day 7, Day 14	3
Carbon C60	9692	Normal	Day 0, Day 7, Day 14	3
	9695	Normal	Day 0, Day 7, Day 14	3

APPENDIX 2.1

Summary of body weight and body weight gain
Male

Group		Body weight (g) on days					Body weight (g) between days				
		0	3	7	10	13	0-3	3-7	7-10	10-13	0-13
**Control** EVO Olive Oil	Mean	178.0	197.4	224.4	242.4	258.6	19.4	27.0	18.0	16.2	80.6
	SD	4.8	4.4	6.3	6.5	8.4	1.5	2.7	4.6	2.8	5.7
	n	5	5	5	5	5	5	5	5	5	5
**4 (3.85) mg/kg bw/day** Olive Oil infused with C60	Mean	178.0	198.4	222.0	237.6	250.2	20.4	23.6	15.6	12.6	72.2
	SD	6.1	6.5	9.5	10.0	12.8	3.0	3.4	1.1	4.2	8.9
	n	5	5	5	5	5	5	5	5	5	5
	%	0	1	-1	-2	-3					
		NS	NS	NS	NS	NS	NS	NS	NS	NS	NS

REMARKS: ±% = Percent Deviation Versus Control
NS = Not Significant
* = $p < 0.05$
** = $p < 0.01$
U = Mann-Whitney U - test Versus Control
DN = Duncan's multiple range test

Study no. 842-400-5742

APPENDIX 2.1

Summary of body weight and body weight gain
Female

Group		Body weight (g) on days					Body weight (g) between days				
		0	3	7	10	13	0-3	3-7	7-10	10-13	0-13
**Control** EVO Olive Oil	Mean	132.0	140.4	154.6	161.0	171.0	8.4	14.2	6.4	10.0	39.0
	SD	5.5	3.6	4.0	3.2	7.1	3.0	4.5	2.1	5.7	9.8
	n	5	5	5	5	5	5	5	5	5	5
**4 (3.85) Mg/kg bw/day** Olive Oil infused with C60	Mean	132.8	140.2	154.6	163.6	174.4	7.4	14.4	9.0	10.8	41.6
	SD	3.0	4.3	6.8	6.7	4.7	2.9	2.7	1.4	2.6	2.3
	n	5	5	5	5	5	5	5	5	5	5
	%	1	0	0	2	2					
		NS	NS	NS	NS	NS	NS	NS	NS	NS	NS

REMARKS: ±% = Percent Deviation Versus Control
NS = Not Significant
* = $p < 0.05$
** = $p < 0.01$
U = Mann-Whitney U - test Versus Control
DN = Duncan's multiple range test

APPENDIX 2.2

Individual body weight and body weight gain

Male

Group	Animal number	Body weight (g) on days					Body weight (g) between days				
		0	3	7	10	13	0-3	3-7	7-10	10-13	0-13
**Control** EVO Olive Oil	9656	173	193	219	243	256	20	26	24	13	83
	9657	174	194	217	235	252	20	23	18	17	78
	9658	184	201	228	246	263	17	27	18	17	79
	9662	182	203	232	251	271	21	29	19	20	89
	9673	177	196	226	237	251	19	30	11	14	74
**4 (3.85) mg/kg bw/day** Olive Oil infused with C60	9655	181	198	218	234	244	17	20	16	10	63
	9660	182	200	226	240	254	18	26	14	14	72
	9666	173	196	219	235	242	23	23	16	7	69
	9672	184	208	236	253	271	24	28	17	18	87
	9679	170	190	211	226	240	20	21	15	14	70

APPENDIX 2.2

Individual body weight and body weight gain

Female

Group	Animal number	Body weight (g) on days					Body weight (g) between days				
		0	3	7	10	13	0-3	3-7	7-10	10-13	0-13
**Control** EVO Olive Oil	9681	126	138	151	159	170	12	13	8	11	44
	9691	128	136	152	158	167	8	16	6	9	39
	9697	134	140	159	162	180	6	19	3	18	46
	9698	132	143	159	166	176	11	16	7	10	44
	9701	140	145	152	160	162	5	7	8	2	22
**4 (3.85) mg/kg bw/day** Olive Oil infused with C60	9684	134	144	162	172	179	10	18	10	7	45
	9689	128	137	148	156	168	9	11	8	12	40
	9690	136	145	161	168	179	9	16	7	11	43
	9692	130	135	148	158	172	3	13	10	14	40
	9695	128	140	154	164	174	6	14	10	10	40

APPENDIX 3.1
Summary of food consumption

Male

Group		Food consumption (g/animal/day) Between days 0 - 7	7 – 13
**Control** EVO Olive Oil	Mean	22.3	22.5
	SD	0.9	1.1
	n	5	5
**4 (3.85) mg/kg bw/day** Olive Oil infused with C60	Mean	21.0	20.0
	SD	1.0	1.2
	n	5	5
	± %	-6	-11 *
		NS	DN

REMARKS: ±% = Percent Deviation Versus Control
NS = Not Significant
* = p < 0.05
** = p < 0.01
U = Mann-Whitney U - test Versus Control
DN = Duncan's multiple range test

APPENDIX 3.1

Summary of food consumption

Female

Group		Food consumption (g/animal/day) Between days 0 – 7	7 - 13
**Control**	Mean	14.5	14.5
EVO Olive Oil	SD	0.3	0.7
	n	5	5
**4 (3.85)**	Mean	15.0	14.2
**mg/kg bw/day**	SD	1.1	0.6
Olive Oil infused with	n	5	5
C60	± %	3	-2
		NS	NS

Remarks:
±% = Percent Deviation Versus Control
NS = Not Significant
* = $p < 0.05$
** = $p < 0.01$
U = Mann-Whitney U - test Versus Control
DN = Duncan's multiple range test

APPENDIX 3.2
Summary of feed efficiency

Male

Group		Feed efficiency (g food/g bwg)		
	**Days**	0 – 7	7 – 13	0-13
	**Weeks**	1	2	
**Control** EVO Olive Oil	Mean	3.38	4.03	3.62
	SD	0.13	0.70	0.20
	n	5	5	5
**4 (3.85) mg/kg bw/day** Olive Oil infused with C60	Mean	3.37	4.31	3.72
	SD	0.35	0.49	0.29
	n	5	5	5
		NS	NS	NS

Remarks: Feed efficiency = food consumption/ body weight gain
NS = Not Significant
* = p < 0.05
** = p < 0.01
U = Mann-Whitney U - test Versus Control
DN = Duncan's multiple range test
bwg = body weight gain

APPENDIX 3.2

Summary of feed efficiency

Female

Group		Feed efficiency (g food/g bwg)		
	**Days**	**0 – 7**	**7 – 13**	**0-13**
	**Weeks**	**1**	**2**	
**Control** EVO Olive Oil	Mean	4.90	5.61	5.18
	SD	1.88	1.48	1.68
	n	5	5	5
**4 (3.85) mg/kg bw/day** Olive Oil infused with C60	Mean	4.95	4.37	4.57
	SD	0.93	0.64	0.16
	N	5	5	5
		NS	NS	NS

REMARKS: Feed efficiency = food consumption/ body weight gain
NS = Not Significant
* = $p < 0.05$
** = $p < 0.01$
U = Mann-Whitney U - test Versus Control
DN = Duncan's multiple range test
bwg = body weight gain

APPENDIX 3.3

Individual food consumption

Male

Group	Cage number	Animal number	Given food (g) Day 0	Given food (g) Day 7	Remained food (g) Day 7	Remained food (g) Day 13	Food consumption (g/animal/day) Between days 0 - 7	Food consumption (g/animal/day) Between days 7 - 13
**Control** EVO Olive Oil	1	9656	400	400	250	260	21	23
	2	9657	400	400	247	272	22	21
	3	9658	400	400	247	270	22	22
	4	9662	400	400	236	256	23	24
	5	9673	400	400	238	268	23	22
**4 (3.85) mg/kg bw/day** Olive Oil infused with C60	11	9655	400	400	254	286	21	19
	12	9660	400	400	252	278	21	20
	13	9666	400	400	255	284	21	19
	14	9672	400	400	242	269	23	22
	15	9679	400	400	262	284	20	19

APPENDIX 3.3

Individual food consumption

Female

Group	Cage number	Animal number	Given food (g)		Remained food (g)		Food consumption (g/animal/day) Between days	
			Day 0	Day 7	Day 7	Day 13	0 - 7	7 - 13
**Control** EVO Olive Oil	21	9681	400	400	297	312	15	15
	22	9691	400	400	298	310	15	15
	23	9697	400	400	299	310	14	15
	24	9698	400	400	296	313	15	15
	25	9701	400	400	301	320	14	13
**4 (3.85) mg/kg bw/day** Olive Oil infused with C60	31	9684	400	400	287	314	16	14
	32	9689	400	400	304	319	14	14
	33	9690	400	400	287	310	16	15
	34	9692	400	400	296	314	15	14
	35	9695	400	400	302	317	14	14

APPENDIX 3.4
Individual feed efficiency

Male

Group	Animal number		Feed efficiency (g food/g bwg)		
		Days	0-7	7-13	0-13
		Weeks	1	2	1-2
**Control** EVO Olive Oil	9656		3.26	3.78	3.49
	9657		3.56	3.66	3.60
	9658		3.48	3.71	3.58
	9662		3.28	3.69	3.46
	9673		3.31	5.28	3.97
**4 (3.85) mg/kg bw/day** Olive Oil infused with C60	9655		3.95	4.38	4.13
	9660		3.36	4.36	3.75
	9666		3.15	5.04	3.78
	9672		3.04	3.74	3.32
	9679		3.37	4.00	3.63

Remark: bwg = body weight gain

APPENDIX 3.4

Individual feed efficiency

Female

Group	Animal number	Days / Weeks	Feed efficiency (g food/g bwg) 0-7 / 1	7-13 / 2	0-13 / 1-2
**Control** EVO Olive Oil	9681		4.12	4.63	4.34
	9691		4.25	6.00	4.92
	9697		4.04	4.29	4.15
	9698		3.85	5.12	4.34
	9701		8.25	8.00	8.14
**4 (3.85) mg/kg bw/day** Olive Oil infused with C60	9684		4.04	5.06	4.42
	9689		4.80	4.05	4.43
	9690		4.52	5.00	4.72
	9692		6.50	3.58	4.75
	9695		4.90	4.15	4.53

Remark: bwg = body weight gain

APPENDIX 4.1

Summary of hematology and blood coagulation

Male

		WBC [x10^9/L]	NEU [%]	LYM [%]	MONO [%]	EOS [%]	BASO [%]	RBC [x10^{12}/L]	HGB [g/L]	HCT [L/L]	MCV [fL]	MCH [pg]	MCHC [g/L]	PLT [x10^9/L]	RET [%]	PT [sec]	APTT [sec]
**Control**	Mean	6.96	19.44	75.68	2.48	1.98	0.06	7.60	151.0	0.44	58.02	20.00	344.4	973.4	3.32	10.36	11.08
EVO Olive Oil	SD	2.73	10.41	14.19	1.25	2.74	0.05	0.90	9.9	0.04	2.64	1.21	5.7	62.4	0.79	0.13	1.27
	n	5	5	5	5	5	5	5	5	5	5	5	5	5	5	5	5
**4 (3.8)**	Mean	6.74	13.04	84.02	1.78	0.70	0.06	7.79	159.2	0.47	60.54	20.48	338.4	964.8	3.20	10.28	11.92
**mg/kg bw/day**	SD	1.20	2.42	2.05	0.25	0.29	0.05	0.32	5.8	0.01	2.85	0.80	4.6	167.3	0.56	0.13	1.37
Olive Oil Infuse with	n	5	5	5	5	5	5	5	5	5	5	5	5	5	5	5	5
Carbon C60	±%	-3	-33	11	-28	-65	0	2	5	7	4	2	-2	-1	-4	-1	8
		NS	NS	NS	NS	NS	NS	NS	DN	DN	NS	NS	NS	NS	NS	NS	NS

REMARKS: ±% = Percent Deviation Versus Control
NS = Not Significant
* = $p < 0.05$
** = $p < 0.01$
U = Mann-Whitney U - test Versus Control
DN = Duncan's multiple range test

APPENDIX 4.1

Summary of hematology and blood coagulation

Female

		WBC [x10^9/L]	NEU [%]	LYM [%]	MONO [%]	EOS [%]	BASO [%]	RBC [x10^{12}/L]	HGB [g/L]	HCT [L/L]	MCV [fL]	MCH [pg]	MCHC [g/L]	PLT [x10^9/L]	RET [%]	PT [sec]	APTT [sec]
**Control**	Mean	6.60	13.90	82.44	2.32	0.90	0.04	7.95	155.8	0.45	57.00	19.56	343.0	1078.0	2.39	10.00	10.56
EVO Olive Oil	SD	1.40	3.07	2.98	0.68	0.34	0.05	0.25	6.3	0.02	1.68	0.61	2.1	157.1	0.26	0.23	0.43
	n	5	5	5	5	5	5	5	5	5	5	5	5	5	5	5	5
**4 (3.8)**	Mean	5.64	12.12	84.26	2.34	0.80	0.06	7.97	154.6	0.45	56.66	19.36	342.0	953.6	2.57	10.10	10.80
**mg/kg bw/day**	SD	0.86	4.55	5.29	1.39	0.29	0.05	0.22	5.3	0.01	0.71	0.43	6.7	118.3	0.38	0.12	0.51
Olive Oil Infuse with	n	5	5	5	5	5	5	5	5	5	5	5	5	5	5	5	5
Carbon C60	±%	-15	-13	2	1	-11	50	0	-1	-1	-1	-1	0	-12	8	1	2
		NS	NS	NS	NS	NS	NS	NS	NS	NS	NS	NS	NS	NS	NS	NS	NS

REMARKS: ±% = Percent Deviation Versus Control
NS = Not Significant
* = $p < 0.05$
** = $p < 0.01$
U = Mann-Whitney U - test Versus Control
DN = Duncan's multiple range test

APPENDIX 4.2
Individual data of hematology and blood coagulation
Male

		WBC [x10^9/L]	NEU [%]	LYM [%]	MONO [%]	EOS [%]	BASO [%]	RBC [x10^{12}/L]	HGB [g/L]	HCT [L/L]	MCV [fL]	MCH [pg]	MCHC [g/L]	PLT [x10^9/L]	RET [%]	PT [sec]	APTT [sec]
**Control**	9656	7.3	16.5	79.9	2.4	0.6	0.0	6.06	134	0.38	62.6	22.1	354	973	4.46	10.3	10.7
EVO Olive Oil	9657	7.3	13.8	84.1	1.3	0.5	0.1	7.91	155	0.45	57.2	19.6	342	964	3.40	10.2	10.6
	9658	2.3	36.3	52.2	4.5	6.8	0.1	7.98	156	0.46	57.5	19.6	341	1071	2.57	10.3	10.7
	9662	9.0	9.2	88.4	1.6	0.4	0.0	7.67	151	0.44	57.0	19.7	345	962	3.59	10.5	10.1
	9673	8.9	21.4	73.8	2.6	1.6	0.1	8.39	159	0.47	55.8	19.0	340	897	2.58	10.5	13.3
**4 (3.8)**	9655	8.2	16.3	81.1	1.6	0.6	0.0	7.51	161	0.48	64.3	21.4	333	684	3.65	10.4	14.3
**mg/kg bw/day**	9660	7.8	10.9	85.9	1.8	1.1	0.1	7.92	159	0.47	58.8	20.1	341	948	3.00	10.1	11.4
Olive Oil Infuse with	9666	5.6	12.3	84.9	1.6	0.5	0.1	8.21	160	0.47	57.0	19.5	343	1067	2.33	10.3	11.1
Carbon C60	9672	5.7	10.9	85.5	2.2	0.9	0.0	7.44	150	0.45	60.4	20.2	334	1022	3.69	10.2	11.0
	9679	6.4	14.8	82.7	1.7	0.4	0.1	7.85	166	0.49	62.2	21.2	341	1103	3.32	10.4	11.8

APPENDIX 4.2

Individual data of hematology and blood coagulation

Female

		WBC [x10^9/L]	NEU [%]	LYM [%]	MONO [%]	EOS [%]	BASO [%]	RBC [x10^12/L]	HGB [g/L]	HCT [L/L]	MCV [fL]	MCH [pg]	MCHC [g/L]	PLT [x10^9/L]	RET [%]	PT [sec]	APTT [sec]
**Control**	9681	6.3	14.7	82.8	1.5	0.7	0.0	8.31	160	0.47	56.3	19.2	341	916	2.29	9.8	10.2
EVO Olive Oil	9691	8.9	13.4	82.0	3.0	0.8	0.1	7.72	145	0.42	54.7	18.8	343	1142	2.45	10.0	10.5
	9697	6.5	14.5	82.4	1.8	0.8	0.1	7.72	158	0.46	59.3	20.4	344	1079	2.71	9.9	10.4
	9698	6.2	9.2	86.7	3.0	0.7	0.0	8.05	160	0.46	57.4	19.8	346	948	2.48	10.4	11.3
	9701	5.1	17.7	78.3	2.3	1.5	0.0	7.96	156	0.46	57.3	19.6	341	1305	2.01	9.9	10.4
**4 (3.8)**	9684	6.9	9.1	87.7	1.9	0.6	0.1	7.83	147	0.44	56.6	18.7	331	1093	2.39	10.1	10.0
**mg/kg bw/day**	9689	5.8	17.9	79.5	1.6	0.7	0.1	8.07	158	0.46	57.1	19.5	342	865	2.96	10.1	11.0
Olive Oil Infuse with	9690	5.6	14.1	81.9	2.8	0.8	0.0	8.29	159	0.46	55.8	19.2	344	986	2.10	10.0	11.1
Carbon C60	9692	4.5	13.3	80.4	4.5	1.3	0.1	7.71	151	0.43	56.2	19.6	349	802	2.46	10.0	11.3
	9695	5.4	6.2	91.8	0.9	0.6	0.0	7.95	158	0.46	57.6	19.8	344	1022	2.96	10.3	10.6

Remark: - = No data (coagulated sample)

Study no. 842-400-5742

APPENDIX 5.1

Summary of clinical chemistry
Male

		ALT [U/L]	AST [U/L]	GGT [U/L]	ALP [U/L]	TBIL [µmol/L]	CREA [µmol/L]	UREA [mmol/L]	GLUC [mmol/L]	CHOL [mmol/L]	Pi [mmol/L]	Ca^{++} [mmol/L]	Na^{+} [mmol/L]	K^{+} [mmol/L]	Cl^{-} [mmol/L]	ALB [g/L]	TPROT [g/L]	A/G
**Control**	Mean	65.6	116.4	-	305.2	1.82	35.6	7.40	5.98	2.26	3.10	2.79	141.20	4.55	98.82	43.96	61.36	2.54
EVO Olive Oil	SD	14.3	21.4	-	109.6	0.29	3.3	0.85	0.34	0.38	0.15	0.05	0.94	0.27	1.42	1.22	2.18	0.21
	n	5	5	-	5	5	5	5	5	5	5	5	5	5	5	5	5	5
**4 (3.8)**	Mean	56.2	95.4	-	299.0	2.20	36.0	8.70	5.34	2.37	3.17	2.84	143.96	4.36	100.64	45.02	62.34	2.60
**mg/kg bw/day**	SD	10.0	12.1	-	68.9	0.56	1.9	0.86	0.24	0.40	0.12	0.05	1.63	0.12	2.06	1.66	1.52	0.19
Olive Oil Infuse with	n	5	5	-	5	5	5	5	5	5	5	5	5	5	5	5	5	5
Carbon C60	±%	-14	-18	-	-2	21	1	18	-11	5	2	2	2	-4	2	2	2	2
									**									
		NS	NS	-	NS	NS	NS	NS	DN	NS	NS	NS	NS	NS	NS	NS	NS	NS

REMARKS: ±% = Percent Deviation Versus Control
NS = Not Significant
* = $p < 0.05$
** = $p < 0.01$
U = Mann-Whitney U - test Versus Control
DN = Duncan's multiple range test
- = No data (Values were below the quantification limit - 3 U/L)

APPENDIX 5.1

Summary of clinical chemistry
Female

		ALT [U/L]	AST [U/L]	GGT [U/L]	ALP [U/L]	TBIL [µmol/L]	CREA [µmol/L]	UREA [mmol/L]	GLUC [mmol/L]	CHOL [mmol/L]	Pi [mmol/L]	Ca^{++} [mmol/L]	Na^{+} [mmol/L]	K^{+} [mmol/L]	Cl^{-} [mmol/L]	ALB [g/L]	TPROT [g/L]	A/G
**Control**	Mean	48.2	117.6	-	190.4	2.04	38.4	6.22	4.98	1.74	2.57	2.63	141.14	4.08	102.18	45.26	60.80	2.90
EVO Olive Oil	SD	17.2	35.9	-	36.1	0.17	2.1	0.97	0.49	0.17	0.12	0.05	0.91	0.29	0.97	0.93	1.70	0.21
	n	5	5	-	5	5	5	5	5	5	5	5	5	5	5	5	5	5
**4 (3.8)**	Mean	38.80	90.80	-	155.6	2.00	37.6	5.58	5.03	1.82	2.50	2.61	140.94	3.84	101.64	44.60	60.24	2.86
**mg/kg bw/day**	SD	5.17	19.69	-	32.3	0.37	1.9	0.51	0.30	0.21	0.17	0.06	0.75	0.21	0.98	0.97	1.69	0.32
Olive Oil Infuse with	n	5	5	-	5	5	5	5	5	5	5	5	5	5	5	5	5	5
Carbon C60	±%	-20	-23	-	-18	-2	-2	-10	1	5	-3	-1	0	-6	-1	-1	-1	-1
		NS	NS	-	NS	NS	NS	NS	NS	NS	NS	DN	NS	NS	NS	NS	NS	NS

REMARKS: ±% = Percent Deviation Versus Control
NS = Not Significant
* = p < 0.05
** = p < 0.01
U = Mann-Whitney U - test Versus Control
DN = Duncan's multiple range test
- = No data (Values were below the quantification limit - 3 U/L)

APPENDIX 5.2

Individual data of clinical chemistry

Male

Group	Animal number	ALT [U/L]	AST [U/L]	GGT [U/L]	ALP [U/L]	TBIL [µmol/L]	CREA [µmol/L]	UREA [mmol/L	GLUC [mmol/L	CHOL [mmol/L	Pi [mmol/L	Ca^{++} [mmol/L	Na^{+} [mmol/L	K^{+} [mmol/L	Cl^{-} [mmol/L	ALB [g/L]	TPROT [g/L]	A/G
**Control**	9656	66	100	bql	483	1.4	38	7.6	6.01	2.83	2.99	2.81	142.0	4.93	99.7	45.0	61.6	2.7
EVO Olive Oil	9657	83	151	bql	287	2.1	36	6.3	6.19	2.35	2.99	2.72	140.0	4.38	99.8	44.0	60.9	2.6
	9658	44	106	bql	213	1.7	38	8.4	5.77	2.27	3.20	2.76	140.8	4.30	96.5	41.9	58.5	2.5
	9662	72	123	bql	220	2.1	30	6.8	6.41	1.84	3.00	2.81	142.3	4.40	98.4	44.7	61.2	2.7
	9673	63	102	bql	323	1.8	36	7.9	5.54	2.00	3.31	2.85	140.9	4.76	99.7	44.2	64.6	2.2
**4 (3.8)**	9655	46	89	bql	357	1.6	34	9.1	5.68	2.29	3.14	2.84	145.3	4.50	101.4	43.8	62.6	2.3
**mg/kg bw/day**	9660	57	93	bql	279	2.1	39	9.4	5.04	1.87	3.31	2.81	142.9	4.33	101.5	43.5	60.4	2.6
Olive Oil Infuse with	9666	71	94	bql	376	2.5	36	9.4	5.30	2.46	3.01	2.79	145.0	4.19	98.1	47.1	64.4	2.7
Carbon C60	9672	48	116	bql	205	1.8	35	7.5	5.46	2.97	3.25	2.88	145.0	4.33	103.2	44.2	61.4	2.6
	9679	59	85	bql	278	3.0	36	8.1	5.23	2.26	3.13	2.90	141.6	4.45	99.0	46.5	62.9	2.8

Remark: GGT values were below the quantification limit (3 U/L)

APPENDIX 5.2

Individual data of clinical chemistry

Female

Group	Animal number	ALT [U/L]	AST [U/L]	GGT [U/L]	ALP [U/L]	TBIL [µmol/L]	CREA [µmol/L]	UREA [mmol/L	GLUC [mmol/L	CHOL [mmol/L	Pi [mmol/L	Ca^{++} [mmol/L	Na^{+} [mmol/L	K^{+} [mmol/L	Cl^{-} [mmol/L	ALB [g/L]	TPROT [g/L]	A/G
**Control**	9681	44	104	bql	173	2.0	38	6.8	4.93	1.70	2.58	2.64	142.4	3.82	102.1	45.9	62.6	2.7
EVO Olive Oil	9691	74	96	bql	169	2.0	40	7.3	4.57	1.89	2.77	2.67	140.3	4.40	100.8	43.7	58.1	3.0
	9697	32	97	bql	230	1.8	36	5.2	5.83	1.78	2.54	2.59	141.0	3.96	102.7	45.9	60.4	3.2
	9698	35	110	bql	228	2.2	37	5.2	4.78	1.46	2.50	2.68	140.3	4.38	101.9	45.1	61.6	2.7
	9701	56	181	bql	152	2.2	41	6.6	4.81	1.86	2.46	2.58	141.7	3.82	103.4	45.7	61.3	2.9
**4 (3.8)**	9684	40	72	bql	140	2.4	40	5.6	5.16	2.12	2.74	2.67	141.6	4.14	102.7	44.2	59.8	2.8
**mg/kg bw/day**	9689	38	80	bql	183	1.4	37	5.8	5.15	1.76	2.52	2.58	140.5	3.63	101.2	44.2	62.5	2.4
Olive Oil Infuse with	9690	47	123	bql	152	2.1	39	4.7	4.86	1.91	2.38	2.67	140.0	3.88	100.9	45.5	59.2	3.3
Carbon C60	9692	34	94	bql	191	2.0	37	5.9	5.38	1.75	2.29	2.52	140.8	3.66	102.7	43.4	58.3	2.9
	9695	35	85	bql	112	2.1	35	5.9	4.62	1.55	2.56	2.61	141.8	3.88	100.7	45.7	61.4	2.9

Remark: GGT values were below the quantification limit (3 U/L)

APPENDIX 6.1

Summary of necropsy findings

Male

**Organs**	**Observations**	**Control**<br>EVO Olive Oil	**4 (3.8) mg/kg bw/day**<br>Olive Oil Infuse with Carbon C60
	No macroscopic findings	4/5	5/5
Thymus	Hemorrhages	1/5	0/5
Lungs	Reddish-brown spot on the left side small lobe - pea-sized	0/5	0/5
Skin	Scars - left ear, neck	0/5	0/5

Remark: Frequency of observations: number of animals with observation/number of animals examined

APPENDIX 6.2

Summary of necropsy findings
Female

Organs	Observations	Control<br>EVO Olive Oil	4 (3.8) mg/kg bw/day<br>Olive Oil Infuse with Carbon C60
	No macroscopic findings	3/5	2/5
Thymus	Point-like hemorrhages	0/5	1/5
Lungs	Hemorrhage	0/5	1/5
Kidneys	Pyelectasia	1/5	0/5
Uterus	Hydrometra	1/5	1/5
	Cyst	0/5	0/5

Remark: Frequency of observations: number of animals with observation/number of animals examined

APPENDIX 6.2

Individual necropsy findings

Male

Group	Animal number	Organs	Observations	
**Control**	9656		No macroscopic findings	Day 14
EVO Olive Oil	9657		No macroscopic findings	Day 14
	9658	Thymus	Hemorrhages	Day 14
	9662		No macroscopic findings	Day 14
	9673		No macroscopic findings	Day 14
**4 (3.8)**	9655		No macroscopic findings	Day 14
**mg/kg bw/day**	9660		No macroscopic findings	Day 14
Olive Oil Infuse with	9666		No macroscopic findings	Day 14
Carbon C60	9672		No macroscopic findings	Day 14
	9679		No macroscopic findings	Day 14

APPENDIX 6.2

Individual necropsy findings
Female

Group	Animal number	Organs	Observations	
**Control**	9681	Uterus	Hydrometra - moderate	Day 14
EVO Olive Oil	9691		No macroscopic findings	Day 14
	9697		No macroscopic findings	Day 14
	9698		No macroscopic findings	Day 14
	9701	Kidneys	Pyelectasia - right side	Day 14
**4 (3.8)**	9684	Thymus	Point-like hemorrhages	Day 14
**mg/kg bw/day**	9689	Lungs	Hemorrhage - left side small lobe	Day 14
Olive Oil Infuse with	9690		No macroscopic findings	Day 14
Carbon C60	9692	Uterus	Hydrometra - moderate	Day 14
	9695		No macroscopic findings	Day 14

APPENDIX 7.1
Summary of organ weight
Male

		Body weight	Brain	Liver	Kidneys	Heart	Thymus	Spleen	Testes	Epididy-mides	Seminal vesicles† Prostate	Adrenal glands
						Organ weight (g)						
**Control**	Mean	247.0	1.89	7.98	1.96	0.73	0.60	0.56	2.79	0.75	1.04	0.064
EVO Olive Oil	SD	6.67	0.05	0.69	0.13	0.04	0.09	0.10	0.13	0.09	0.20	0.008
	n	5	5	5	5	5	5	5	5	5	5	5
**4 (3.8)**	Mean	241.0	1.93	7.62	1.84	0.71	0.65	0.60	2.80	0.80	0.94	0.066
**mg/kg bw/day**	SD	9.80	0.11	0.58	0.14	0.06	0.10	0.09	0.09	0.08	0.07	0.012
Olive Oil Infuse with	n	5	5	5	5	5	5	5	5	5	5	5
Carbon C60	± %	-2	2	-4	-6	-3	9	8	0	7	-9	3
		NS	NS	NS	NS	NS	NS	NS	NS	NS	NS	NS

Remarks:
† = Seminal vesicles with coagulating gland
±% = Percent Deviation Versus Control
NS = Not Significant
* = $p < 0.05$
** = $p < 0.01$
U = Mann-Whitney U - test Versus Control
DN = Duncan's multiple range test

APPENDIX 7.1
Summary of organ weight
Male

		Organ weight relative to body weight (%)									
		**Brain**	**Liver**	**Kidneys**	**Heart**	**Thymus**	**Spleen**	**Testes**	**Epididy-mides**	**Seminal vesicles† Prostate**	**Adrenal glands**
**Control** EVO Olive Oil	Mean	0.764	3.225	0.794	0.296	0.242	0.225	1.130	0.303	0.421	0.0261
	SD	0.023	0.201	0.042	0.010	0.037	0.039	0.032	0.042	0.077	0.0036
	n	5	5	5	5	5	5	5	5	5	5
**4 (3.8) mg/kg bw/day** Olive Oil Infuse with Carbon C60	Mean	0.802	3.160	0.765	0.294	0.268	0.249	1.164	0.333	0.393	0.0277
	SD	0.054	0.125	0.042	0.020	0.036	0.029	0.046	0.029	0.041	0.0056
	n	5	5	5	5	5	5	5	5	5	5
	± %	5	-2	-4	-1	11	11	3	10	-7	6
		NS	NS	NS	NS	NS	NS	NS	NS	NS	NS

Remarks:
† = Seminal vesicles with coagulating gland
±% = Percent Deviation Versus Control
NS = Not Significant
* = $p < 0.05$
** = $p < 0.01$
U = Mann-Whitney U - test Versus Control
DN = Duncan's multiple range test

APPENDIX 7.1
Summary of organ weight
Male

		Organ weight and body weight relative to brain weight (%)									
		Body weight	Liver	Kidneys	Heart	Thymus	Spleen	Testes	Epididy-mides	Seminal vesicles† Prostate	Adrenal glands
**Control**	Mean	13101.7	422.97	104.10	38.81	31.60	29.55	148.11	39.79	55.32	3.42
EVO Olive Oil	SD	412.86	36.53	7.86	1.67	4.49	5.64	7.43	5.91	11.69	0.44
	n	5	5	5	5	5	5	5	5	5	5
**4 (3.8)**	Mean	12521.5	395.96	95.91	36.96	33.75	31.21	145.62	41.85	49.05	3.45
**mg/kg bw/day**	SD	902.84	35.45	10.64	4.83	6.53	4.22	10.44	6.82	4.35	0.61
Olive Oil Infuse with	n	5	5	5	5	5	5	5	5	5	5
Carbon C60	± %	-4	-6	-8	-5	7	6	-2	5	-11	1
		NS	NS	NS	NS	NS	NS	NS	NS	NS	NS

Remarks: † = Seminal vesicles with coagulating gland
±% = Percent Deviation Versus Control
NS = Not Significant
* = p < 0.05
** = p < 0.01
U = Mann-Whitney U - test Versus Control
DN = Duncan's multiple range test

APPENDIX 7.1
Summary of organ weight
Female

		Body weight	Organ weight (g) Brain	Liver	Kidneys	Heart	Thymus	Spleen	Uterus	Ovaries	Adrenal glands
**Control**	Mean	161.2	1.73	5.06	1.29	0.53	0.51	0.33	0.50	0.080	0.073
EVO Olive Oil	SD	3.70	0.03	0.24	0.08	0.02	0.05	0.02	0.13	0.008	0.006
	n	5	5	5	5	5	5	5	5	5	5
**4 (3.8)**	Mean	164.0	1.71	5.04	1.31	0.52	0.47	0.37	0.45	0.085	0.068
**mg/kg bw/day**	SD	7.31	0.07	0.14	0.04	0.06	0.05	0.05	0.09	0.017	0.007
Olive Oil Infuse with	n	5	5	5	5	5	5	5	5	5	5
Carbon C60	± %	2	-1	0	2	-1	-8	10	-10	6	-7
		NS	NS	NS	NS	NS	NS	NS	NS	NS	NS

REMARKS: ±% = Percent Deviation Versus Control
NS = Not Significant
* = p < 0.05
** = p < 0.01
U = Mann-Whitney U - test Versus Control
DN = Duncan's multiple range test

APPENDIX 7.1
Summary of organ weight
Female

		Organ weight relative to body weight (%) Brain	Liver	Kidneys	Heart	Thymus	Spleen	Uterus	Ovaries	Adrenal glands
**Control**	Mean	1.075	3.141	0.799	0.326	0.314	0.206	0.314	0.0498	0.0455
EVO Olive Oil	SD	0.021	0.142	0.039	0.014	0.027	0.009	0.087	0.0040	0.0033
	n	5	5	5	5	5	5	5	5	5
**4 (3.8)**	Mean	1.044	3.079	0.801	0.318	0.285	0.223	0.278	0.0518	0.0415
**mg/kg bw/day**	SD	0.046	0.109	0.023	0.028	0.022	0.029	0.065	0.0086	0.0051
Olive Oil Infuse with	n	5	5	5	5	5	5	5	5	5
Carbon C60	± %	-3	-2	0	-3	-9	8	-12	4	-9
		NS	NS	NS	NS	NS	NS	NS	NS	NS

REMARKS: ±% = Percent Deviation Versus Control
NS = Not Significant
* = p < 0.05
** = p < 0.01
U = Mann-Whitney U - test Versus Control
DN = Duncan's multiple range test

APPENDIX 7.1
Summary of organ weight
Female

		Organ weight and body weight relative to brain weight (%)								
		**Body weight**	**Liver**	**Kidneys**	**Heart**	**Thymus**	**Spleen**	**Uterus**	**Ovaries**	**Adrenal glands**
**Control**	Mean	9307.9	292.19	74.36	30.37	29.23	19.17	29.12	4.64	4.24
EVO Olive Oil	SD	183.81	10.99	4.24	1.15	2.81	1.12	7.55	0.43	0.30
	n	5	5	5	5	5	5	5	5	5
**4 (3.8)**	Mean	9596.6	295.42	76.77	30.53	27.38	21.43	26.58	4.98	3.98
**mg/kg bw/day**	SD	434.47	16.29	2.09	3.03	2.93	3.09	5.93	0.97	0.39
Olive Oil Infuse with	n	5	5	5	5	5	5	5	5	5
Carbon C60	± %	3	1	3	1	-6	12	-9	7	-6
		NS	NS	NS	NS	NS	NS	NS	NS	NS

REMARKS: ±% = Percent Deviation Versus Control
NS = Not Significant
* = $p < 0.05$
** = $p < 0.01$
U = Mann-Whitney U - test Versus Control
DN = Duncan's multiple range test

APPENDIX 7.2
Individual organ weight
Male

Group	Animal number	Body weight	Organ weight (g) Brain	Liver	Kidneys	Heart	Thymus	Spleen	Testes	Epididy-mides	Seminal vesicles† Prostate	Adrenal glands
**Control**	9656	249	1.92	8.06	2.10	0.77	0.71	0.56	2.69	0.66	1.11	0.054
EVO Olive Oil	9657	237	1.82	6.94	1.86	0.67	0.58	0.53	2.66	0.88	1.03	0.072
	9658	251	1.93	8.03	1.91	0.75	0.48	0.41	2.92	0.70	0.83	0.072
	9662	254	1.84	8.87	2.11	0.75	0.56	0.68	2.93	0.80	1.34	0.060
	9673	244	1.92	7.98	1.83	0.72	0.65	0.60	2.76	0.70	0.89	0.064
**4 (3.8)**	9655	234	1.95	7.51	1.86	0.63	0.61	0.64	2.84	0.74	0.95	0.086
**mg/kg bw/day**	9660	246	1.75	7.78	1.95	0.79	0.77	0.59	2.81	0.94	0.90	0.060
Olive Oil Infuse with	9666	234	1.99	6.99	1.62	0.72	0.65	0.50	2.64	0.74	0.99	0.060
Carbon C60	9672	256	2.05	8.52	1.98	0.74	0.71	0.73	2.87	0.80	0.86	0.058
	9679	235	1.91	7.32	1.81	0.67	0.50	0.55	2.85	0.79	1.02	0.068

Remark: † = Seminal vesicles with coagulating gland

APPENDIX 7.2
Individual organ weight
Male

Group	Animal number	Brain	Liver	Kidneys	Heart	Thymus	Spleen	Testes	Epididy-mides	Seminal vesicles† Prostate	Adrenal glands
		Organ weight relative to body weight (%)									
**Control**	9656	0.771	3.237	0.843	0.309	0.285	0.225	1.080	0.265	0.446	0.0217
EVO Olive Oil	9657	0.768	2.928	0.785	0.283	0.245	0.224	1.122	0.371	0.435	0.0304
	9658	0.769	3.199	0.761	0.299	0.191	0.163	1.163	0.279	0.331	0.0287
	9662	0.724	3.492	0.831	0.295	0.220	0.268	1.154	0.315	0.528	0.0236
	9673	0.787	3.270	0.750	0.295	0.266	0.246	1.131	0.287	0.365	0.0262
**4 (3.8)**	9655	0.833	3.209	0.795	0.269	0.261	0.274	1.214	0.316	0.406	0.0368
**mg/kg bw/day**	9660	0.711	3.163	0.793	0.321	0.313	0.240	1.142	0.382	0.366	0.0244
Olive Oil Infuse with	9666	0.850	2.987	0.692	0.308	0.278	0.214	1.128	0.316	0.423	0.0256
Carbon C60	9672	0.801	3.328	0.773	0.289	0.277	0.285	1.121	0.313	0.336	0.0227
	9679	0.813	3.115	0.770	0.285	0.213	0.234	1.213	0.336	0.434	0.0289

Remark: † = Seminal vesicles with coagulating gland

APPENDIX 7.2
Individual organ weight
Male

Group	Animal number	Organ weight and body weight relative to brain weight (%) Body weight	Liver	Kidneys	Heart	Thymus	Spleen	Testes	Epididy-mides	Seminal vesicles† Prostate	Adrenal glands
**Control**	9656	12968.8	419.79	109.38	40.10	36.98	29.17	140.10	34.38	57.81	2.81
EVO Olive Oil	9657	13022.0	381.32	102.20	36.81	31.87	29.12	146.15	48.35	56.59	3.96
	9658	13005.2	416.06	98.96	38.86	24.87	21.24	151.30	36.27	43.01	3.73
	9662	13804.3	482.07	114.67	40.76	30.43	36.96	159.24	43.48	72.83	3.26
	9673	12708.3	415.63	95.31	37.50	33.85	31.25	143.75	36.46	46.35	3.33
**4 (3.8)**	9655	12000.0	385.13	95.38	32.31	31.28	32.82	145.64	37.95	48.72	4.41
**mg/kg bw/day**	9660	14057.1	444.57	111.43	45.14	44.00	33.71	160.57	53.71	51.43	3.43
Olive Oil Infuse with	9666	11758.8	351.26	81.41	36.18	32.66	25.13	132.66	37.19	49.75	3.02
Carbon C60	9672	12487.8	415.61	96.59	36.10	34.63	35.61	140.00	39.02	41.95	2.83
	9679	12303.7	383.25	94.76	35.08	26.18	28.80	149.21	41.36	53.40	3.56

Remark: † = Seminal vesicles with coagulating gland

APPENDIX 7.2
Individual organ weight
Female

Group	Animal number	Body weight	Organ weight (g) Brain	Liver	Kidneys	Heart	Thymus	Spleen	Uterus	Ovaries	Adrenal glands
**Control**	9681	157	1.74	5.07	1.28	0.54	0.46	0.32	0.68	0.076	0.069
EVO Olive Oil	9691	162	1.73	5.34	1.28	0.50	0.58	0.33	0.42	0.082	0.073
	9697	166	1.78	5.26	1.33	0.55	0.49	0.34	0.44	0.084	0.082
	9698	163	1.71	4.78	1.38	0.54	0.52	0.36	0.38	0.090	0.067
	9701	158	1.70	4.86	1.17	0.50	0.48	0.31	0.60	0.070	0.076
**4 (3.8)**	9684	171	1.74	5.08	1.32	0.61	0.49	0.41	0.42	0.114	0.065
**mg/kg bw/day**	9689	154	1.63	4.94	1.25	0.51	0.43	0.31	0.54	0.074	0.060
Olive Oil Infuse with	9690	170	1.66	5.27	1.33	0.50	0.52	0.39	0.43	0.087	0.066
Carbon C60	9692	159	1.72	5.01	1.32	0.46	0.40	0.41	0.54	0.077	0.080
	9695	166	1.80	4.92	1.34	0.53	0.50	0.31	0.33	0.074	0.069

APPENDIX 7.2
Individual organ weight
Female

Group	Animal number	Organ weight relative to body weight (%) Brain	Liver	Kidneys	Heart	Thymus	Spleen	Uterus	Ovaries	Adrenal glands
**Control**	9681	1.108	3.229	0.815	0.344	0.293	0.204	0.433	0.0484	0.0439
EVO Olive Oil	9691	1.068	3.296	0.790	0.309	0.358	0.204	0.259	0.0506	0.0451
	9697	1.072	3.169	0.801	0.331	0.295	0.205	0.265	0.0506	0.0494
	9698	1.049	2.933	0.847	0.331	0.319	0.221	0.233	0.0552	0.0411
	9701	1.076	3.076	0.741	0.316	0.304	0.196	0.380	0.0443	0.0481
**4 (3.8)**	9684	1.018	2.971	0.772	0.357	0.287	0.240	0.246	0.0667	0.0380
**mg/kg bw/day**	9689	1.058	3.208	0.812	0.331	0.279	0.201	0.351	0.0481	0.0390
Olive Oil Infuse with	9690	0.976	3.100	0.782	0.294	0.306	0.229	0.253	0.0512	0.0388
Carbon C60	9692	1.082	3.151	0.830	0.289	0.252	0.258	0.340	0.0484	0.0503
	9695	1.084	2.964	0.807	0.319	0.301	0.187	0.199	0.0446	0.0416

APPENDIX 7.2

Individual organ weight

Female

Group	Animal number	Body weight	Liver	Kidneys	Heart	Thymus	Spleen	Uterus	Ovaries	Adrenal glands
		Organ weight and body weight relative to brain weight (%)								
**Control**	9681	9023.0	291.38	73.56	31.03	26.44	18.39	39.08	4.37	3.97
EVO Olive Oil	9691	9364.2	308.67	73.99	28.90	33.53	19.08	24.28	4.74	4.22
	9697	9325.8	295.51	74.72	30.90	27.53	19.10	24.72	4.72	4.61
	9698	9532.2	279.53	80.70	31.58	30.41	21.05	22.22	5.26	3.92
	9701	9294.1	285.88	68.82	29.41	28.24	18.24	35.29	4.12	4.47
**4 (3.8)**	9684	9827.6	291.95	75.86	35.06	28.16	23.56	24.14	6.55	3.74
**mg/kg bw/day**	9689	9447.9	303.07	76.69	31.29	26.38	19.02	33.13	4.54	3.68
Olive Oil Infuse with	9690	10241.0	317.47	80.12	30.12	31.33	23.49	25.90	5.24	3.98
Carbon C60	9692	9244.2	291.28	76.74	26.74	23.26	23.84	31.40	4.48	4.65
	9695	9222.2	273.33	74.44	29.44	27.78	17.22	18.33	4.11	3.83

APPENDIX 8.1
Summary of histopathology findings
Male

Organs	Observations	Control<br>EVO Olive Oil	Incidence of observations<br>4 (3.8) mg/kg bw/day<br>Olive Oil Infuse with Carbon C60
Adrenal glands	No lesion	5/5	5/5
Aorta	No lesion	5/5	5/5
Bone marrow	No lesion	5/5	5/5
Brain	No lesion	5/5	5/5
Cecum	No lesion	5/5	5/5
Colon	No lesion	5/5	5/5
Duodenum	No lesion	5/5	5/5
Eyes + optic nerve	No lesion	5/5	5/5
Epididymides	No lesion	5/5	5/5
Esophagus	No lesion	5/5	5/5
Harderian glands	No lesion	5/5	5/5
Heart	No lesion	5/5	5/5
Ileum	No lesion	5/5	5/5
Jejunum	No lesion	5/5	5/5
Kidneys	No lesion	5/5	5/5
Lachrymal glands	No lesion	5/5	5/5
Liver	No lesion	5/5	5/5
**Lungs**	**Focal inflammation**	**0/5**	**0/5**
	**Hemorrhage**	**0/5**	**0/5**
Mammary gland	No lesion	5/5	5/5
Mesenteric lymph nodes	No lesion	5/5	5/5
Muscle (quadriceps)	No lesion	5/5	5/5
Nasal cavity	No lesion	5/5	5/5
Pancreas	No lesion	5/5	5/5
Pituitary	No lesion	5/5	5/5
Prostate	No lesion	5/5	5/5
Rectum	No lesion	5/5	5/5
Salivary glands (subm.)	No lesion	5/5	5/5
Sciatic nerve	No lesion	5/5	5/5
Seminal vesicle †	No lesion	5/5	5/5
**Skin - Ear**	**Subacute dermatitis**	**0/5**	**0/5**
Spinal cord	No lesion	5/5	5/5
Spleen	No lesion	5/5	5/5
Sternum	No lesion	5/5	5/5
Stomach	No lesion	5/5	5/5
Subm. lymph nodes	No lesion	5/5	5/5
**Thymus**	**Acute hemorrhage**	**1/5**	**0/5**
Thyroid + parathyroid	No lesion	5/5	5/5
Testes	No lesion	5/5	5/5
Trachea	No lesion	5/5	5/5
Urinary bladder	No lesion	5/5	5/5

Remark: Frequency of observations: number of animals with observation/number of animals examined
† = Seminal vesicle with coagulating gland
subm. = Submandibular

APPENDIX 8.2
Summary of histopathology findings
Female

Organs	Observations	Control<br>EVO Olive Oil	Incidence of observations<br>4 (3.8) mg/kg bw/day<br>Olive Oil Infuse with Carbon C60
Adrenal glands	No lesion	5/5	5/5
Aorta	No lesion	5/5	5/5
Bone marrow	No lesion	5/5	5/5
Brain	No lesion	5/5	5/5
Cecum	No lesion	5/5	5/5
Colon	No lesion	5/5	5/5
Duodenum	No lesion	5/5	5/5
Eyes + optic nerve	No lesion	5/5	5/5
Esophagus	No lesion	5/5	5/5
Harderian glands	No lesion	5/5	5/5
Heart	No lesion	5/5	5/5
Ileum	No lesion	5/5	5/5
Jejunum	No lesion	5/5	5/5
**Kidneys**	**Pyelectasia**	**1/5**	**0/5**
Lachrymal glands	No lesion	5/5	5/5
Liver	No lesion	5/5	5/5
**Lungs**	**Alveolar emphysema**	**0/5**	**1/5**
	**Hemorrhage**	**0/5**	**1/5**
Mammary gland	No lesion	5/5	5/5
Mesenteric lymph nodes	No lesion	5/5	5/5
Muscle (quadriceps)	No lesion	5/5	5/5
Nasal cavity	No lesion	5/5	5/5
Ovaries	No lesion	5/5	5/5
Pancreas	No lesion	5/5	5/5
Pituitary	No lesion	5/5	5/5
Rectum	No lesion	5/5	5/5
Salivary glands (subm)	No lesion	5/5	5/5
Sciatic nerve	No lesion	5/5	5/5
Skin	No lesion	5/5	5/5
Spinal cord	No lesion	5/5	5/5
Spleen	No lesion	5/5	5/5
Sternum	No lesion	5/5	5/5
Stomach	No lesion	5/5	5/5
Subm. lymph nodes	No lesion	5/5	5/5
**Thymus**	**Acute hemorrhage**	**0/5**	**1/5**
Thyroid + parathyroid:	No lesion	5/5	5/5
Trachea	No lesion	5/5	5/5
Urinary bladder	No lesion	5/5	5/5
**Uterus**	**Dilatation**	**2/5**	**1/5**
	**Cyst**	**0/5**	**0/5**
Vagina	No lesion	5/5	5/5

Remark: Frequency of observations: number of animals with observation/number of animals examined
subm. = Submandibular

APPENDIX 8.2

Individual histopathology findings
Male

**Group 1: Control EVO Olive Oil**		**Animal numbers**				
**Organs**	**Observations**	**9656**	**9657**	**9658**	**9662**	**9673**
Adrenal glands	No lesion	+	+	+	+	+
Aorta	No lesion	+	+	+	+	+
Bone marrow	No lesion	+	+	+	+	+
Brain	No lesion	+	+	+	+	+
Cecum	No lesion	+	+	+	+	+
Colon	No lesion	+	+	+	+	+
Duodenum	No lesion	+	+	+	+	+
Eyes + optic nerve	No lesion	+	+	+	+	+
Epididymides	No lesion	+	+	+	+	+
Esophagus	No lesion	+	+	+	+	+
Harderian glands	No lesion	+	+	+	+	+
Heart	No lesion	+	+	+	+	+
Ileum	No lesion	+	+	+	+	+
Jejunum	No lesion	+	+	+	+	+
Kidneys	No lesion	+	+	+	+	+
Lachrymal glands	No lesion	+	+	+	+	+
Liver	No lesion	+	+	+	+	+
Lungs	No lesion	+	+	+	+	+
Mammary gland	No lesion	+	+	+	+	+
Mesenteric lymph nodes	No lesion	+	+	+	+	+
Muscle (quadriceps)	No lesion	+	+	+	+	+
Nasal cavity	No lesion	+	+	+	+	+
Pancreas	No lesion	+	+	+	+	+
Pituitary	No lesion	+	+	+	+	+
Prostate	No lesion	+	+	+	+	+
Rectum	No lesion	+	+	+	+	+
Salivary glands (subm.)	No lesion	+	+	+	+	+
Sciatic nerve	No lesion	+	+	+	+	+
Seminal vesicle †	No lesion	+	+	+	+	+
Skin	No lesion	+	+	+	+	+
Spinal cord	No lesion	+	+	+	+	+
Spleen	No lesion	+	+	+	+	+
Sternum	No lesion	+	+	+	+	+
Stomach	No lesion	+	+	+	+	+
Subm. lymph nodes	No lesion	+	+	+	+	+
Thymus	Acute hemorrhage	-	-	**2**	-	-
Thyroid + parathyroid	No lesion	+	+	+	+	+
Testes	No lesion	+	+	+	+	+
Trachea	No lesion	+	+	+	+	+
Urinary bladder	No lesion	+	+	+	+	+

Remarks:

+ = Observation present
- = Observation not present
/ = No data
† = Seminal vesicle with coagulating gland
subm. = Submandibular
b = both sides
o = one side

1 = Minimal
2 = Mild
3 = Moderate
4 = Severe (Marked)

APPENDIX 8.2

Individual histopathology findings
Male

Group: 3 (3.8) mg/kg bw/day<br>Olive Oil Infuse with Carbon C60		Animal numbers				
**Organs**	**Observations**	**9655**	**9660**	**9666**	**9672**	**9679**
Adrenal glands	No lesion	+	+	+	+	+
Aorta	No lesion	+	+	+	+	+
Bone marrow	No lesion	+	+	+	+	+
Brain	No lesion	+	+	+	+	+
Cecum	No lesion	+	+	+	+	+
Colon	No lesion	+	+	+	+	+
Duodenum	No lesion	+	+	+	+	+
Eyes + optic nerve	No lesion	+	+	+	+	+
Epididymides	No lesion	+	+	+	+	+
Esophagus	No lesion	+	+	+	+	+
Harderian glands	No lesion	+	+	+	+	+
Heart	No lesion	+	+	+	+	+
Ileum	No lesion	+	+	+	+	+
Jejunum	No lesion	+	+	+	+	+
Kidneys	No lesion	+	+	+	+	+
Lachrymal glands	No lesion	+	+	+	+	+
Liver	No lesion	+	+	+	+	+
Lungs	No lesion	+	+	+	+	+
Mammary gland	No lesion	+	+	+	+	+
Mesenteric lymph nodes	No lesion	+	+	+	+	+
Muscle (quadriceps)	No lesion	+	+	+	+	+
Nasal cavity	No lesion	+	+	+	+	+
Pancreas	No lesion	+	+	+	+	+
Pituitary	No lesion	+	+	+	+	+
Prostate	No lesion	+	+	+	+	+
Rectum	No lesion	+	+	+	+	+
Salivary glands (subm.)	No lesion	+	+	+	+	+
Sciatic nerve	No lesion	+	+	+	+	+
Seminal vesicle †	No lesion	+	+	+	+	+
Skin	No lesion	+	+	+	+	+
Spinal cord	No lesion	+	+	+	+	+
Spleen	No lesion	+	+	+	+	+
Sternum	No lesion	+	+	+	+	+
Stomach	No lesion	+	+	+	+	+
Subm. lymph nodes	No lesion	+	+	+	+	+
Thymus	No lesion	+	+	+	+	+
Thyroid + parathyroid	No lesion	+	+	+	+	+
Testes	No lesion	+	+	+	+	+
Trachea	No lesion	+	+	+	+	+
Urinary bladder	No lesion	+	+	+	+	+

Remarks:

+ = Observation present
- = Observation not present
/ = No data
† = Seminal vesicle with coagulating gland
subm. = Submandibular
b = both sides
o = one side

1 = Minimal
2 = Mild
3 = Moderate
4 = Severe (Marked)

APPENDIX 8.2

Individual histopathology findings

Female

**Group 1: Control**<br>**EVO Olive Oil**		**Animal numbers**				
**Organs**	**Observations**	**9681**	**9691**	**9697**	**9698**	**9701**
Adrenal glands	No lesion	+	+	+	+	+
Aorta	No lesion	+	+	+	+	+
Bone marrow	No lesion	+	+	+	+	+
Brain	No lesion	+	+	+	+	+
Cecum	No lesion	+	+	+	+	+
Colon	No lesion	+	+	+	+	+
Duodenum	No lesion	+	+	+	+	+
Eyes + optic nerve	No lesion	+	+	+	+	+
Esophagus	No lesion	+	+	+	+	+
Harderian glands	No lesion	+	+	+	+	+
Heart	No lesion	+	+	+	+	+
Ileum	No lesion	+	+	+	+	+
Jejunum	No lesion	+	+	+	+	+
**Kidneys**	**Pyelectasia**	-	-	-	-	+o
Lachrymal glands	No lesion	+	+	+	+	+
Liver	No lesion	+	+	+	+	+
Lungs	No lesion	+	+	+	+	+
Mammary gland	No lesion	+	+	+	+	+
Mesenteric lymph nodes	No lesion	+	+	+	+	+
Muscle (quadriceps)	No lesion	+	+	+	+	+
Nasal cavity	No lesion	+	+	+	+	+
Ovaries:	No lesion	+	+	+	+	+
Pancreas	No lesion	+	+	+	+	+
Pituitary	No lesion	+	+	+	+	+
Rectum	No lesion	+	+	+	+	+
Salivary glands (subm)	No lesion	+	+	+	+	+
Sciatic nerve	No lesion	+	+	+	+	+
Skin	No lesion	+	+	+	+	+
Spinal cord	No lesion	+	+	+	+	+
Spleen	No lesion	+	+	+	+	+
Sternum	No lesion	+	+	+	+	+
Stomach	No lesion	+	+	+	+	+
Subm. lymph nodes	No lesion	+	+	+	+	+
Thymus	No lesion	+	+	+	+	+
Thyroid + parathyroid	No lesion	+	+	+	+	+
Trachea	No lesion	+	+	+	+	+
Urinary bladder	No lesion	+	+	+	+	+
**Uterus**	**Dilatation**	+	-	-	-	+
Vagina	No lesion	+	+	+	+	+

Remark:
+ = Observation present
- = Observation not present
/ = No data
subm. = Submandibular
b = both sides
o = one side

1 = Minimal
2 = Mild
3 = Moderate
4 = Severe (Marked)

APPENDIX 8.2

Individual histopathology findings

Female

**Group:3 (3.8) mg/kg bw/day Olive Oil Infuse with Carbon C60**		**Animal numbers**				
**Organs**	**Observations**	**9684**	**9689**	**9690**	**9692**	**9695**
Adrenal glands	No lesion	+	+	+	+	+
Aorta	No lesion	+	+	+	+	+
Bone marrow	No lesion	+	+	+	+	+
Brain	No lesion	+	+	+	+	+
Cecum	No lesion	+	+	+	+	+
Colon	No lesion	+	+	+	+	+
Duodenum	No lesion	+	+	+	+	+
Eyes + optic nerve	No lesion	+	+	+	+	+
Esophagus	No lesion	+	+	+	+	+
Harderian glands	No lesion	+	+	+	+	+
Heart	No lesion	+	+	+	+	+
Ileum	No lesion	+	+	+	+	+
Jejunum	No lesion	+	+	+	+	+
Kidneys	No lesion	+	+	+	+	+
Lachrymal glands	No lesion	+	+	+	+	+
Liver	No lesion	+	+	+	+	+
**Lungs**	**Alveolar emphysema**	-	2	-	-	-
	**Hemorrhage**	-	1	-	-	-
Mammary gland	No lesion	+	+	+	+	+
Mesenteric lymph nodes	No lesion	+	+	+	+	+
Muscle (quadriceps)	No lesion	+	+	+	+	+
Nasal cavity	No lesion	+	+	+	+	+
Ovaries:	No lesion	+	+	+	+	+
Pancreas	No lesion	+	+	+	+	+
Pituitary	No lesion	+	+	+	+	+
Rectum	No lesion	+	+	+	+	+
Salivary glands (subm)	No lesion	+	+	+	+	+
Sciatic nerve	No lesion	+	+	+	+	+
Skin	No lesion	+	+	+	+	+
Spinal cord	No lesion	+	+	+	+	+
Spleen	No lesion	+	+	+	+	+
Sternum	No lesion	+	+	+	+	+
Stomach	No lesion	+	+	+	+	+
Subm. lymph nodes	No lesion	+	+	+	+	+
**Thymus**	**Hemorrhage**	2	-	-	-	-
Thyroid + parathyroid	No lesion	+	+	+	+	+
Trachea	No lesion	+	+	+	+	+
Urinary bladder	No lesion	+	+	+	+	+
**Uterus**	**Dilatation**	-	-	-	+	-
Vagina	No lesion	+	+	+	+	+

Remark:

+ = Observation present
- = Observation not present
/ = No data
subm. = Submandibular
b = both sides
o = one side

1 = Minimal
2 = Mild
3 = Moderate
4 = Severe (Marked)

APPENDIX 9

Copy of the Certificates of Analysis

Olive Oil infuse with Carbon C60

SES research

5999 WEST 34TH STREET, SUITE 106. HOUSTON TEXAS 77092

713-686-9662

FAX 713-686-9635

Certificate of Analysis # C688

Product Name	Olive Oil infuse with Carbon 60		
Product Code:	SE20-142		
Lot#	V01561		
Production Date	04/17/2020		
Expiration Date	04/17/2023		
**Items**	**Method**	**Specification**	**Results**
Appearance	Observation	Reddish Brown liquid	PASS
Odor	Smell	Faint oil odor	PASS
Fullerene content (mg/kg)	HPLC	800-870	830

Heavy Metals (mg/kg or ppm)		Method	Specification	Results
	Hg	Methods EAM 4.7	<0.010	PASS
	Pb	Methods EAM 4.7	<0.010	PASS
	As	Methods EAM 4.7	0.012	PASS
	Cd	Methods EAM 4.7	<0.010	PASS

Microbiological Specification	Quantity	Units	Method
Total Plate Count	<3000	CFU / g	USP <61>
Yeast & Mold	<300	CFU / g	AOAC
Coliforms	<3	MPN / g	FDA (BAM) Ch.4
E. Coli	Negative		USP<62>
Pseudomonas Aeruginosa	Negative		USP<62>
Salmonella	Negative		USP<62>
Staphylococcus aureus	Negative		USP<62>

SES Research
Owner

Mr. Robert Wong ______ 10/20/2020
date

Page 1 of 1

APPENDIX 9

Copy of the Certificates of Analysis

EVO Olive Oil

SES research

5999 WEST 34TH STREET, SUITE 106. HOUSTON TEXAS 77092

713-686-9662

FAX 713-686-9635

Certificate of Analysis # C685

Product Name	EVO Olive Oil		
Product Code:	SE20-EVOO		
Lot#	V100		
Production Date	04/17/2020		
Expiration Date	04/17/2023		
**Items**	**Method**	**Specification**	**Results**
Appearance	Observation	Golden greenish liquid	PASS
Odor	Smell	Faint oil odor	PASS
Fullerene content (mg/kg)	HPLC	0	0

Heavy Metals (mg/kg or ppm)		Method	Specification	Results
	Hg	Methods EAM 4.7	<0.010	PASS
	Pb	Methods EAM 4.7	<0.010	PASS
	As	Methods EAM 4.7	0.012	PASS
	Cd	Methods EAM 4.7	<0.010	PASS

Microbiological Specification	Quantity	Units	Method
Total Plate Count	<3000	CFU / g	USP <61>
Yeast & Mold	<300	CFU / g	AOAC
Coliforms	<3	MPN / g	FDA (BAM) Ch.4
E. Coli	Negative		USP<62>
Pseudomonas Aeruginosa	Negative		USP<62>
Salmonella	Negative		USP<62>
Staphylococcus aureus	Negative		USP<62>

SES Research
Owner

Mr. Robert Wong   10/20/2020
                               date

Page 1 of 1

APPENDIX 10

Copy of the Health Certificate of animals

TOXI-COOP Toxikológiai Kutató Központ ZRT

1103. Budapest, Cserkesz utca 90.

SPF HIGIÉNIAI STATUSZIGAZOLÁS

SPF HEALTH CERTIFICATE

Kórboctani/*Pathology* dátum/*date*: 2019. nov. 11.,12. / 11.,12. 11. 2019. E,P(M,R)

Kórszövettani/*Hystopathology* dátum/*date*: 2019. nov. 07./ 07. 11. 2019. P,E(R,M)

Parazitológia/*Parasitology* dátum/*date*: 2019. nov. 07./ 07. 11. 2019. P,E(R,M)

Bacteriológia/*Bacteriology* dátum/*date:* 2019. nov. 11./ *11.11..2019.* P,E(R,M)

Egér db /mouse piece: 2(M) *patkány db/rat piece:* 2 (R)

Kórokozó/agent	Össz.vizsgált minta<br>total tested	Legutóbbi eredmény<br>latest results
Salmonella spp.	00/ 20	00/4
Citrobacter rodentium	00/20	00/4
Bordetella bronchiseptica	00/ 20	00/4
Corynebacterium kutscheri	00/ 20	00/4
Clostridium piliforme	00/ 16	00/00
Pastorella spp.	00/ 24	00/4
Streptococcus spp.	00/ 20	00/4
S. pneumonie		
S. spp. β haemolitic		
Yersinia pseudotuberculosis	00/ 20	00/4
Mycoplasma spp.	00/ 24	00/4
Streptobacillus moniliformis	00/ 20	00/4
pseudomonas aeruginosa	00/ 20	00/4
Helicobacter spp.	00/ 20	00/4
Endoparazita	00/ 20	00/4
Ektoparazita	00/ 20	00/4
Protozoonok	00/ 20	00/4

Megjegyzés/*Comments:* A vizsgálatot 2019. november 15. **Nemzeti Élelmiszerlánc-biztonsági Hivatal Diagnosztikai Ig**. végezte.

Ikt.szám: **M 2019-10055030** patkány(**R**) és **M 2019-10055031** egér (M)

Dátum/Date: 2019. november. 25.

„TOXI-COOP" Toxikológiai Kutató Központ Zártkörűen Működő Részvénytársaság

Aláírás/Signatur

APPENDIX 10

Copy of the Health Certificate of animals

TOXI-COOP Toxikológiai Kutató Központ ZRT

1103. Budapest, Cserkesz utca 90.

SPF HIGIÉNIAI STATUSZIGAZOLÁS

SPF HEALTH CERTIFICATE

Serologia/ *Serology* dátum/*date*: 2021. január 11./ *11. Jan. 2021.*

Egér (M)db /mouse piece: 2 *patkány(R) db/rat piece:* 2

Kórokozó/agent	Módszer/Method		Össz.vizsgált minta total tested	Legutóbbi eredmény latest results
SDAV/RCV	IFA	R	00/14	00/02
MHV	IFA	M	00/14	00/02
Theiler(TMEV)	IFA	M	00/14	00/02
PVM	IFA	R,M	00/28	00/04
Sendai	IFA	R,M	00/28	00/04
MVM	IFA	M	00/14	00/02
MPV	IFA	M	00/14	00/02
Rat parvoviruses	IFA	R	00/14	00/02
Rota (EDIM)	IFA	M	00/14	00/02
MNV	IFA	M	00/14	00/02
Mycoplasma pulmonis	IFA	R,M	00/28	00/04
Clostridium piliforme	ELISA	R,M	00/28	00/04
Pasteurella pneumotropica	IFA	R,M	00/28	00/04
Rat theilovirus	IFA	R	00/06	00/02

Megjegyzés/*Comments:* A vizsgálatot 2021. január 11. ***BioDoc-Hannover***, Feor-Lynen Str. 23. D-30625 Hannover: ***40/WK/21*** témaszámon végezte.

Patkány=R; Egér=M; IFA= Indirect Immunofluorescence; ELISA= Enzime-linked immunosorbent assay

Dátum/Date: 2021. január 20.

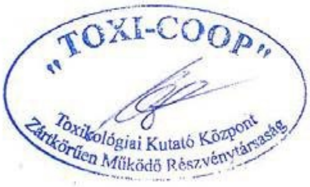

Aláírás/*Signature*

APPENDIX 11

Quality control of the diet

AGROLAB LUFA GmbH

Dr.-Hell-Str. 6, 24107 Kiel, Germany
www.agrolab.de

AGROLAB GROUP
Your labs. Your service.

AGROLAB LUFA Dr.-Hell-Str. 6, 24107 Kiel

SSNIFF SPEZIALDIÄTEN GMBH
FERDINAND-GABRIEL-WEG 16
59494 SOEST

ssniff Spezialdiäten GmbH
Freigabe / Release
Nach GV-Solas

MHD/best before: 31.05.21

The activities reported in this document are accredited according to DIN EN ISO/IEC 17025:2018. Only not accredited activities are identified by the symbol " *) ".

Date	04.12.2020
Customer no.	1000187

REPORT 2818859 - 862473

Order	**2818859 71101VD-20 088 Ssniff**
Sample no.	**862473**
Project	**145 ssniff**
Sample acceptance	**30.11.2020**
Date of sampling	**not specified**
Customer sample description	**SM Rat/Mouse- Zucht+ Haltung Ungarn, 10 mm<br>S8106-S008<br>Chargen-Nr.: 211 72850**
Packaging	**plastic bag**

	Unit	Result	Declaration	Substance	Method
**Nutrition values/ingredients**					
Moisture (4h, 103°C)	%	**11,8**		OM	REG(EC) 152/2009, III, A : 2009-02
Crude ash	%	**6,3**		OM	REG(EC) 152/2009, III, M : 2009-02
Crude protein (Nx6,25)	%	**20,5**		OM	REG(EC) 152/2009, III, C : 2009-02
Total fat	%	**3,9**		OM	REG(EC) 152/2009, III, H, method B : 2009-02
Crude fibre	%	**4,7**		OM	REG(EC) 152/2009, III, I : 2009-02
**Calculated values (nutrition/ingredients)**					
N-free substances	%	**52,8**		OM	calculated
**Minerals**					
Sodium (Na)	%	**0,21**		OM	DIN EN 15621 : 2017-10
Calcium (Ca)	%	**1,05**		OM	DIN EN 15621 : 2017-10
Phosphorus (P)	%	**0,75**		OM	DIN EN 15621 : 2017-10

Parameter-specific measurement uncertainties and information regarding the method of calculation will be provided upon request if the reported results are above the parameter-specific limit of quantification.

Explanation: OM = on original matter; DM = on dry matter base

Start of testing: 30.11.2020
End of testing: 04.12.2020

The results are related only to the samples tested. In cases where the laboratory has not been responsible for sampling, the reported results apply to the samples as received. Duplication of this document or of parts of it requires the authorization from laboratory. In accordance our agreement in writing in the order confirmation, the results in this test report are in a simplified form in the context of DIN EN ISO/IEC 17025:2018, paragraph 7.8.1.3.

F. Borchers

AGROLAB LUFA Frau Frederike Borchers, Tel. 0431/1228-210
Customer Relations Management feed

page 1 of 1

SignaturAG Kiel HRB 5796 Ust./VAT-ID-Nr: DE 813 356 511

Geschäftsführer
Dr. Paul Wimmer
Dr. Stephanie Nagorny

DAkkS Deutsche Akkreditierungsstelle D PL 14082 01 00

APPENDIX 11

Quality control of the diet

AGROLAB LUFA GmbH

Dr.-Hell-Str. 6, 24107 Kiel, Germany
www.agrolab.de

AGROLAB GROUP
Your labs. Your service.

AGROLAB LUFA Dr.-Hell-Str. 6, 24107 Kiel

SSNIFF SPEZIALDIÄTEN GMBH
FERDINAND-GABRIEL-WEG 16
59494 SOEST

ssniff Spezialdiäten GmbH
Freigabe / Release
Nach GV-Solas

MHD/best before:
31.05.21

Date 14.12.2020
Customer no. 1000187

The activities reported in this document are accredited according to DIN EN ISO/IEC 17025:2018. Only not accredited activities are identified by the symbol " *) ".

REPORT 2818877 - 862456

Order	**2818877 71101VD-20 088 Ssniff**
Sample no.	**862456**
Project	**145 ssniff**
Sample acceptance	**30.11.2020**
Date of sampling	**not specified**
Customer sample description	**SM Rat/Mouse- Zucht+ Haltung Ungarn, 10 mm<br>S8106-S008<br>Chargen-Nr.: 211 72850**
Packaging	**plastic bag**

	Unit	Result	Declaration	Substance	Method
**Trace elements / Heavy metals / Halogenides**					
Copper (Cu)	mg/kg	**11,7**		OM	DIN EN 15621 : 2017-10
Selenium (Se)	mg/kg	**0,23**		OM	DIN EN 17053 : 2018-03
Cadmium (Cd)	mg/kg	**0,06**		OM	DIN EN 17053 : 2018-03
Lead (Pb)	mg/kg	**<0,10**		OM	DIN EN 17053 : 2018-03
Mercury (Hg)	mg/kg	**<0,02**		OM	DIN EN 16277 : 2012-09 (mod.)
Arsenic (As)	mg/kg	**<0,10**		OM	DIN EN 17053 : 2018-03
**Pharmaceutical Substances**					
Antibiotic activity		**detected**		OM	VDLUFA III, 28.4.1 : 2007 (mod.)
Amoxycillin	mg/kg	**<0,05**		OM	MP-00238-DE : 2020-06 (LC-MSMS)
Ampicillin	mg/kg	**<0,05**		OM	MP-00238-DE : 2020-06 (LC-MSMS)
Chlortetracycline	mg/kg	**<0,05**		OM	MP-00238-DE : 2020-06 (LC-MSMS)
Doxycycline residues	mg/kg	**<0,05**		OM	MP-00238-DE : 2020-06 (LC-MSMS)
Florfenicol	mg/kg	**<0,01**		OM	MP-00238-DE : 2020-06 (LC-MSMS)
Lasalocid-Sodium	mg/kg	**<0,05**		OM	MP-00238-DE : 2020-06 (LC-MSMS)
Lincomycine	mg/kg	**<0,05**		OM	MP-00238-DE : 2020-06 (LC-MSMS)
Maduramicin-Ammonium	mg/kg	**<0,05**		OM	MP-00238-DE : 2020-06 (LC-MSMS)
Monensin-Sodium	mg/kg	**<0,05**		OM	MP-00238-DE : 2020-06 (LC-MSMS)
Narasine	mg/kg	**<0,05**		OM	MP-00238-DE : 2020-06 (LC-MSMS)
Nicarbazin	mg/kg	**<0,05**		OM	MP-00238-DE : 2020-06 (LC-MSMS)
Oxytetracycline (OTC)	mg/kg	**<0,05**		OM	MP-00238-DE : 2020-06 (LC-MSMS)
Salinomycin-Sodium	mg/kg	**<0,05**		OM	MP-00238-DE : 2020-06 (LC-MSMS)
Semduramicin-Sodium	mg/kg	**<0,05**		OM	MP-00238-DE : 2020-06 (LC-MSMS)
Spiramycin	mg/kg	**<0,05**		OM	MP-00238-DE : 2020-06 (LC-MSMS)
Sulfachinoxaline	mg/kg	**<0,05**		OM	MP-00238-DE : 2020-06 (LC-MSMS)

page 1 of 4

SignaturAG Kiel
HRB 5796
Ust./VAT-ID-Nr:
DE 813 356 511

Geschäftsführer
Dr. Paul Wimmer
Dr. Stephanie Nagorny

ilac-MRA DAkkS Deutsche Akkreditierungsstelle D-PL-14082-01-00

APPENDIX 11

Quality control of the diet

AGROLAB LUFA GmbH

Dr.-Hell-Str. 6, 24107 Kiel, Germany
www.agrolab.de

AGROLAB GROUP
Your labs. Your service.

ssniff
Spezialdiäten GmbH
Freigabe / Release
Nach GV-Solas

Date	14.12.2020
Customer no.	1000187

REPORT 2818877 - 862456

	Unit	Result	Declaration		Substance	Method
Sulfaclozine	mg/kg	**<0,05**			OM	MP-00238-DE : 2020-06 (LC-MSMS)
Sulfadiazine	mg/kg	**<0,05**			OM	MP-00238-DE : 2020-06 (LC-MSMS)
Sulfadimethoxine	mg/kg	**<0,05**			OM	MP-00238-DE : 2020-06 (LC-MSMS)
Sulfadimidine	mg/kg	**<0,05**			OM	MP-00238-DE : 2020-06 (LC-MSMS)
Sulfadoxine	mg/kg	**<0,05**			OM	MP-00238-DE : 2020-06 (LC-MSMS)
Sulfamerazine	mg/kg	**<0,05**			OM	MP-00238-DE : 2020-06 (LC-MSMS)
Sulfamethizole	mg/kg	**<0,05**			OM	MP-00238-DE : 2020-06 (LC-MSMS)
Sulfamethoxazole	mg/kg	**<0,05**			OM	MP-00238-DE : 2020-06 (LC-MSMS)
Sulfathiazole	mg/kg	**<0,05**			OM	MP-00238-DE : 2020-06 (LC-MSMS)
Tetracycline (TC)	mg/kg	**<0,05**			OM	MP-00238-DE : 2020-06 (LC-MSMS)
Tiamuline	mg/kg	**<0,05**			OM	MP-00238-DE : 2020-06 (LC-MSMS)
Trimethoprim	mg/kg	**<0,05**			OM	MP-00238-DE : 2020-06 (LC-MSMS)
Tylosin A	mg/kg	**<0,05**			OM	MP-00238-DE : 2020-06 (LC-MSMS)
Virginiamycin M1	mg/kg	**<0,05**			OM	MP-00238-DE : 2020-06 (LC-MSMS)
**Mycotoxins**						
Aflatoxine B1	µg/kg	**<0,5**			OM	QMP_504_KI_52_151 : 2020-11 (LC-MSMS)
Aflatoxine B2	µg/kg	**<0,5**			OM	QMP_504_KI_52_151 : 2020-11 (LC-MSMS)
Aflatoxine G1	µg/kg	**<0,5**			OM	QMP_504_KI_52_151 : 2020-11 (LC-MSMS)
Aflatoxine G2	µg/kg	**<0,5**			OM	QMP_504_KI_52_151 : 2020-11 (LC-MSMS)
**Sum aflatoxines**	µg/kg	**n.q.**			OM	calculated
**Non-dioxinlike PCB (ndl-PCB)**						
PCB 28	mg/kg	**<0,0002**			OM	DIN EN 16215 : 2012-07 (mod.)
PCB 52	mg/kg	**<0,0004**			OM	DIN EN 16215 : 2012-07 (mod.)
PCB 101	mg/kg	**<0,00055**			OM	DIN EN 16215 : 2012-07 (mod.)
PCB 138	mg/kg	**<0,0002**			OM	DIN EN 16215 : 2012-07 (mod.)
PCB 153	mg/kg	**<0,0002**			OM	DIN EN 16215 : 2012-07 (mod.)
PCB 180	mg/kg	**<0,0001**			OM	DIN EN 16215 : 2012-07 (mod.)
**Pesticides Multiresiduemethods**						
Aldrin	mg/kg	**<0,005**			OM	EN 15662 : 2018 (mod.)
Dieldrin	mg/kg	**<0,005**			OM	EN 15662 : 2018 (mod.)
**Sum aldrin, dieldrin**	mg/kg	**n.q.**			OM	calculated
Bromophos-ethyl	mg/kg	**<0,010**			OM	EN 15662 : 2018 (mod.)
Bromophos-methyl	mg/kg	**<0,010**			OM	EN 15662 : 2018 (mod.)
Carbophenothion	mg/kg	**<0,010**			OM	EN 15662 : 2018 (mod.)
Carbophenothion-methyl	mg/kg	**<0,010**			OM	EN 15662 : 2018 (mod.)
Chlordane alpha	mg/kg	**<0,005**			OM	EN 15662 : 2018 (mod.)
Chlordane gamma	mg/kg	**<0,005**			OM	EN 15662 : 2018 (mod.)
Chlordane oxy	mg/kg	**<0,005**			OM	EN 15662 : 2018 (mod.)
**Sum Chlordane**	mg/kg	**n.q.**			OM	calculated
Chlorphenvinphos	mg/kg	**<0,010**			OM	EN 15662 : 2018 (mod.)
Chlorpyrifos	mg/kg	**<0,010**			OM	EN 15662 : 2018 (mod.)
Chlorpyrifos-methyl	mg/kg	**<0,010**			OM	EN 15662 : 2018 (mod.)
Chlorthion	mg/kg	**<0,010**			OM	EN 15662 : 2018 (mod.)

The activities reported in this document are accredited according to DIN EN ISO/IEC 17025:2018. Only not accredited activities are identified by the symbol " *) ".

page 2 of 4

AG Kiel
HRB 5796
Ust./VAT-ID-Nr:
DE 813 356 511

Geschäftsführer
Dr. Paul Wimmer
Dr. Stephanie Nagorny

DAkkS
Deutsche
Akkreditierungsstelle
D-PL-14082-01-00

APPENDIX 11

Quality control of the diet

AGROLAB LUFA GmbH

Dr.-Hell-Str. 6. 24107 Kiel, Germany
www.agrolab.de

ssniff
Spezialdiäten GmbH
Freigabe / Release
Nach GV-Solas
RS

AGROLAB GROUP
Your labs. Your service.

Date 14.12.2020
Customer no. 1000187

REPORT 2818877 - 862456

	Unit	Result	Declaration		Substance	Method
o,p-DDD	mg/kg	**<0,005**			OM	EN 15662 : 2018 (mod.)
o,p-DDE	mg/kg	**<0,005**			OM	EN 15662 : 2018 (mod.)
o,p-DDT	mg/kg	**<0,005**			OM	EN 15662 : 2018 (mod.)
p,p-DDD	mg/kg	**<0,005**			OM	EN 15662 : 2018 (mod.)
p,p-DDE	mg/kg	**<0,005**			OM	EN 15662 : 2018 (mod.)
p,p-DDT	mg/kg	**<0,005**			OM	EN 15662 : 2018 (mod.)
**Sum DDT-isomers**	mg/kg	**n.q.**			OM	calculated
Diazinon	mg/kg	**<0,010**			OM	EN 15662 : 2018 (mod.)
Dichlorvos	mg/kg	**<0,010**			OM	EN 15662 : 2018 (mod.)
Dimethoate	mg/kg	**<0,010**			OM	EN 15662 : 2018 (mod.)
Endosulfan alpha	mg/kg	**<0,005**			OM	EN 15662 : 2018 (mod.)
Endosulfan beta	mg/kg	**<0,005**			OM	EN 15662 : 2018 (mod.)
Endosulfansulfat	mg/kg	**<0,005**			OM	EN 15662 : 2018 (mod.)
**Sum endosulfan-alpha, -beta, -sulfat**	mg/kg	**n.q.**			OM	calculated
Endrin	mg/kg	**<0,005**			OM	EN 15662 : 2018 (mod.)
Ethion	mg/kg	**<0,010**			OM	EN 15662 : 2018 (mod.)
Fenitrothion	mg/kg	**<0,010**			OM	EN 15662 : 2018 (mod.)
HCH-alpha	mg/kg	**<0,005**			OM	EN 15662 : 2018 (mod.)
HCH-beta	mg/kg	**<0,005**			OM	EN 15662 : 2018 (mod.)
HCH-delta	mg/kg	**<0,005**			OM	EN 15662 : 2018 (mod.)
HCH-epsilon	mg/kg	**<0,005**			OM	EN 15662 : 2018 (mod.)
Hexachlorobenzene	mg/kg	**<0,005**			OM	EN 15662 : 2018 (mod.)
**Sum alpha-, beta-, delta-, epsilon-HCH**	mg/kg	**n.q.**			OM	calculated
HCH-gamma (Lindane)	mg/kg	**<0,005**			OM	EN 15662 : 2018 (mod.)
Heptachlor	mg/kg	**<0,005**			OM	EN 15662 : 2018 (mod.)
Heptachlorepoxide-cis	mg/kg	**<0,005**			OM	EN 15662 : 2018 (mod.)
Heptachlorepoxide-trans	mg/kg	**<0,005**			OM	EN 15662 : 2018 (mod.)
**Sum heptachlor, heptachlorepoxide**	mg/kg	**n.q.**			OM	calculated
Malathion	mg/kg	**<0,010**			OM	EN 15662 : 2018 (mod.)
Mecarbame	mg/kg	**<0,010**			OM	EN 15662 : 2018 (mod.)
Methidathion	mg/kg	**<0,010**			OM	EN 15662 : 2018 (mod.)
Methoxychlor	mg/kg	**<0,005**			OM	EN 15662 : 2018 (mod.)
Mirex	mg/kg	**<0,005**			OM	EN 15662 : 2018 (mod.)
Nitrofen	mg/kg	**<0,005**			OM	EN 15662 : 2018 (mod.)
Parathion-methyl	mg/kg	**<0,010**			OM	EN 15662 : 2018 (mod.)
Parathion-ethyl	mg/kg	**<0,010**			OM	EN 15662 : 2018 (mod.)
Quintozene	mg/kg	**<0,005**			OM	EN 15662 : 2018 (mod.)
Phorate	mg/kg	**<0,010**			OM	EN 15662 : 2018 (mod.)
Pirimiphos-ethyl	mg/kg	**<0,010**			OM	EN 15662 : 2018 (mod.)
Pirimiphos-methyl	mg/kg	**0,10**			OM	EN 15662 : 2018 (mod.)
Profenofos	mg/kg	**<0,010**			OM	EN 15662 : 2018 (mod.)
Sulfotep	mg/kg	**<0,010**			OM	EN 15662 : 2018 (mod.)
Tecnazene	mg/kg	**<0,005**			OM	EN 15662 : 2018 (mod.)
Tetradifon	mg/kg	**<0,005**			OM	EN 15662 : 2018 (mod.)

Explanation: The symbol "<" or n.d. in the result column means, the substance concerned is not quantifiable at the limit of quantification shown opposite.
Parameter-specific measurement uncertainties and information regarding the method of calculation will be provided upon request if the reported results are above the parameter-specific limit of quantification.

Explanation: OM = on original matter; DM = on dry matter base

The activities reported in this document are accredited according to DIN EN ISO/IEC 17025:2018. Only not accredited activities are identified by the symbol " *) ".

page 3 of 4

SignaturAG Kiel
ungültig HRB 5796
Ust./VAT-ID-Nr:
DE 813 356 511

Geschäftsführer
Dr. Paul Wimmer
Dr. Stephanie Nagorny

ilac-MRA DAkkS Deutsche Akkreditierungsstelle D-PL-14082-01-00

APPENDIX 11

Quality control of the diet

AGROLAB LUFA GmbH

Dr.-Hell-Str. 6, 24107 Kiel, Germany
www.agrolab.de

AGROLAB GROUP
Your labs. Your service.

Date 14.12.2020
Customer no. 1000187

REPORT 2818877 - 862456

Qualitative detection of antibiotic activity using agar well diffusion method

Applied test microorganisms: Bacillus cereus ATCC 11778, Bacillus pumilus NCTC 8241, Bacillus subtilis TcR, Kocuria rhizophila ATCC 9341, Micrococcus luteus ATCC 10240, Staphylococcus aureus ATCC 6538P. Detectable antimicrobial agents: ß-Lactam-Antibiotika (Amoxicillin, Ampicillin, Penicillin), Lincomycin, macrolides (spiramycin, tylosin), tiamulin, virginiamycin, tetracyclines (chlortetracycline, doxycycline, oxytetracycline, tetracycline), trimethoprim (synergist sulfonamides), florfenicol, lasalocid, monensin, narasin, salinomycin. The presence of antimicrobial agents is indicated by inhibition growth zones. Growth of test microorganisms can also be inhibited by organic acids, essential oils, secondary metabolites of plants and yeasts, metal ions, antioxidants and preserving agents.
Remark to Sum aldrin, dieldrin: Aldrin and dieldrin combined expressed as dieldrin (F).
Remark to Sum Chlordane: Sum of cis-Chlordan and trans-Chlordan (F)(R).
Remark to Sum DDT-Isomers: Sum of p,p´-DDT, o,p´-DDT, p-p´-DDE and p,p´-TDE (DDD) expressed as DDT (F).
Remark to Sum endosulfan-alpha, -beta, -sulphate: Sum of alpha- and beta-isomers and endosulfan-sulphate expresses as endosulfan (F).
Remark to HCH-alpha: Hexachlorocyclohexane (HCH), alpha-isomer (F).
Remark to HCH-beta: Hexachlorocyclohexane (HCH), beta-isomer (F).
Remark to Sum alpha-,beta-,delta-epsilon-HCH: Hexachlorocyclohexane (HCH), sum of isomers, except the gamma isomer.
Remark to HCH-gamma (Lindane): Lindane (Gamma-isomer of hexachlorocyclohexane (HCH)) (F).
Remark to Sum heptachlor, heptachlorepoxide: Sum of heptachlor and heptachlor epoxide expressed as heptachlor (F).

Start of testing: 30.11.2020
End of testing: 14.12.2020

The results are related only to the samples tested. In cases where the laboratory has not been responsible for sampling, the reported results apply to the samples as received. Duplication of this document or of parts of it requires the authorization from laboratory. In accordance our agreement in writing in the order confirmation, the results in this test report are in a simplified form in the context of DIN EN ISO/IEC 17025:2018, paragraph 7.8.1.3.

F. Borchers

AGROLAB LUFA Frau Frederike Borchers, Tel. 0431/1228-210
Customer Relations Management feed

The activities reported in this document are accredited according to DIN EN ISO/IEC 17025:2018. Only not accredited activities are identified by the symbol " *) ".

page 4 of 4

Signatur ungültig
AG Kiel
HRB 5796
Ust./VAT-ID-Nr:
DE 813 356 511

Geschäftsführer
Dr. Paul Wimmer
Dr. Stephanie Nagorny

ilac-MRA DAkkS Deutsche Akkreditierungsstelle D-PL-14082-01-00

APPENDIX 12

Quality control of the drinking water

Government Office of Capital Budapest
Public Health Department
Váci út 174.
Budapest
H-1138 Hungary

Source of water sample:	Toxi-Coop Zrt.<br>Animal house, room no. 11<br>Berlini utca 47-49.<br>H-1045 Budapest<br>Hungary
Sample taken by:	Government Office of Capital Budapest, Public Health Department<br>Accredited sampling
Date of sampling:	October 07, 2020
Registry no.	2020/ 04519
Date of report:	October 12, 2020

Bacteriology:	
Coliform no.	0/ 100 mL
Escherichia coli	0/ 100 mL
Enterococcus	0/ 100 mL
Pseudomonas aeruginosa	0/ 100 mL
Total colony no. at 37 ºC	4/ 1 mL
Total colony no. at 22 ºC	0/ 1 mL

Water chemistry:		
Color:	Colorless	
Transparency:	0.19 FNU	
Odor:	0 Ball	
Permanganate index (KOI ps.):	0.47	mg/L
Chloride:	17	mg/L
Nitrate:	4.81	mg/L
Nitrite:	<0.01	mg/L
Ammonium:	<0.02	mg/L
Total hardness:	134	CaO mg/L
Iron:	<50	µg/L
Manganese:	<10	µg/L
Sulfate:	30.2	mg/L
Sp. Conductivity:	392	µS/cm (20 ºC)

Temperature:	21.4 ºC
pH	7.31

The water sample is suitable drinking water for human on the basis of the examined specifications. The qualification was made according to the threshold limits declared by the Decree of the Hungarian Government no. 201/2001(X.25).
(Translation of the original certificate; see pages 2 and 3 of Appendix 12)

APPENDIX 12

Quality control of the drinking water

1/2 oldal

BUDAPEST FŐVÁROS
KORMÁNYHIVATALA

NÉPEGÉSZSÉGÜGYI FŐOSZTÁLY

Ivóvíz vizsgálati jegyzőkönyv

Iktatószám:	**2020/ 04519** Kód:	Megrendelő:	
Minta származása:	Toxi - Coop Zrt. 5/Á épület 2. em. 11. állatszoba<br>mosogató<br>ellenőrző<br>1045 Budapest, Berlini út 47-49.	Toxi - Coop Zrt.<br>Budapest<br>Magyar Jakobinusok tere 4. B. 5/2<br>11222	
A mintát vette:	BFKH NF Laboratóriumi Osztály<br>Akkreditált mintavétel		

Mintavétel: 2020. 10. 07. 08:40 Átvétel: 2020. 10. 07. Vizsgálat időtartama: 2020. 10. 07. - 10.12. Kiadás: 2020. 10. 12.

Helyszíni vizsgálatok

Min	Vizsgálat	Érték	Mértékegység	Szabvány
	Víz hőmérséklet	21,4	°C	MSZ 448-2 :1967 1.fejezet (visszavont szabvány)
	pH (25 °C)	7,31		MSZ 1484-22:2009 8.1. szakasz

Bakteriológiai vizsgálatok:

Min	Vizsgálat	Érték	Mértékegység	Szabvány
	Coliformszám	0	/100ml	MSZ EN ISO 9308-1:2015
	Escherichia coli-szám	0	/100ml	MSZ EN ISO 9308-1:2015
	Enterococcus-szám	0	/100mL	MSZ EN ISO 7899-2:2000
	Pseudomonas aeruginosa-szám	0	/100 mL	MSZ EN ISO 16266:2008
	Telepszám 37°C-on	4	/1mL	MSZ EN ISO 6222: 2000
	Telepszám 22°C-on	0	/1mL	MSZ EN ISO 6222: 2000

Kémiai vizsgálatok:

Min	Vizsgálat	Érték	Mértékegység	Szabvány
	# Szín	színtelen		MSZ EN ISO 7887:1998 2. fejezet (visszavont szabvány)
	# Zavarosság	0,19	FNU	MSZ EN ISO 7027-1:2016
	# Szag	0	Ball	MSZ 448-35:1965 2.1 szakasz (visszavont szabvány)
	Kémiai Oxigénigény KOIp	0,47	mg/l	MSZ 448-20:1990
	Klorid	17	mg/l	MSZ 1484-15:2009
	Nitrát	4,81	mg/l	MSZ 1484-13:2009 5.fejezet
	Nitrit	<0.01	mg/l	MSZ 1484-13:2009 6.fejezet
	Ammónium	<0.02	mg/l	MSZ ISO 7150-1:1992
	Össz. keménység	134	CaO mg/l	MSZ 448-21:1986 3. fejezet
	Vas	<50	µg/l	MSZ 448-4:1983 2.fejezet (visszavont szabvány)
	Mangán	<10	µg/l	MSZ 1484-2:1993
	Szulfát	30,2	mg/l	MSZ 448-13:1983 6. fejezet
	Fajl. elektr. vezetőképesség	392	µS/cm (20 °C)	MSZ EN 27888:1998

Laboratóriumi Osztály
1138 Budapest, Váci út 174. -1550 Budapest, Pf. 203. - Telefon: +36 (1) 465-3829
E-mail: kozeg.biolabor@nfo.bfkh.gov.hu - Honlap: www.kormanyhivatal.hu

A NAH által NAH-1-1362/2019 számon akkreditált vizsgálólaboratórium.

APPENDIX 12

Quality control of the drinking water

BUDAPEST FŐVÁROS
KORMÁNYHIVATALA

NÉPEGÉSZSÉGÜGYI FŐOSZTÁLY

Ivóvíz vizsgálati jegyzőkönyv

Iktatószám: **2020/ 04519** Kód: Megrendelő:

Minta származása: Toxi - Coop Zrt. 5/Á épület 2. em. 11. állatszoba mosogató ellenőrző 1045 Budapest, Berlini út 47-49.

Megrendelő: Toxi - Coop Zrt. Budapest Magyar Jakobinusok tere 4. B. 5/2 11222

A mintát vette: BFKH NF Laboratóriumi Osztály Akkreditált mintavétel

Mintavétel: 2020. 10. 07. 08:40 Átvétel: 2020. 10. 07. Vizsgálat időtartama: 2020. 10. 07. - 10.12. Kiadás: 2020. 10. 12.

A vízminta a vizsgált jellemzők szempontjából megfelelő ivóvíz.
Minősítés a 201/2001. (X.25.) kormányrendeletben foglalt határértékek szerint.
# - Nem akkreditált vizsgálat.
A mintavétel szakszerűségéért és a minta azonosságáért a mintavevő vállal felelősséget.
A közölt vizsgálati eredmények kizárólag a vizsgált mintára vonatkoznak.
A Vizsgálati jegyzőkönyvet a vizsgáló laboratórium engedélye nélkül csak teljes terjedelmében lehet másolni, kivonatolásához a kiadványozó írásos engedélye szükséges.
Aláírással, pecsét nélkül hiteles.

Budapest, 2020. 10. 12.

dr. Sára Botond
kormánymegbízott megbízásából

Vargáné Konyha Edit
kémiai laboratóriumi irányító

Dr. Kovács Gábor
osztályvezető

Laboratóriumi Osztály
1138 Budapest, Váci út 174. -1550 Budapest, Pf. 203. - Telefon: +36 (1) 465-3829
E-mail: kozeg.biolabor@nfo.bfkh.gov.hu - Honlap: www.kormanyhivatal.hu

A NAH által NAH-1-1362/2019 számon akkreditált vizsgálólaboratórium.

APPENDIX 13

Copy of the Certificate of Quality of the bedding material

J. RETTENMAIER & SÖHNE
GMBH + CO KG

Fasern aus der Natur
Fibers designed by Nature

73494 Rosenberg (Germany)
Phone: + 49 (0) 7967 / 152-0
Fax: + 49 (0) 7967 - 152 222
info@jrs.de
www.jrs.de

J. RETTENMAIER & SÖHNE • 73494 Rosenberg (Germany)

Certificate of Quality

TO WHOM IT MAY CONCERN

**Customer:**	**Toxi-Coop**<br>**Safety Toxicol. Study Center**<br>**Herr Zoltán Balogh**<br>**Cserkesz utca 90**<br>**H 1103 Budapest**
**Product:**	**SAFE 3/ 4-S-FASERN**
**Lot number:**	**03027201125 = 6.750,00 kg**
**Quantity:**	540 bags á 12,50 kg<br>20 palets á 337,50 kg
**Net weight:**	11.137,50 kg
**Country of origin:**	Federal Republic of Germany
**Manufacturing date:**	2020-11-11
**Best before:**	**at least 3 years from manufacturing date**

We hereby certify that goods are of first-class quality

Goods are harmless to people and safe from the ecological point of view.

With the comminution and faxtitious subsequent drying of the product +180° Celcius are obtained so that it can be supposed that the dispatch is free from hazardous plant diseases and vermines.

Holzmühle, 2020-12-02

J. RETTENMAIER & SÖHNE GMBH & CO. KG
Fibers designed by Nature

Elke Voss

APPENDIX 14

Historical control

Hematology and blood coagulation
Han:WIST Rat Male: 9 - 10 weeks old

Animal number	WBC [x10^9/L]	NEU [%]	LYM [%]	MONO [%]	EOS [%]	BASO [%]	RBC [x10^{12}/L]	HGB [g/L]	HCT [L/L]	MCV [fL]	MCH [pg]	MCHC [g/L]	PLT [x10^9/L]	RET [%]	PT [sec]	APTT [sec]
1760	4,9	15,3	80,2	2,6	1,6	0,0	7,93	160	0,46	57,5	20,2	351	1036	2,95	10,1	11,1
1762	6,1	15,2	80,7	3,0	0,9	0,0	7,80	159	0,45	57,5	20,3	353	974	3,39	10,7	12,9
1771	7,9	10,2	87,0	1,6	0,8	0,1	8,60	167	0,48	56,1	19,4	346	892	2,89	-	-
1775	7,6	13,4	82,7	2,0	1,2	0,1	8,19	166	0,48	59,0	20,3	344	1039	4,04	10,2	13,1
1780	7,9	12,2	82,5	3,3	1,3	0,0	8,16	169	0,48	59,4	20,7	348	1072	3,39	10,6	15,2
1814	10,5	7,6	88,5	2,3	1,0	0,1	8,49	164	0,48	56,2	19,3	343	864	2,37	10,3	9,9
1815	10,1	8,6	88,8	2,0	0,4	0,0	8,36	165	0,47	56,8	19,8	348	973	2,98	10,4	12,8
1822	9,6	9,5	87,8	1,6	0,7	0,1	8,11	169	0,47	58,4	20,8	356	912	2,99	10,2	15,8
1823	7,2	15,3	81,9	1,8	0,6	0,1	8,47	168	0,48	56,6	19,8	351	942	3,26	10,0	11,6
1824	11,2	10,7	86,5	1,6	0,9	0,0	8,36	170	0,48	57,5	20,3	353	1066	2,72	10,4	15,1
1093	9,7	9,7	87,8	1,3	0,6	0,2	8,26	172	0,49	59,6	20,8	349	918	2,99	10,0	13,8
1094	8,9	8,3	89,0	1,6	0,8	0,1	8,16	164	0,48	58,9	20,1	341	990	3,47	9,8	11,1
1098	8,2	13,1	84,2	1,5	0,8	0,1	7,85	158	0,45	57,7	20,1	348	1069	3,39	10,0	13,4
1102	9,5	13,9	82,7	2,0	0,9	0,0	8,00	165	0,48	59,8	20,6	344	1100	3,86	10,0	10,5
1107	8,3	15,5	80,4	2,9	0,7	0,1	8,49	164	0,48	56,7	19,4	341	780	3,85	10,0	12,8
596	9,7	11,8	84,7	1,8	0,8	0,1	8,76	167	0,50	57,1	19,1	334	1059	2,99	9,9	12,6
599	5,0	†45.8	†39.2	†6.6	7,9	0,2	8,33	151	0,45	54,4	18,1	334	638	2,58	10,1	14,3
601	7,8	16,2	78,8	2,7	1,6	0,1	8,14	164	0,48	58,5	20,2	345	1041	3,06	10,0	10,9
604	9,3	10,1	86,9	2,0	0,6	0,1	8,58	163	0,48	56,0	19,1	340	816	3,02	10,2	13,7
605	8,3	12,1	82,6	1,7	3,0	0,1	8,24	158	0,47	57,3	19,2	335	824	2,83	9,8	11,6
Mean	8,4	12,0	84,4	2,1	1,4	0,1	8,26	164,2	0,47	57,6	19,9	345,2	950,3	3,15	10,1	12,7
SD	1,7	2,7	3,3	0,6	1,6	0,1	0,26	5,0	0,01	1,4	0,7	6,4	120,6	0,43	0,2	1,7
n	20	19	19	19	20	20	20	20	20	20	20	20	20	20	19	19
Min	4,9	7,6	78,8	1,3	0,4	0,0	7,80	151	0,45	54,4	18,1	334	638	2,37	9,8	9,9
Max	11,2	16,2	89,0	3,3	7,9	0,2	8,76	172	0,50	59,8	20,8	356	1100	4,04	10,7	15,8

Remark: - = No data (coagulated sample)
† = Excluded from the evaluation

Historical control - Collected data - 2018; Equipments: Hematology, Siemens ADVIA120; Blood coagulation, Sysmex CA-1500

APPENDIX 14

Historical control

Hematology and blood coagulation
Han:WIST Rat Female: 9 - 10 weeks ol

Animal number	WBC [x10^9/L]	NEU [%]	LYM [%]	MONO [%]	EOS [%]	BASO [%]	RBC [x10^{12}/L]	HGB [g/L]	HCT [L/L]	MCV [fL]	MCH [pg]	MCHC [g/L]	PLT [x10^9/L]	RET [%]	PT [sec]	APTT [sec]
1794	5,5	10,6	85,9	1,5	1,2	0,1	7,74	157	0,46	60,0	20,4	340	994	3,42	9,7	10,0
1796	5,8	11,0	84,1	2,0	2,5	0,1	8,83	172	0,49	55,5	19,5	351	1099	2,18	9,7	11,0
1797	3,5	12,8	83,3	1,6	1,7	0,0	8,17	166	0,48	58,8	20,3	345	792	3,28	9,6	12,7
1801	5,0	12,8	82,8	2,1	1,6	0,1	8,12	162	0,45	55,9	19,9	356	1140	3,16	9,7	9,6
1802	7,1	11,0	86,0	1,7	0,8	0,1	7,78	157	0,44	56,1	20,1	359	768	3,13	10,0	12,7
1836	5,6	8,5	88,8	1,5	0,9	0,2	8,59	164	0,47	55,2	19,1	345	1004	1,86	9,7	13,1
1841	4,6	10,6	85,6	2,0	1,3	0,1	7,47	145	0,41	55,5	19,5	351	915	2,54	9,5	19,1
1843	5,9	26,9	67,7	3,2	1,9	0,1	8,15	165	0,48	59,3	20,2	341	1000	2,63	10,0	12,3
1845	7,4	14,9	80,2	2,9	1,1	0,1	7,92	164	0,46	57,5	20,8	361	1230	2,23	10,0	15,4
1858	7,4	6,7	90,4	1,8	0,6	0,0	8,69	167	0,48	55,0	19,2	349	993	2,35	10,0	14,4
1120	5,9	9,6	86,8	1,2	1,9	0,1	8,14	161	0,46	56,1	19,7	352	772	3,44	9,7	13,1
1128	4,8	13,0	83,5	1,2	1,7	0,1	8,48	163	0,46	54,4	19,2	352	1049	2,71	9,9	13,1
1130	8,3	17,2	77,4	2,6	2,2	0,1	8,85	162	0,47	53,4	18,3	344	785	2,01	9,8	12,8
1131	5,8	7,0	90,6	0,9	0,9	0,0	8,39	150	0,44	52,6	17,8	339	991	2,79	9,5	12,6
1136	6,3	6,5	90,8	1,4	0,6	0,0	8,05	153	0,44	55,0	19,0	345	994	3,23	9,8	14,7
612	6,0	14,4	79,5	1,2	4,4	0,1	7,62	147	0,44	57,9	19,3	334	1134	2,87	9,5	12,8
617	5,4	12,6	82,6	2,9	1,1	0,0	8,06	150	0,45	56,2	18,7	332	956	1,97	9,5	14,6
621	4,7	13,4	82,5	2,1	1,6	0,1	8,24	152	0,45	55,2	18,4	334	799	2,41	9,6	11,8
622	6,0	17,7	78,5	2,2	1,2	0,0	8,20	154	0,46	56,4	18,8	332	858	2,27	9,5	14,9
629	5,2	14,7	81,6	2,1	1,2	0,0	8,28	157	0,48	58,0	19,0	327	797	2,67	9,5	13,8
Mean	5,8	12,6	83,4	1,9	1,5	0,1	8,19	158,4	0,46	56,2	19,4	344,5	953,5	2,66	9,7	13,2
SD	1,1	4,6	5,4	0,6	0,9	0,1	0,38	7,3	0,02	1,9	0,8	9,6	138,8	0,50	0,2	2,1
n	20	20	20	20	20	20	20	20	20	20	20	20	20	20	20	20
Min	3,5	6,5	67,7	0,9	0,6	0,0	7,47	145	0,41	52,6	17,8	327	768	1,86	9,5	9,6
Max	8,3	26,9	90,8	3,2	4,4	0,2	8,85	172	0,49	60,0	20,8	361	1230	3,44	10,0	19,1

Historical control - Collected data - 2018; Equipments: Hematology, Siemens ADVIA120; Blood coagulation, Sysmex CA-1500

APPENDIX 14

Historical control

Clinical chemistry

Han:WIST Rat Male: 9 - 10 weeks old

Animal number	ALT [U/L]	AST [U/L]	GGT [U/L]	ALP [U/L]	TBIL [µmol/L]	CREA [µmol/L]	UREA [mmol/L]	GLUC [mmol/L]	CHOL [mmol/L]	Pi [mmol/L]	Ca^{++} [mmol/L]	Na^{+} [mmol/L]	K^{+} [mmol/L]	Cl [mmol/L]	ALB [g/L]	TPROT [g/L]	A/G
1760	45	105	bql	182	1,4	19	5,9	4,24	1,77	3,18	2,64	144,3	5,27	98,8	41,8	55,5	3,1
1762	50	114	bql	230	1,5	23	7,2	4,07	1,68	3,09	2,63	144,3	4,90	97,6	43,0	57,5	3,0
1771	47	100	bql	123	0,8	21	8,0	4,83	2,32	2,76	2,83	143,6	4,77	98,3	44,7	63,4	2,4
1775	44	106	bql	153	1,6	29	10,3	3,99	2,52	3,03	2,77	144,5	5,28	98,3	44,6	63,1	2,4
1780	50	108	bql	129	2,3	23	6,7	3,95	2,42	2,51	2,68	145,5	4,43	99,4	44,4	60,4	2,8
1814	56	136	bql	173	2,2	21	6,1	4,40	2,55	3,12	2,81	142,8	5,61	98,9	42,1	58,7	2,5
1815	47	89	bql	123	1,8	19	6,1	4,04	2,06	3,20	2,76	143,3	4,83	98,3	42,7	62,2	2,2
1822	44	95	bql	194	1,7	23	8,2	4,48	3,01	2,89	2,83	144,5	4,47	96,9	44,1	59,7	2,8
1823	51	81	bql	184	1,1	20	8,7	5,43	2,73	3,10	2,96	142,9	4,71	94,5	45,0	62,7	2,5
1824	48	100	bql	239	2,2	22	6,8	4,48	2,43	2,89	2,70	143,7	4,25	97,9	45,8	63,4	2,6
1093	56	93	bql	230	1,6	20	7,3	5,75	2,22	3,11	2,80	144,2	4,50	97,0	44,5	62,9	2,4
1094	53	104	bql	242	1,5	23	6,6	5,34	2,86	2,52	2,63	143,9	4,40	99,3	41,4	59,8	2,3
1098	41	89	bql	249	1,5	21	6,0	5,94	2,14	3,09	2,71	144,5	4,74	98,1	43,9	61,3	2,5
1102	54	89	bql	208	2,2	23	9,4	5,56	1,94	3,31	2,77	147,6	5,19	101,8	43,2	62,7	2,2
1107	51	87	bql	262	1,5	19	5,6	4,66	2,61	2,98	2,82	143,7	4,83	99,1	41,7	59,6	2,3
596	47	102	bql	169	1,6	17	9,2	5,70	2,71	2,91	2,81	141,6	4,86	96,1	43,6	63,3	2,2
599	†179	†368	bql	212	1,3	21	9,5	5,25	2,20	2,83	2,76	141,4	4,87	97,9	39,9	59,7	2,0
601	61	96	bql	131	1,4	20	10,9	5,73	2,38	3,21	2,77	141,9	5,45	97,8	42,0	60,4	2,3
604	35	72	bql	196	1,7	20	6,7	4,83	1,84	3,16	2,63	143,9	4,56	99,1	41,5	57,7	2,6
605	54	92	bql	173	1,3	18	8,7	5,22	2,42	3,06	2,82	144,4	4,59	99,4	43,0	59,9	2,5
Mean	49,2	97,8	-	190,1	1,6	21,1	7,7	4,9	2,3	3,0	2,8	143,8	4,8	98,2	43,1	60,7	2,5
SD	6,0	13,7	-	43,8	0,4	2,6	1,6	0,7	0,4	0,2	0,1	1,4	0,4	1,5	1,5	2,3	0,3
n	19	19	-	20	20	20	20	20	20	20	20	20	20	20	20	20	20
Min	35	72	-	123	0,8	17	5,6	4,0	1,7	2,5	2,6	141,4	4,3	94,5	39,9	55,5	2,0
Max	61	136	-	262	2,3	29	10,9	5,9	3,0	3,3	3,0	147,6	5,6	101,8	45,8	63,4	3,1

Remark: bql = Below the quantification limit - 7 U/L
- = No data
† = Excluded from the evaluation

Historical control - Collected data - 2018; Equipment: Cobas C311

APPENDIX 14

Historical control

Clinical chemistry
Han:WIST Rat Female: 9 - 10 weeks old

Animal number	ALT [U/L]	AST [U/L]	GGT [U/L]	ALP [U/L]	TBIL [µmol/L]	CREA [µmol/L]	UREA [mmol/L]	GLUC [mmol/L]	CHOL [mmol/L]	Pi [mmol/L]	Ca^{++} [mmol/L]	Na^{+} [mmol/L]	K^{+} [mmol/L]	Cl^{-} [mmol/L]	ALB [g/L]	TPROT [g/L]	A/G
1794	38	145	bql	188	0,9	31	8,0	3,28	2,02	2,81	2,54	147,3	4,08	103,7	44,2	58,7	3,0
1796	26	87	bql	179	1,5	22	5,8	3,89	2,20	2,71	2,62	144,9	4,27	100,2	47,1	61,7	3,2
1797	39	134	bql	121	1,2	25	5,9	3,40	2,07	2,71	2,69	146,2	4,41	99,1	47,6	61,8	3,4
1801	36	110	bql	129	2,0	27	6,5	3,62	1,89	2,80	2,61	139,8	4,35	98,2	48,2	60,8	3,8
1802	42	100	bql	122	1,0	26	6,1	4,20	1,68	2,48	2,63	142,3	3,69	98,7	45,5	60,5	3,0
1836	30	85	bql	143	1,3	26	6,6	4,22	1,71	2,41	2,66	143,7	4,37	100,3	46,5	61,7	3,1
1841	38	131	bql	101	1,6	32	12,2	4,69	2,54	2,72	2,69	145,6	4,19	100,1	47,2	67,9	2,3
1843	42	111	bql	129	1,7	32	9,0	4,34	2,24	2,69	2,59	144,8	4,54	97,0	45,5	61,1	2,9
1845	30	83	bql	108	1,2	25	7,5	4,21	2,17	2,23	2,63	143,3	4,15	99,6	45,5	61,8	2,8
1858	37	79	bql	75	1,1	24	7,0	5,50	2,27	2,14	2,63	144,4	4,19	100,6	47,1	62,9	3,0
1120	36	98	bql	134	1,1	26	6,9	4,56	1,85	2,37	2,65	144,6	4,74	101,2	44,3	60,6	2,7
1128	54	93	bql	172	1,7	23	4,8	5,59	1,47	2,04	2,60	144,3	3,98	100,8	47,0	65,0	2,6
1130	60	91	bql	133	1,7	32	7,8	4,38	1,40	2,33	2,58	143,4	3,92	101,5	43,4	59,1	2,8
1131	53	126	bql	87	1,0	25	7,2	3,97	1,77	2,18	2,59	144,2	3,87	103,4	44,6	62,6	2,5
1136	39	89	bql	102	1,5	29	6,3	5,13	1,41	1,82	2,65	144,5	3,98	101,1	50,3	68,4	2,8
612	32	67	bql	66	2,0	21	7,2	5,45	1,95	2,43	2,73	142,4	4,27	101,2	49,5	65,3	3,1
617	34	91	bql	152	1,1	23	6,3	4,56	2,09	2,82	2,61	141,8	4,56	99,9	42,4	60,3	2,4
621	35	85	bql	168	1,8	23	6,4	4,94	2,03	2,55	2,60	141,9	4,25	99,8	45,2	62,7	2,6
622	47	81	bql	101	0,9	22	6,0	6,18	2,06	2,42	2,58	142,2	3,76	100,7	42,0	61,0	2,2
629	40	73	bql	117	1,1	21	7,1	5,88	2,45	2,78	2,70	141,6	4,12	99,2	45,0	65,8	2,2
Mean	39,4	98,0	-	126,4	1,4	25,8	7,0	4,6	2,0	2,5	2,6	143,7	4,2	100,3	45,9	62,5	2,8
SD	8,5	21,5	-	33,7	0,4	3,7	1,5	0,8	0,3	0,3	0,0	1,8	0,3	1,6	2,2	2,7	0,4
n	20	20	-	20	20	20	20	20	20	20	20	20	20	20	20	20	20
Min	26	67	-	66	0,9	21,0	4,8	3,3	1,4	1,8	2,5	139,8	3,7	97,0	42,0	58,7	2,2
Max	60	145	-	188	2,0	32,0	12,2	6,2	2,5	2,8	2,7	147,3	4,7	103,7	50,3	68,4	3,8

Remark: bql = Below the quantification limit - 7 U/L
- = No data

Historical control - Collected data - 2018; Equipment: Cobas C311

APPENDIX 14

Historical control

Organ weight
Han:WIST Rat Male: 9 - 10 weeks old

Animal number	Body weight	Organ weight (g) Brain	Liver	Kidneys	Heart	Thymus	Spleen	Testes	Epididymides	Seminal vesicles† Prostate	Adrenal glands
1760	265	1,97	8,18	2,07	0,81	0,76	0,77	3,10	0,74	1,20	0,067
1762	251	2,06	7,09	1,85	0,80	0,71	0,49	3,23	0,75	1,24	0,050
1771	249	2,04	7,80	1,69	0,70	0,50	0,55	2,86	0,70	0,99	0,053
1775	265	1,97	8,53	2,21	0,88	0,80	0,66	2,94	0,84	0,87	0,087
1780	259	1,95	7,71	2,18	0,94	0,93	0,70	3,18	0,66	1,01	0,073
1814	279	1,90	9,35	2,36	0,87	0,75	0,69	2,84	0,80	1,25	0,064
1815	269	1,92	8,58	2,55	0,83	0,76	0,59	3,17	0,91	1,31	0,072
1822	267	2,06	7,56	2,05	0,80	0,66	0,70	3,03	0,80	0,89	0,070
1823	268	2,06	9,57	1,93	0,86	0,62	0,60	3,06	0,74	0,89	0,069
1824	258	2,00	8,04	1,98	0,82	0,68	0,69	3,03	0,90	1,01	0,070
1093	281	1,95	9,24	2,10	0,88	0,49	0,61	3,05	0,90	1,26	0,083
1094	286	2,10	8,73	2,25	0,85	0,56	0,57	3,08	0,84	1,29	0,078
1098	294	2,00	9,11	2,10	0,95	0,50	0,54	2,96	0,73	1,39	0,079
1102	283	2,05	9,32	2,18	0,85	0,61	0,66	3,06	0,78	1,28	0,063
1107	308	2,02	9,49	2,25	0,96	0,66	0,73	3,22	0,74	1,23	0,064
596	290	2,06	8,57	2,51	0,89	0,52	0,66	3,54	1,07	1,25	0,069
599	306	2,21	9,40	2,16	0,93	0,80	0,53	3,22	0,99	1,20	0,078
601	316	2,06	9,92	2,17	0,98	0,62	0,65	3,33	0,97	1,15	0,076
604	314	2,14	8,98	2,33	0,90	0,63	0,71	3,15	0,96	1,25	0,076
605	289	2,10	8,58	2,09	0,87	0,59	0,62	3,45	0,92	0,86	0,076
Mean	279,9	2,03	8,69	2,15	0,87	0,66	0,64	3,13	0,84	1,14	0,071
SD	20,5	0,08	0,77	0,20	0,07	0,12	0,07	0,18	0,11	0,17	0,009
n	20	20	20	20	20	20	20	20	20	20	20
Min	249,0	1,90	7,09	1,69	0,70	0,49	0,49	2,84	0,66	0,86	0,050
Max	316,0	2,21	9,92	2,55	0,98	0,93	0,77	3,54	1,07	1,39	0,087

† = Seminal vesicles with coagulating gland

Historical control - Collected data - 2018

APPENDIX 14

Historical control

Organ weight
Han:WIST Rat Male: 9 - 10 weeks old

Animal number	Brain	Liver	Kidneys	Heart	Thymus	Spleen	Testes	Epididy-mides	Seminal vesicles† Prostate	Adrenal glands
	Organ weight relative to body weight (%)									
1760	0,74	3,09	0,78	0,31	0,29	0,29	1,17	0,28	0,45	0,025
1762	0,82	2,82	0,74	0,32	0,28	0,20	1,29	0,30	0,49	0,020
1771	0,82	3,13	0,68	0,28	0,20	0,22	1,15	0,28	0,40	0,021
1775	0,74	3,22	0,83	0,33	0,30	0,25	1,11	0,32	0,33	0,033
1780	0,75	2,98	0,84	0,36	0,36	0,27	1,23	0,25	0,39	0,028
1814	0,68	3,35	0,85	0,31	0,27	0,25	1,02	0,29	0,45	0,023
1815	0,71	3,19	0,95	0,31	0,28	0,22	1,18	0,34	0,49	0,027
1822	0,77	2,83	0,77	0,30	0,25	0,26	1,13	0,30	0,33	0,026
1823	0,77	3,57	0,72	0,32	0,23	0,22	1,14	0,28	0,33	0,026
1824	0,78	3,12	0,77	0,32	0,26	0,27	1,17	0,35	0,39	0,027
1093	0,69	3,29	0,75	0,31	0,17	0,22	1,09	0,32	0,45	0,030
1094	0,73	3,05	0,79	0,30	0,20	0,20	1,08	0,29	0,45	0,027
1098	0,68	3,10	0,71	0,32	0,17	0,18	1,01	0,25	0,47	0,027
1102	0,72	3,29	0,77	0,30	0,22	0,23	1,08	0,28	0,45	0,022
1107	0,66	3,08	0,73	0,31	0,21	0,24	1,05	0,24	0,40	0,021
596	0,71	2,96	0,87	0,31	0,18	0,23	1,22	0,37	0,43	0,024
599	0,72	3,07	0,71	0,30	0,26	0,17	1,05	0,32	0,39	0,025
601	0,65	3,14	0,69	0,31	0,20	0,21	1,05	0,31	0,36	0,024
604	0,68	2,86	0,74	0,29	0,20	0,23	1,00	0,31	0,40	0,024
605	0,73	2,97	0,72	0,30	0,20	0,21	1,19	0,32	0,30	0,026
Mean	0,73	3,11	0,77	0,31	0,24	0,23	1,12	0,30	0,41	0,025
SD	0,05	0,18	0,07	0,02	0,05	0,03	0,08	0,03	0,06	0,003
n	20	20	20	20	20	20	20	20	20	20
Min	0,65	2,82	0,68	0,28	0,17	0,17	1,00	0,24	0,30	0,020
Max	0,82	3,57	0,95	0,36	0,36	0,29	1,29	0,37	0,49	0,033

† = Seminal vesicles with coagulating gland

Historical control - Collected data - 2018

APPENDIX 14

Historical control

Organ weight
Han:WIST Rat Male: 9 - 10 weeks old

Animal number	Body weight	Liver	Kidneys	Heart	Thymus	Spleen	Testes	Epididy-mides	Seminal vesicles† Prostate	Adrenal glands
	Organ weight and body weight relative to brain weight (%)									
1760	13452	415	105	41,1	38,6	39,1	157	37,56	60,9	3,4
1762	12184	344	90	38,8	34,5	23,8	157	36,41	60,2	2,4
1771	12206	382	83	34,3	24,5	27,0	140	34,31	48,5	2,6
1775	13452	433	112	44,7	40,6	33,5	149	42,64	44,2	4,4
1780	13282	395	112	48,2	47,7	35,9	163	33,85	51,8	3,7
1814	14684	492	124	45,8	39,5	36,3	149	42,11	65,8	3,4
1815	14010	447	133	43,2	39,6	30,7	165	47,40	68,2	3,8
1822	12961	367	100	38,8	32,0	34,0	147	38,83	43,2	3,4
1823	13010	465	94	41,7	30,1	29,1	149	35,92	43,2	3,3
1824	12900	402	99	41,0	34,0	34,5	152	45,00	50,5	3,5
1093	14410	474	108	45,1	25,1	31,3	156	46,15	64,6	4,3
1094	13619	416	107	40,5	26,7	27,1	147	40,00	61,4	3,7
1098	14700	456	105	47,5	25,0	27,0	148	36,50	69,5	4,0
1102	13805	455	106	41,5	29,8	32,2	149	38,05	62,4	3,1
1107	15248	470	111	47,5	32,7	36,1	159	36,63	60,9	3,2
596	14078	416	122	43,2	25,2	32,0	172	51,94	60,7	3,3
599	13846	425	98	42,1	36,2	24,0	146	44,80	54,3	3,5
601	15340	482	105	47,6	30,1	31,6	162	47,09	55,8	3,7
604	14673	420	109	42,1	29,4	33,2	147	44,86	58,4	3,6
605	13762	409	100	41,4	28,1	29,5	164	43,81	41,0	3,6
Mean	13781	428	106	42,8	32,5	31,4	154	41,2	56,3	3,5
SD	893	39	12	3,5	6,3	4,2	8	5,1	8,8	0,5
n	20	20	20	20	20	20	20	20	20	20
Min	12184	344	83	34,3	24,5	23,8	140,2	33,8	41,0	2,4
Max	15340	492	133	48,2	47,7	39,1	171,8	51,9	69,5	4,4

† = Seminal vesicles with coagulating gland

Historical control - Collected data - 2018

APPENDIX 14

Historical control

Organ weight
Han:WIST Rat Female: 9 - 10 weeks old

Animal number	Body weight	Organ weight (g)								
		Brain	Liver	Kidneys	Heart	Thymus	Spleen	Uterus	Ovaries	Adrenal glands
1794	164	1,91	5,30	1,31	0,57	0,52	0,33	0,50	-	0,055
1796	172	1,90	5,87	1,42	0,58	0,49	0,44	0,42	-	0,073
1797	166	1,87	5,03	1,38	0,62	0,40	0,50	0,44	-	0,080
1801	163	1,82	5,26	1,20	0,57	0,50	0,32	0,37	-	0,068
1802	170	1,92	6,33	1,41	0,62	0,44	0,44	0,48	-	0,064
1836	160	1,90	4,74	1,35	0,54	0,41	0,33	-	-	0,079
1841	167	1,77	5,21	1,49	0,58	0,49	0,54	-	-	0,065
1843	169	1,98	5,78	1,38	0,54	0,49	0,45	-	-	0,079
1845	172	1,79	5,35	1,51	0,60	0,59	0,40	-	-	0,079
1858	171	1,85	5,30	1,33	0,56	0,35	0,40	-	-	0,057
1120	189	1,84	5,35	1,33	0,58	0,42	0,44	-	-	0,079
1128	195	1,84	6,09	1,48	0,54	0,40	0,49	-	-	0,099
1130	186	1,84	5,33	1,22	0,56	0,28	0,42	-	-	0,077
1131	179	1,92	5,32	1,34	0,57	0,29	0,35	-	-	0,074
1136	181	1,80	5,23	1,24	0,57	0,36	0,44	-	-	0,070
612	181	1,90	5,49	1,44	0,65	0,52	0,43	0,56	0,089	0,068
617	185	1,80	5,68	1,53	0,64	0,54	0,35	0,36	0,108	0,066
621	193	1,93	5,67	1,46	0,55	0,38	0,46	0,48	0,083	0,074
622	183	1,91	6,56	1,56	0,61	0,39	0,38	0,35	0,081	0,069
629	192	1,75	6,61	1,61	0,66	0,50	0,38	0,37	0,113	0,082
Mean	176,9	1,86	5,58	1,40	0,59	0,44	0,41	0,43	0,095	0,073
SD	10,9	0,06	0,50	0,11	0,04	0,08	0,06	0,07	0,015	0,010
n	20	20	20	20	20	20	20	10	5	20
Min	160	1,75	4,74	1,20	0,54	0,28	0,32	0,35	0,081	0,055
Max	195	1,98	6,61	1,61	0,66	0,59	0,54	0,56	0,113	0,099

Remark: '- = No data

Historical control - Collected data - 2018

APPENDIX 14

Historical control

Organ weight
Han:WIST Rat Female: 9 - 10 weeks old

Animal number	Brain	Liver	Kidneys	Heart	Thymus	Spleen	Uterus	Ovaries	Adrenal glands
	Organ weight relative to body weight (%)								
1794	1,16	3,23	0,80	0,35	0,32	0,20	0,30	-	0,0335
1796	1,10	3,41	0,83	0,34	0,28	0,26	0,24	-	0,0424
1797	1,13	3,03	0,83	0,37	0,24	0,30	0,27	-	0,0482
1801	1,12	3,23	0,74	0,35	0,31	0,20	0,23	-	0,0417
1802	1,13	3,72	0,83	0,36	0,26	0,26	0,28	-	0,0376
1836	1,19	2,96	0,84	0,34	0,26	0,21	-	-	0,0494
1841	1,06	3,12	0,89	0,35	0,29	0,32	-	-	0,0389
1843	1,17	3,42	0,82	0,32	0,29	0,27	-	-	0,0467
1845	1,04	3,11	0,88	0,35	0,34	0,23	-	-	0,0459
1858	1,08	3,10	0,78	0,33	0,20	0,23	-	-	0,0333
1120	0,97	2,83	0,70	0,31	0,22	0,23	-	-	0,0418
1128	0,94	3,12	0,76	0,28	0,21	0,25	-	-	0,0508
1130	0,99	2,87	0,66	0,30	0,15	0,23	-	-	0,0414
1131	1,07	2,97	0,75	0,32	0,16	0,20	-	-	0,0413
1136	0,99	2,89	0,69	0,31	0,20	0,24	-	-	0,0387
612	1,05	3,03	0,80	0,36	0,29	0,24	0,31	0,049	0,0376
617	0,97	3,07	0,83	0,35	0,29	0,19	0,19	0,058	0,0357
621	1,00	2,94	0,76	0,28	0,20	0,24	0,25	0,043	0,0383
622	1,04	3,58	0,85	0,33	0,21	0,21	0,19	0,044	0,0377
629	0,91	3,44	0,84	0,34	0,26	0,20	0,19	0,059	0,0427
Mean	1,06	3,15	0,79	0,33	0,25	0,23	0,25	0,051	0,0
SD	0,08	0,25	0,06	0,03	0,05	0,04	0,04	0,008	0,0
n	20	20	20	20	20	20	10	5	20
Min	0,91	2,83	0,66	0,28	0,15	0,19	0,19	0,043	0
Max	1,19	3,72	0,89	0,37	0,34	0,32	0,31	0,059	0

Remark: '- = No data

Historical control - Collected data - 2018

APPENDIX 14

Historical control

Organ weight
Han:WIST Rat Female: 9 - 10 weeks old

Animal number	Organ weight and body weight relative to brain weight (%)								
	Body wight	Liver	Kidneys	Heart	Thymus	Spleen	Uterus	Ovaries	Adrenal glands
1794	8586	277	68,6	29,8	27,2	17,3	26,2	-	2,88
1796	9053	309	74,7	30,5	25,8	23,2	22,1	-	3,84
1797	8877	269	73,8	33,2	21,4	26,7	23,5	-	4,28
1801	8956	289	65,9	31,3	27,5	17,6	20,3	-	3,74
1802	8854	330	73,4	32,3	22,9	22,9	25,0	-	3,33
1836	8421	249	71,1	28,4	21,6	17,4	-	-	4,16
1841	9435	294	84,2	32,8	27,7	30,5	-	-	3,67
1843	8535	292	69,7	27,3	24,7	22,7	-	-	3,99
1845	9609	299	84,4	33,5	33,0	22,3	-	-	4,41
1858	9243	286	71,9	30,3	18,9	21,6	-	-	3,08
1120	10272	291	72,3	31,5	22,8	23,9	-	-	4,29
1128	10598	331	80,4	29,3	21,7	26,6	-	-	5,38
1130	10109	290	66,3	30,4	15,2	22,8	-	-	4,18
1131	9323	277	69,8	29,7	15,1	18,2	-	-	3,85
1136	10056	291	68,9	31,7	20,0	24,4	-	-	3,89
612	9526	289	75,8	34,2	27,4	22,6	29,5	4,68	3,58
617	10278	316	85,0	35,6	30,0	19,4	20,0	6,00	3,67
621	10000	294	75,6	28,5	19,7	23,8	24,9	4,30	3,83
622	9581	343	81,7	31,9	20,4	19,9	18,3	4,24	3,61
629	10971	378	92,0	37,7	28,6	21,7	21,1	6,46	4,69
Mean	9514	300	75,3	31,5	23,6	22,3	23,1	5,1	3,9
SD	720	29	7,1	2,5	4,8	3,4	3,4	1,0	0,6
n	20	20	20	20	20	20	10	5	20
Min	8421	249	65,9	27,3	15,1	17,3	18,3	4,24	2,88
Max	10971	378	92,0	37,7	33,0	30,5	29,5	6,46	5,38

Remark: '- = No data

Historical control - Collected data - 2018

APPENDIX 15

Copy of the Study Plan

TC

TOXI-COOP ZRT.

TOXI-COOP ZRT

Address: Berlini utca 47-49.
H-1045 Budapest
Hungary
Phone: +36-1-920-1228

Study Plan

14-Day Repeated Dose Oral Gavage Toxicity Study of three Fullerenes in Olive Oil - C60/C70, C60, C70 - in Rats

Study no.: **842-400-5742**

Study Director: **Ilona Pasics Szakonyiné**

Date of Study Plan: **January 29, 2021**

(Study Plan including Appendices total pages 29)

Sponsor:

SES RESEARCH Inc.
5999 West 34th Street
Suite 106
Houston,TX 77079 USA

Test Facility

Toxi-Coop Zrt.
Berlini utca 47-49.
H-1045 Budapest
Hungary

APPENDIX 15

Copy of the Study Plan

Table of Contents **Page**

Study plan signatures 4
General statements 5
Experimental schedule 6
Responsibilities 7
1.0 Study objective and introduction 8
2.0 Regulatory guidelines and test methods 8
3.0 Quality assurance 8
3.1 GLP compliance 8
3.2 Amendment procedures 9
3.3 Deviation procedures / interfering factors 9
3.4 Quality assurance evaluations 9
3.5 Archiving 9
4.0 Materials and methods 11
4.1 Test items 11
4.1.1 Characteristics of test items 11
4.1.2 Identification, receipt 11
4.1.3 Formulation 12
4.1.4 Concentration check of the test item 12
4.2 Control item 12
4.3 Characteristics of the other materials 12
4.4 Test system 12
4.4.1 Animals 12
4.4.2 Reason for selection of species 13
4.4.3 Husbandry 13
4.4.4 Identification of animals 14
4.5 Experimental design 14
4.5.1 Route of administration and reason for the selection 14
4.5.2 Randomization 14
4.5.3 Dose levels 14
4.5.4 Justification of dose level selection 15
4.6 Duration of the experimental period 15
5.0 Description of the test procedure 15
5.1 Selection of animals 15
5.2 Administration of test item 15
5.3 Mortality 16
5.4 Clinical observations 16
5.5 Body weight and body weight gain 16
5.6 Food consumption measurement 16
5.7 Clinical pathology 16
5.7.1 Hematology 17
5.7.2 Blood coagulation 17
5.7.3 Clinical chemistry 18

APPENDIX 15

Copy of the Study Plan

5.8 Pathology 19
5.8.1 Necropsy 19
5.8.2 Organ weight 20
5.8.3 Histopathology 21
6.0 Evaluation of experimental data 21
7.0 Animal welfare 22
8.0 Reporting 22
9.0 Distribution of the study plan 23
10.0 References 23
11.0 Abbreviations 24

Appendices:

APPENDIX 1:
Copy of the Certificates of Analysis ..... 4 pages

APPENDIX 2:
Contents of SSNIFF® SM R/M-Z+H Complete diet for rats and mice ..... 1 page

The following print of the study plan is issued:

Paper print: Original is archived at Toxi-Coop Zrt.

APPENDIX 15

Copy of the Study Plan

Study plan signatures

On behalf of Toxi-Coop Zrt.

Ilona Pasics Szakonyiné
Study Director

Date January 29, 2021

Dr. Gábor Hirka
Managing Director

Date Ja 29, 2021

Anett Szegner
Quality Assurance

Date January 29, 2021

Tóth Éva

Éva Tóth
Secretary of IACUC

Date January 29, 2021

On behalf of the Sponsor

John R Endres

Digitally signed by John R Endres
DN: cn=John R Endres, o=AIBMR Life Sciences, ou, email=john@aibmr.com, c=US
Date: 2021.02.01 21:52:20 -08'00'

John R. Endres, ND
Sponsor's Scientific Monitor

February 1, 2021
Date

APPENDIX 15

Copy of the Study Plan

General statements

**Study title:**	**14-Day Repeated Dose Oral Gavage Toxicity Study of three Fullerenes in Olive Oil - C60/C70, C60, C70 - in Rats**
**Study number:**	**842-400-5742**
**Sponsor:**	**SES RESEARCH Inc.**<br>5999 West 34th Street<br>Suite 106<br>Houston,TX 77079 USA
**Sponsor's scientific monitor:**	**John R. Endres, ND**<br>Chief Scientific Officer<br>Natural and Medicinal Products Research<br>AIBMR Life Sciences, Inc.<br>2800 E. Madison St. Suite 202<br>Seattle WA 98112
**Test facility:**	**Toxi-Coop Zrt.**<br>Berlini utca 47-49.<br>H-1045 Budapest<br>Hungary<br><br>Arácsi út 97.<br>H-8230 Balatonfüred,<br>Hungary

APPENDIX 15

Copy of the Study Plan

Study no. 842-400-5742

Experimental schedule

**Proposed date of start of experimental phase:**	February 09, 2021
**Proposed date of end of in-life phase:**	February 23, 2021
**Proposed date of end of experimental phase:**	March 30, 2021

Pre-experimental period

Animal arrival:	February 04, 2021
Veterinary control/acclimatization:	February 04 – 22, 2021
Animal identification:	February 04, 2021
Body weight measurement:	February 05, 22, 2021
Clinical observations:	February 05, 22, 2021
Randomization:	February 08, 2021

Experimental period

Treatment period:	February 09 - 22, 2021
Body weight measurement:	February 09, 12, 16, 19, 22, 2021<br>Before the necropsy: February 23, 2021
Food consumption measurement:	February 09, 16, 22, 2021
Clinical observation:	February 09 - 22, 2021, daily
Blood sampling:	February 23, 2021
Necropsy:	February 23, 2021
Proposed date of Draft Report:	April 12, 2021
Proposed date of Final Report:	Within four weeks after the Sponsor's approval of Draft Report

APPENDIX 15

Copy of the Study Plan

Responsibilities

Test facility management: **Dr. Gábor Hirka**
Phone: +36-1-920-1228
E-mail: gabor.hirka@toxicoop.com

Study director: **Ilona Pasics Szakonyiné**
Phone: +36-30-846-2665
E-mail: ilona.pasics@toxicoop.com

Head of Quality assurance unit: **Ildikó Hermann**
Phone: +36-1-920-1228
E-mail: ildiko.hermann@toxicoop.com

Sponsor's scientific monitor: **John R. Endres, ND**
Phone: (253) 286-2888
john@aibmr.com
http://www.aibmr.com

Histopathology: **Róbert Glávits, D.V.M., Ph.D., D.Sc.**
Histopathologist

Responsible personnel: The names and the responsibilities of other scientists or professionals, and of responsible technical and quality assurance personnel, involved in the study will be part of the raw data and the study report.

APPENDIX 15

Copy of the Study Plan

1.0 Study objective and introduction

The objective of this study is to obtain first information on the toxic potential of three fullerenes – C60/C70, C60 and C70 – i.e. test items Olive Oil infuse with Carbon C60/C70, Olive Oil infuse with Carbon C60 and Olive Oil infuse with Carbon C70 in the groups of male and female rats likely to arise from repeated exposure to the test item over a 14-Day repeat-dose test period.

2.0 Regulatory guidelines and test methods

This study will follow the procedures indicated by the following internationally accepted guidelines and recommendations:

– OECD Guidelines for Testing of Chemicals, Section 4 Health Effects; No. 407, "*Repeated Dose 28-Day Oral Toxicity Study in Rodents*" (adopted 03 October 2008)
– US FDA Toxicological Principles for the Safety Assessment of Food Ingredients, Redbook 2000, IV.C. 3.a. *Short-Term Toxicity Studies with Rodents* (2003).

Three groups of three test item will be examined (instead of three doses of one substance as determined by the test guidelines) at approximately similar concentrations and doses.

3.0 Quality assurance

3.1 GLP compliance

On the request of the Sponsor for authority purposes, the study will be performed in compliance with the principles of Good Laboratory Practice Regulations. The Principles of Good Laboratory Practice as specified by Hungarian and international legislations are followed:

- Hungarian Good Laboratory Practice Regulation: 42/2014 (VIII. 19.) EMMI decree of the Minister of Human Capacities which corresponds to the OECD GLP, ENV/MC/CHEM(98)17)
- OECD Principles of GLP as revised in 1997, published in ENV/MC/CHEM (98)17); OECD, Paris, 1998

Unless otherwise specified, all procedures mentioned in the study plan are the subject of detailed standard operating procedures (SOPs), which are contained in the SOP manuals of the operating departments of Toxi-Coop Zrt.
The Quality Assurance will conduct inspections of the study plan, various phases of the study, certain repetitive operations and the report will be audited according to internal Standard Operating Procedures.

APPENDIX 15

Copy of the Study Plan

3.2 Amendment procedures

This study plan can be amended in consultation with the sponsor (intended changes). Amendments will become effective at the time of the study director's signature. After authorization, the sponsor will return the signed amendment and it will be stored with the original of the study plan. Copies of the amendment will be distributed and added to all copies of the study plan.

3.3 Deviation procedures / interfering factors

The Test Facility undertakes to adopt all reasonable measures to perform the study in accordance with the study plan. Under practical working conditions, however, some minor variations may occur due to circumstances beyond the control of the Test Facility. All such deviations will be documented in the study records with the raw data and reported in the study report the reason for the study plan deviation and its anticipated effect on the outcome of the study. The deviations will be approved by the study director and communicated to the Sponsor.

3.4 Quality assurance evaluations

The study is subjected to quality assurance evaluation. The performance of techniques, as described in the SOPs, is regularly inspected by the quality assurance (QA) of Toxi-Coop Zrt. Study plan, raw data of the study, all relevant phases of the study and the draft and final report are also inspected according to the QA's SOPs. A QA statement, signed by the QA, is included in the final report.

3.5 Archiving

The study documents and samples as listed below will be archived according to the OECD GLP regulations and to the Toxi-Coop Zrt.'s SOP-s in the archives of Toxi-Coop Zrt. Berlini utca 47-49. H-1045 Budapest Hungary:

- all raw data[1] for 15 years,
- the Study plan for 15 years,
- any Study plan Amendment(s) for 15 years,
- one original Final Report for 15 years,
- one sample of the control and test items for 5 years,
- biological samples for 5 or 12 years:
  - -- organs and tissues preserved in 4 % buffered formaldehyde solution for 5 years
  - -- blocks and slides of organs and tissues 12 years
- correspondence for 15 years,

1

A. Information on test item will include but not be limited to the following: Storage; Certificate of analysis of test item; Usage; Disposition;
B. Information on animals should include but not be limited to the following: Receipt, age of animals at starting; Initial health assessment; Dosing; Body weights; Food consumption; Clinical observations; Ophthalmologic evaluations, Hematology, clinical chemistry and coagulation data; Individual necropsy records; Organ weights; Histopathology data;
C. All other records that would demonstrate adherence to the study plan.

APPENDIX 15

Copy of the Study Plan

For the first 5 years archiving is included, thereafter archiving occurs at additional costs of the Sponsor. After this period, the Sponsor will be notified to decide on further archiving to comply with current legal requirements.

After the retention time all the archived materials listed above will be returned to the Sponsor or retained for a further period if agreed by a contract or destroyed on their behalf.
None of the above cited documents or material will be discarded without the explicit written consent of the Sponsor.

At the end of the study, any remaining test item will be returned to the Sponsor or will be discarded, unless otherwise instructed by the Sponsor.

APPENDIX 15

Copy of the Study Plan

4.0 Materials and methods

4.1 Test items

4.1.1 Characteristics of test items

Name of test item 1:	Olive Oil infuse with Carbon C60/C70,
Product Code:	SE20-6070
Lot#:	V0001
Fullerene (C60/C70) content:	850 mg/kg
Relative density:	0.91 g/mL
Appearance:	Brown-reddish liquid
Odor:	Faint oil odor
Manufacturing date:	April 17, 2020
Expiry date:	April 17, 2023
Storage conditions:	At room temperature
Name of test item 2:	Olive Oil infuse with Carbon C60
Product Code:	SE20-142
Lot#:	V01561
Fullerene (C60) content:	830 mg/kg
Relative density:	0.91 g/mL
Appearance:	Reddish-brown- liquid
Odor:	Faint oil odor
Manufacturing date:	April 17, 2020
Expiry date:	April 17, 2023
Storage conditions:	At room temperature
Name of test item 3:	Olive Oil infuse with Carbon C70
Product Code:	SE20-0070
Lot#:	V0011
Fullerene (C70) content:	830 mg/kg
Relative density:	0.91 g/mL
Appearance:	Dark red liquid
Odor:	Faint oil odor
Manufacturing date:	April 17, 2020
Expiry date:	April 17, 2023
Storage conditions:	At room temperature

(Information based on the Certificate of Analysis, Material Safety Data Sheet and correspondence with Sponsor's Scientific Monitor.)

4.1.2 Identification, receipt

The test items of a suitable chemical purity, certificate of analysis, safety data sheet and specification of the product were supplied by the Sponsor. All precautions required in the handling and disposal of the test items were outlined by the Sponsor. Identification of test items was made in Toxi-Coop Zrt. on the basis of the information included in the Certificates of Analysis (see appendix 1) and MSDS.

APPENDIX 15

Copy of the Study Plan

4.1.3 Formulation

Formulation of the test items is not necessary. The three fullerenes (Olive Oil infuse with Carbon C60/C70, Olive Oil infuse with Carbon C60 and Olive Oil infuse with Carbon C70) as well as the control item (EVO Olive Oil) were provided by the Sponsor in "ready to use" form.

4.1.4 Concentration check of the test item

Analytical control of dosing formulations (control of concentration) will be not performed within the scope of this study. The sponsor provided the analytical certificates for control and test items prepared in "ready to use" form.

4.2 Control item

Name:	EVO Olive Oil
Product Code:	SE20-EVOO
Lot#:	V100
Appearance:	Golden-greenish liquid
Odor:	Faint oil odor
Manufacturing date:	April 17, 2020
Expiry date:	April 17, 2023
Storage conditions:	At room temperature

4.3 Characteristics of the other materials

Name:	Isofluran CP®
Batch number:	G150G19A
Expiry date:	June 2024
Supplier:	Medicus Partner Kft.<br>Biatorbágy, Tormásrét u. 12.<br>H-2051 Hungary
Storage conditions:	Below30 °C
Purpose of use:	Anesthesia during the blood collection and euthanasia

Specification of new batches of anesthetics will be given in the Study report and raw data if needed.

4.4 Test system

4.4.1 Animals

Species / Strain:	Han:WIST rat of Wistar origin
Source:	Toxi-Coop Zrt. 1103 Budapest, Cserkesz u. 90.
Hygienic level:	SPF (Specific pathogen-free) at arrival and kept in good conventional environment during the study.
Age at the commencement of the treatment:	Young adult rats, less than 9 weeks old
Body weights at arrival:	The weight variation will not exceed ± 20 per cent of the mean weight

APPENDIX 15

Copy of the Study Plan

Number and sex of animals:	40 rats (20 male and 20 female - nulliparous and non-pregnant animals)
Number of groups:	4 (3 test items treated + 1 control group)
Number of animals/groups:	10 (5 male; 5 female)
Animal health:	Only healthy animals will be used for the study. Healthy status will be certified by the breeder.

4.4.2 Reason for selection of species

The rat is commonly used species for toxicological studies in accordance with international recommendations. The Wistar rat is the system of choice because it has been the preferred and most commonly used species for toxicity tests is a well-known laboratory model with sufficient historical data.

4.4.3 Husbandry

4.4.3.1 Housing conditions

Animal room no.:	18/1 and 18/2
Housing:	Individual caging
Cage type:	Type III polypropylene/polycarbonate (conform to the size recommendations in the latest *Guide for the Care and Use of Laboratory Animals (*Natl. Res. Council, 2011*)*[2].
Bedding:	Certified laboratory wood bedding (SAFE 3/ 4-S-FASERN produced by J. Rettenmaier & Söhne GmbH+Co.KG; D-73494 Rosenberg Holzmühle 1 Germany). The cages and bedding will be changed once or twice a week.
Illumination:	Artificial light, from 6 a.m. to 6 p.m.<br>(except days of ophthalmology examinations)
Temperature:	22 ± 3 °C
Relative humidity:	30 - 70 %
Ventilation:	Above 10 air-exchanges/ hour by a central air-condition system.
Acclimatization time:	5 days

Environmental conditions are maintained by an air-condition system. Temperature and relative humidity will be verified and recorded daily during the study.

4.4.3.2 Food and water supply

Animals will receive ssniff® SM R/M-Z+H complete diet for rats and mice produced by ssniff Spezialdiäten GmbH, D-59494 Soest Germany and tap water, as for human consumption, *ad libitum* except overnight food deprivation before blood sampling.

The food is considered not to contain any contaminants that could reasonably be expected to affect the purpose or integrity of the study. The supplier will provide an analytical certificate of the standard diet for the batch used. Contents of standard diet for rats and mice guaranteed by the supplier are presented in Appendix 2.

[2] National Research Council Guide for the Care and Use of Laboratory Animals, Inst. Lab. Anim. Res., Comm. Life Sci., Natl. Acad. Press, 8th Edition, Washington, D.C., 2011.

APPENDIX 15

Copy of the Study Plan

Animals will receive tap water from watering bottles. Water quality control analysis and microbiological assessment are performed once in every six months by Government Office of Capital Budapest Department of Public Health and Medical Officer Service (Váci út 172-174. Budapest, H-1138 Hungary). The quality control results are available at Toxi-Coop Zrt.'s archives.

4.4.4 Identification of animals

Individual identification will be performed by numbers on the tail of the animals written with a permanent marker. The numbers will be given on the basis of the laboratory master file of Toxi-Coop Zrt. and will be re-marked as necessary to ensure correct identification.
The cages will be marked by identity cards, with information at least about the study number, control or test item name, group number, sex, cage number and individual animal numbers, mode of administration, start of the treatment, date of the necropsy. Cages will be arranged in such a way that possible effects due to cage placement are minimized.

4.5 Experimental design

4.5.1 Route of administration and reason for the selection

The test item will be administered orally via gavage. The route of application is selected in compliance with international guidelines (See references in section 2). The oral route is the anticipated route of human exposure to the test item.

4.5.2 Randomization

Animals will be randomly assigned to test groups. All animals will be sorted according to body weight by computer and grouped according to weight ranges.
There will be an equal number of animals from each weight group in each of the experimental groups during the randomization.
The grouping will be controlled by SPSS/PC+ computer program according to the actual body weight verifying the homogeneity and deviations among the groups.

4.5.3 Dose levels

A control and three test items treated groups will be involved in the study. Table below contains the group number, name of control and test items, doses, dosing volume and number of animals.

Table 1: Experimental design

Group number	Name and concentration † of control or test items	Dose†† (mg/kg bw/day)	Dose volume (mL/kg bw)	Number of animals	
				Male	Female
Group 1	EVO Olive Oil 0 mg/mL	0	5	5	5

APPENDIX 15

Copy of the Study Plan

Table 1: Experimental design

Group number	Name and concentration † of control or test items	Dose†† (mg/kg bw/day)	Dose volume (mL/kg bw)	Number of animals	
				Male	Female
Group 2	Olive Oil Infuse with Carbon C60/C70 0.77 mg/ml	4 (3.85)	5	5	5
Group 3	Olive Oil Infuse with Carbon C60 0.76 mg/ml	4 (3.8)	5	5	5
Group 4	Olive Oil Infuse with Carbon C70 0.76 mg/ml	4 (3.8)	5	5	5

† Concentrations by C60/70, C60 or C70;

††Doses calculated by C60/70, C60 or C70 concentrations, respectively.

Animals in Group 1 will only receive the control item, EVO Olive Oil.

4.5.4 Justification of dose level selection

The Sponsor, in consultation with the Study Director, selected the dose levels to target approximate exposures of 0 and 4 mg/kg bw/day of control and test items C60/70, C60 or C70, respectively.

4.6 Duration of the experimental period

The experimental period involves 5 days of acclimatization, 14 days treatment and observation periods and necropsy on the following day (Day 14).
The day of first treatment is considered as Day 0 of examination.

5.0 Description of the test procedure

5.1 Selection of animals

Forty (40) healthy rats (twenty males and twenty females) will be used on test. Animals will be selected for this study on the basis of adequate body weight, freedom from clinical signs of disease or injury, and a body weight within ± 20% of the mean within a sex.
Selected rats will be distributed by randomization according to stratification by body weight so that there will be no statistically significant difference among group body weight means within a sex.

5.2 Administration of test item

The control and test items are to be administered to the appropriate animals by once daily oral gavage approximately the same time each day morning within a 2-3 hours period from Day 0 up to Day 13 and Day 14. The dose volume for each animal will be based on the most recent body weight measurement.
Animals will not be treated on the day of necropsy.

APPENDIX 15

Copy of the Study Plan

5.3 Mortality

Inspection for signs of morbidity and mortality will be made twice daily at the beginning and at the end of the working day. Moribund animals or animals obviously in pain or showing signs of severe and enduring distress will be euthanized. These animals will be processed in the same way as the animals of the terminal necropsy. Mortality checks will be recorded.

5.4 Clinical observations

General clinical observations will be made cage-side once a day, after treatment at approximately the same time.
On the day prior to the first treatment with the test item, and approximately once weekly thereafter, a detailed observation will be conducted while handling the animal on days that the animals are weighed and food consumption measurements are taken. Potential signs noted include but are not limited to: changes in skin, fur, eyes, and mucous membranes, occurrence of secretions and excretions and autonomic activity (e.g., lacrimation, piloerection, pupil size, and unusual respiratory pattern).
Likewise, changes in gait, posture and response to handling as well as the presence of clonic or tonic movements, stereotype activities (e.g., excessive grooming, repetitive circling), or bizarre behavior (e.g., self-mutilation, walking backwards) will be recorded. All observations will be recorded.
The Study Director will be advised when an animal is found in a moribund condition and may authorize euthanasia and necropsy as necessary to avoid the loss of quality data. All such authorizations will be recorded in the raw data.

5.5 Body weight and body weight gain

Individual body weights will be recorded two times during acclimatization. Animals involved in the study will be weighed with an accuracy of 1 g on Day 0 (prior to study start) and twice weekly thereafter (i.e. on Days 0, 3, 7, 10 and 13).
The animals will also be weighed prior to sacrifice (on Day 14) in order to calculate organ to body weight ratios. Decedents will be also weighed. Individual body weight changes will be calculated according to the days of measurements and for the study overall.

5.6 Food consumption measurement

Food consumption will be determined with the measurement of given and non-consumed diet with a precision of 1 g once weekly to coincide with body weight measurements (Days 0, 7 and 13). Food consumption will be evaluated and reported by weekly interval for each group. Feed efficiency will be calculated and reported. Feed efficiency will be calculated on the basis of the weekly body weight gain and food consumption.

5.7 Clinical pathology

Clinical pathology examinations including hematology, blood coagulation and clinical chemistry will be conducted at termination of the treatment (i.e. one day after the last treatment: on Day 14).

APPENDIX 15

Copy of the Study Plan

Animals will be food deprived overnight (for approximately 16 hours) prior to blood collection. Blood samples will be harvested from the retro orbital venous plexus under Isofluran CP® anesthesia.
Three samples will be taken from each animal: one for hematology, one for determination of blood clotting times and the third one to obtain serum samples for clinical chemistry.

5.7.1 Hematology

Blood samples for hematology measurements will be collected in tubes containing K3EDTA (spray-dried; MiniCollect® 0.5 mL, manufactured by Greiner Bio-One International AG, Kremsmünster, Austria; or equivalent) and tubes should be filled up to the final volume marked on the tubes. Blood will be analyzed immediately after sampling (may be stored at 2-8 °C until analysis not longer than for 24 hours).
The parameters listed in Table 3 will be measured in all animals by Siemens ADVIA120:

Table 2: Hematology parameters

PARAMETERS	UNIT	METHODS
**WBC**<br>White Blood Cell (leukocyte) count	10^9/L (G/L)	Flow cytometry method
**RBC**<br>Red Blood Cell (erythrocyte) count	10^{12}/L (T/L)	Flow cytometry method
**HGB**<br>Hemoglobin concentration	g/L	Cyanide-colorimetric hemoglobin method
**HCT**<br>Hematocrit (relative volume of erythrocytes)	L/L	Computed by equipment
**MCV**<br>Mean Corpuscular (erythrocyte) Volume	fL	Flow cytometry method
**MCH**<br>Mean Corpuscular (erythrocyte) Hemoglobin	pg	Computed by equipment
**MCHC**<br>Mean Corpuscular (erythrocyte) Hemoglobin Concentration	g/L	Computed by equipment
**PLT**<br>Platelet (thrombocyte) count	10^9/L (G/L)	Flow cytometry method
**RET**<br>Reticulocytes,	%	Flow cytometry method
**Differential white blood cell count†**	%	Peroxidase and basophil/ lobularity method

† Notes:
NEU: Neutrophil (%) **LYM:** Lymphocyte (%) **EOS:** Eosinophil; (%)
MONO: Monocyte (%) **BASO:** Basophil (%)

5.7.2 Blood coagulation

Blood samples for determination of blood clotting times (APTT and PT) will be collected in tubes containing 9NC Coagulation sodium citrate 3.2 % (MiniCollect® 1 mL; manufactured by Greiner Bio-One International AG, Kremsmünster, Austria; or equivalent). Tubes should be filled up to the final volume marked on the tubes.

Blood will be centrifuged at 2500 rpm for 15 minutes within 20 – 30 minutes after the sampling. Supernatant plasma samples will be measured immediately after harvesting (may be stored at 2-8 °C and measured within 3 hours or should be frozen between (-20 °C) and (-80 °C) then should be measured within 30 days).The following parameters will be measured in all animals by Sysmex CA-1500:

Table 3: Blood coagulation parameters

PARAMETERS	UNIT	METHODS
**APTT**<br>Activated partial Thromboplastin Time	sec	Optical
**PT**<br>Prothrombin Time	sec	Optical

5.7.3 Clinical chemistry

Blood samples collected for clinical chemistry measurements will be drawn in tubes Vacuette 2.5 mL Z Serum Sep C/A (no anticoagulant; manufactured by Greiner Bio-One International AG, Kremsmünster, Austria; or equivalent).
At least 1.0 mL blood should be collected if possible, into clinical chemistry tubes. Samples will be stored in a dark place at room temperature for 30-40 minutes and then centrifuged at 4000 rpm for 15 minutes. Serum samples will be stored at 2-8 °C (or frozen between minus 20 and minus 30 °C) and measured within 7 days.

The following parameters will be measured in all animals by Cobas C311:

Table 4: Clinical chemistry parameters

PARAMETERS	UNIT	METHODS
**ALT**<br>Alanine Aminotransferase activity	U/L	IFCC recommended (with P-5'-P), 3-reagent system
**AST**<br>Aspartate Aminotransferase activity	U/L	IFCC recommended (with P-5'-P), 3-reagent system
**GGT**<br>Gamma Glutamyltransferase activity	U/L	IFCC recommended enzymatic method
**ALP**<br>Alkaline Phosphatase activity	U/L	IFCC (AMP) *2-Amino-2-metyl-1-propanol*
**TBIL**<br>Total Bilirubin concentration	µmol/L	Colorimetric diazo method (NBD: *p-nitrobenzene-diazonium)*
**CREA**<br>Creatinine concentration	µmol/L	Enzymatic method
**UREA**<br>Urea concentration	mmol/L	Urease-GLDH method
**GLUC**<br>Glucose concentration	mmol/L	Hexokinase method
**CHOL**<br>Cholesterol concentration	mmol/L	Enzymatic CHOD-POD method

APPENDIX 15

Copy of the Study Plan

Table 4: Clinical chemistry parameters

PARAMETERS	UNIT	METHODS
**Pi**<br>Inorganic phosphate concentration	mmol/L	Ammonium-molybdate
**Ca^{++}**<br>Calcium concentration	mmol/L	(NM-BAPTA)-EDTA method
**Na^{+}**<br>Sodium concentration	mmol/L	Potentiometric test (Direct ISE)
**K^{+}**<br>Potassium concentration	mmol/L	Potentiometric test (Direct ISE)
**Cl^{-}**<br>Chloride concentration	mmol/L	Potentiometric test (Direct ISE)
**ALB**<br>Albumin concentration	g/L	Colorimetric - BCG (Bromocresol green) - metod
**TPROT**<br>Total Protein concentration	g/L	Colorimetric – Biuret - method
**A/G**<br>Albumin/globulin ratio	–	Calculated value

5.8 Pathology

5.8.1 Necropsy

Gross pathology will be performed on every experimental animal irrespective of the date of its death: animals died during the study or are removed from the study for animal welfare reasons and at termination of the treatment.

Scheduled sacrifice:
Animals will be anesthetized with Isofluran CP® and will be exsanguinated from the abdominal aorta after verification of deep narcosis.
The external appearance (surface of the body, all orifices) will be examined, cranium, thoracic and abdominal cavities will be opened and the appearance of the tissues and organs will be observed macroscopically. All observations will be recorded with details of the location, color, shape and size.

The following organs/ tissues will be removed and preserved in 4 % buffered formaldehyde solution, except testes and epididymides, which will be preserved in modified Davidson solution and then stored in 4 % buffered formaldehyde solution for future histopathological examination:

Table 5: List of organs to be preserved

Adrenal glands
Aorta
Bone with joint and marrow (femur)
Brain (representative regions: cerebrum, cerebellum and pons and medulla oblongata)

APPENDIX 15

Copy of the Study Plan

Table 5: List of organs to be preserved

Esophagus
Eyes (lachrymal gland with Harderian glands)
Gonads (testes with epididymides, ovaries, uterus with vagina)
Gross lesions
Heart
Kidneys
Large intestines (caecum, colon, rectum, including Peyer's patches),
Liver
Lungs (with main stem bronchi; inflation with fixative and then immersion;)
Lymph nodes (submandibular, mesenteric)
Mammary gland
Muscle (quadriceps)
Nasal turbinates
Pancreas
Pituitary
Prostate
Salivary glands (submandibular)
Sciatic nerve
Seminal vesicle with coagulating gland
Skin
Small intestines (representative regions: duodenum, ileum, jejunum)
Spinal cord (at three levels: cervical, mid-thoracic and lumbar)
Spleen
Sternum
Stomach
Thymus
Thyroid + parathyroid
Trachea
Urinary bladder

* Thyroid and parathyroid will be preserved together with larynx but larynx will not be processed histologically.

Unscheduled sacrifice:

Any rat that dies or is sacrificed because of a moribund condition will be examined for the cause of death or moribund condition on the day the observation is made. Rats will be evaluated for gross lesions. All organs/ tissues will be removed and preserved as specified above.

Organs and tissues will be excised, weighed (except for animals found dead) and preserved as described for those animals sacrificed by design.

5.8.2 Organ weight

The following organs will be weighed and recorded. Paired organs will be weighed together.

With precision of 0.01g:	Liver, kidneys, testes, epididymides, prostate + seminal vesicles with coagulating glands as a whole, uterus and fallopian tubes, thymus, spleen, brain and heart.
With precision of 0.001g:	Adrenal glands, ovaries

APPENDIX 15

Copy of the Study Plan

Paired organs will be weighed together. If a visible difference exists between the sizes of the two organs, paired organs are measured individually.

5.8.3 Histopathology

Histopathological examination will be performed on the preserved organs and tissues of the animals from both the control and test items treated groups (Groups 1, 2, 3 and 4) as well as from any animal that dies during the course of the study. In addition, gross lesions of potential toxicological significance noted in any test groups at the time of terminal sacrifice will also be examined.
Examination of parathyroids will be performed if feasible (i.e. section plane of thyroids crosses parathyroids).
The fixed tissues will be trimmed, processed (dehydrated), embedded in paraffin, sectioned with a microtome (at a thickness of 2-4 µm, placed on glass microscope slides, stained with hematoxylin and eosin and examined by light microscopy.

6.0 Evaluation of experimental data

Statistical analysis will be done with SPSS PC+ software for the following data:

- Body weight
- Food consumption
- Feed efficiency
- Hematology
- Blood coagulation
- Clinical chemistry
- Organ weight

The heterogeneity of variance between groups will be checked by Bartlett's homogeneity of variance test. Where no significant heterogeneity is detected, a one-way analysis of variance will be carried out. If the obtained result is positive, Duncan's Multiple Range test will be used to assess the significance of inter-group differences.
Where significant heterogeneity is found, the normal distribution of data will be examined by Kolmogorov-Smirnov test. In case of a none-normal distribution, the non-parametric method of Kruskal-Wallis One-Way analysis of variance will be used. If there is a positive result, the inter-group comparisons are performed using the Mann-Whitney U-test.

The use of the word “significant” or “significantly” indicates a statistically significant difference between the control and the test item treated groups. Significance will be judged at a probability value of $p < 0.05$ and < 0.01. Male and female rats will be evaluated separately.

Frequency of toxic response, pathological and histopathological findings by sex and dose will be calculated.

APPENDIX 15

Copy of the Study Plan

7.0 Animal welfare

Institutional Animal Care and Use Committee (IACUC) of Toxi-Coop Zrt. permit the conduct of the study by signature on the Study Plan. (SOP: ALT 023 - Instructions for animal protection)
The study will be conducted according to the National Research Council Guide for the Care and Use of Laboratory Animals and in compliance with the principles of the Hungarian Act 2011 CLVIII (modification of Hungarian Act 1998 XXVIII) and Government Decree 40/2013 regulating animal protection.

8.0 Reporting

The results of the study will be reported in a detailed Final Report in English. The Final Report will include but will not be limited to:
General information and statements:
- Name and address of the Sponsor, the test facility and the study schedule;
- The names of the study director and other scientists and supervisory personnel involved in the study;
- The statement of GLP compliance, signed by the study director;
- The signature and statement of the management;
- The Quality Assurance statement, signature QA;
- A list of all deviations to the Study plan and a statement on their possible impact on quality and integrity of the study results;
- Details of archiving (the storage location, list of archived data and samples, time for archiving);

Test item:
- Characterization of test item components: The identification of the test item components, either by name or code number. The concentration, purity, stability, composition and other appropriate characteristics of the test item components, if the Sponsor provides data;
- A copy of the analytical certificate of the test item;

Test animals:
- A description of the animals: species/strain, source, health status (microbiological, if known), number, age, sex, identification, weight at commencement of the study, group size;
- Details of housing conditions (room number, caging, bedding material, environmental conditions, etc.);
- Details of food and water quality (including diet type/ source, water source);

Test Conditions:
- Rationale dose level selection;
- Details of the test item formulation;
- Details of the administration of the test item (way of dosing, time of dosing, treatment procedures);

APPENDIX 15

Copy of the Study Plan

Results:
- All investigated parameters will be presented in individual and summary tables;
- Toxic response data by sex and dose level, including signs of toxicity;
- Nature, severity and duration of clinical observations (whether reversible or not);
- Body weight/body weight changes;
- Food consumption and feed efficiency;
- Hematological tests;
- Clinical biochemistry tests;
- Necropsy findings;
- Body weight at necropsy and organ weight data (absolute and organ to body weight and organ to brain weight ratios);
- Histopathology findings;
- Statistical treatment of results, where appropriate

Discussion and interpretation of the results

Conclusion

9.0 Distribution of the study plan

Study Director: Original
Sponsor: PDF
Internal units†: PDF
† = Quality assurance, formulation, clinical pathology and histopathology laboratory, statistical unit.

10.0 References

1) OECD Principles of Good Laboratory Practice, adopted by Council on 26th November 1997; Environment Directorate, Organisation for Economic Cooperation and Development, Paris 1998. (OECD Principles of GLP as revised in 1997, published in ENV/MC/CHEM (98)17); OECD, Paris, 1998)
2) Hungarian Good Laboratory Practice Regulation: 42/2014 (VIII. 19.) EMMI decree of the Minister of Human Capacities which corresponds to the OECD GLP, ENV/MC/CHEM(98)17).
3) OECD Guidelines for Testing of Chemicals, Section 4 Health Effects; No. 407, "*Repeated Dose 28-Day Oral Toxicity Study in Rodents*" (adopted 03 October 2008)
4) US FDA Toxicological Principles for the Safety Assessment of Food Ingredients, Redbook 2000, IV.C. 3. a. *Short-Term Toxicity Studies with Rodents* (2003).
5) National Research Council. Guide for the Care and Use of Laboratory Animals. Inst. Lab. Anim. Res., Comm. Life Sci., Natl. Acad. Press, 8th Edition, Washington, D.C., 2011.

APPENDIX 15

Copy of the Study Plan

11.0 Abbreviations

Ad libitum	at will
APP/ App.	Appendix
Bw or bw	Body weight
°C	Degrees centigrade
cm	Centimeter
DAkkS:	Deutsche Akkreditierungsstelle (D-PL-14082-01-00)
DVM	Doctor of Veterinary Medicine
EC	European Community
EMMI	Emberi Erőforrások Minisztériuma (Ministry of Human Resources)
GLP	Good Laboratory Practice
GmbH	Gesellschaft mit beschränkter Haftung (Limited Liability Company)
h	Hour(s)
kg, g, mg	Kilogram, gram, milligram
L, mL	Liter, milliliter
Ltd.	Limited Liability Corporation/Company
MSDS	Material Safety Data Sheet
min.	Minute(s)
OECD	Organisation for Economic Co-operation and Development
PDF	Portable Document Format
PhD	Doctor of Philosophy
Ref.	Reference
QA	Quality Assurance
SD	Standard deviation
SOP	Standard Operating Procedures
SPF	Specific Pathogen Free
u	Utca (street in Hungarian)
Zrt.	Zártkörűen működő Részvénytársaság (Private Limited Company)
%	Percentage
±	Plus/minus

APPENDIX 15

Copy of the Study Plan

APPENDIX 1

Copy of the Certificates of Analysis

EVO Olive Oil

SES research

5999 WEST 34TH STREET, SUITE 106, HOUSTON TEXAS 77092 713-686-9662

FAX 713-686-9635

Certificate of Analysis # C685

Product Name	EVO Olive Oil			
Product Code:	SE20-EVOO			
Lot#	V100			
Production Date	04/17/2020			
Expiration Date	04/17/2023			
Items		Method	Specification	Results
Appearance		Observation	Golden greenish liquid	PASS
Odor		Smell	Faint oil odor	PASS
Fullerene content (mg/kg)		HPLC	0	0
Heavy Metals (mg/kg or ppm)	Hg	Methods EAM 4.7	<0.010	PASS
	Pb	Methods EAM 4.7	<0.010	PASS
	As	Methods EAM 4.7	0.012	PASS
	Cd	Methods EAM 4.7	<0.010	PASS

Microbiological Specification	Quantity	Units	Method
Total Plate Count	<3000	CFU / g	USP <61>
Yeast & Mold	<300	CFU / g	AOAC
Coliforms	<3	MPN / g	FDA (BAM) Ch.4
E. Coli	Negative		USP<62>
Pseudomonas Aeruginosa	Negative		USP<62>
Salmonella	Negative		USP<62>
Staphylococcus aureus	Negative		USP<62>

SES Research
Owner

Mr. Robert Wong — date: 10/20/2020

Page 1 of 1

APPENDIX 15

Copy of the Study Plan

APPENDIX 1

Copy of the Certificates of Analysis
Olive Oil infuse with Carbon C60/C70

SES research
5999 WEST 34TH STREET, SUITE 106. HOUSTON TEXAS 77092 713-686-9662
FAX 713-686-9635

Certificate of Analysis # C686

Product Name	Olive Oil infuse with Carbon C60/C70		
Product Code:	SE20-6070		
Lot#	V0001		
Production Date	04/17/2020		
Expiration Date	04/17/2023		
**Items**	**Method**	**Specification**	**Results**
Appearance	Observation	Brown Reddish liquid	PASS
Odor	Smell	Faint oil odor	PASS
Fullerene content (mg/kg)	HPLC	800-870	850
Heavy Metals (mg/kg or ppm) Hg	Method EAM 4.7	<0.010	PASS
Heavy Metals (mg/kg or ppm) Pb	Methods EAM 4.7	<0.010	PASS
Heavy Metals (mg/kg or ppm) As	Methods EAM 4.7	0.012	PASS
Heavy Metals (mg/kg or ppm) Cd	Methods EAM 4.7	<0.010	PASS

Microbiological Specification	Quantity	Units	Method
Total Plate Count	<3000	CFU / g	USP <61>
Yeast & Mold	<300	CFU / g	AOAC
Coliforms	<3	MPN / g	FDA (BAM) Ch.4
E. Coli	Negative		USP<62>
Pseudomonas Aeruginosa	Negative		USP<62>
Salmonella	Negative		USP<62>
Staphylococcus aureus	Negative		USP<62>

SES Research
Owner

Mr. Robert Wong 10/20/2020
date

APPENDIX 15

Copy of the Study Plan

APPENDIX 1

Copy of the Certificates of Analysis
Olive Oil infuse with Carbon C60

SES research
5999 WEST 34TH STREET, SUITE 106. HOUSTON TEXAS 77092
713-686-9662
FAX 713-686-9635

Certificate of Analysis # C688

Product Name		Olive Oil infuse with Carbon 60		
Product Code:		SE20-142		
Lot#		V01561		
Production Date		04/17/2020		
Expiration Date		04/17/2023		
Items		Method	Specification	Results
Appearance		Observation	Reddish Brown liquid	PASS
Odor		Smell	Faint oil odor	PASS
Fullerene content (mg/kg)		HPLC	800-870	830
Heavy Metals (mg/kg or ppm)	Hg	Methods EAM 4.7	<0. 010	PASS
	Pb	Methods EAM 4.7	<0. 010	PASS
	As	Methods EAM 4.7	0. 012	PASS
	Cd	Methods EAM 4.7	<0. 010	PASS

Microbiological Specification	Quantity	Units	Method
Total Plate Count	<3000	CFU / g	USP <61>
Yeast & Mold	<300	CFU / g	AOAC
Coliforms	<3	MPN / g	FDA (BAM) Ch.4
E. Coli	Negative		USP<62>
Pseudomonas Aeruginosa	Negative		USP<62>
Salmonella	Negative		USP<62>
Staphylococcus aureus	Negative		USP<62>

SES Research
Owner

Mr. Robert Wong 10/20/2020
date

APPENDIX 15

Copy of the Study Plan

APPENDIX 1

Copy of the Certificates of Analysis

Olive Oil infuse with Carbon C70

SES research
5999 WEST 34TH STREET, SUITE 106. HOUSTON TEXAS 77092
713-686-9662
FAX 713-686-9635

Certificate of Analysis # C687

Product Name	Olive Oil infuse with Carbon 70		
Product Code:	SE20-0070		
Lot#	V0011		
Production Date	04/17/2020		
Expiration Date	04/17/2023		

Items		Method	Specification	Results
Appearance		Observation	Dark Red liquid	PASS
Odor		Smell	Faint oil odor	PASS
Fullerene content (mg/kg)		HPLC	800-870	830
Heavy Metals (mg/kg or ppm)	Hg	Methods RAM 4.7	<0. 010	PASS
	Pb	Methods RAM 4.7	<0. 010	PASS
	As	Methods RAM 4.7	0. 012	PASS
	Cd	Methods RAM 4.7	<0. 010	PASS

Microbiological Specification	Quantity	Units	Method
Total Plate Count	<3000	CFU / g	USP <61>
Yeast & Mold	<300	CFU / g	AOAC
Coliforms	<3	MPN / g	FDA (BAM) Ch.4
E. Coli	Negative		USP<62>
Pseudomonas Aeruginosa	Negative		USP<62>
Salmonella	Negative		USP<62>
Staphylococcus aureus	Negative		USP<62>

SES Research
Owner

Mr. Robert Wong 10/20/2020
date

Page 1 of 1

APPENDIX 15

Copy of the Study Plan

APPENDIX 2

Contents of
SSNIFF® SM R/M-Z+H Complete diet for rats and mice

S8106-S011 15 mm Pellets

Producer: ssniff Spezialdiäten GmbH

Crude nutrients [%]

Dry matter	88.4
Crude protein	19.0
Crude fat	3.5
Crude fibre	3.6
Crude ash	6.5
N free extracts	55.9
Starch	39.5
Sugar	4.8

Minerals [%]

Calcium	1.00
Phosphorus	0.70
Sodium	0.20
Magnesium	0.22
Potassium	0.78

Amino acids [%]

Lysine	1.12
Methionine	0.56
Met+Cys	0.89
Threonine	0.63
Thryptophan	0.23
Arginine	1.08
Histidine	0.43
Valine	0.84
Isoleucine	0.72
Leucine	1.25
Phenylalanine	0.82
Phe+Tyr	1.36
Glycine	0.77
Glutamic acid	3.99
Aspartatic acid	1.45
Proline	1.28
Alanine	0.75
Serine	0.86

Vitamins (per kg)

Vitamin A	25 000	IU
Vitamin D3	1 000	IU
Vitamin E	135	mg
Vitamin K (as menadione)	20	mg
Thiamine (B1)	86	mg
Riboflavin (B2)	32	mg
Pyridoxine (B6)	31	mg
Cobalamin (B12)	150	µg
Nicotinic acid	170	mg
Pantothenic acid	62	mg
Folic acid	10	mg
Biotin	730	µg
Choline-Chloride	2 910	mg
Inositol	100	mg

Trace elements (per kg)

Iron	166	mg
Manganese	71	mg
Zinc	94	mg
Copper	15	mg
Iodine	2.2	mg
Selenium	0.3	mg
Cobalt	2.1	mg

Energy

Metabolizable Energy:	13.4	MJ/kg

These data are standard and guaranteed values provided by the supplier.

APPENDIX 16

Copy of the Good Laboratory Practice (GLP) Certificate of the Test Facility

OGYÉI
Országos Gyógyszerészeti
és Élelmezés-egészségügyi Intézet

1051 Budapest, Zrínyi u. 3.
Levélcím: 1372 Postafiók 450.
Tel.: (1) 8869-300, Fax: (1) 8869-460
E-mail: ogyei@ogyei.gov.hu, Web: www.ogyei.gov.hu

Ref. no: OGYÉI/8623-5/2019
Admin.: dr. Szaller Zoltán
Date: 22 May, 2019

GOOD LABORATORY PRACTICE (GLP) CERTIFICATE

It is hereby certified that the test facility

TOXI-COOP Toxicolgical Research Center Zrt.

H-1103 Budapest, Cserkesz u. 90.,
H-1045 Budapest, Berlini u. 47-49.,
H-8230 Balatonfüred, Arácsi u. 97-99.,
H-8230 Balatonfüred, Vasút u. 3.,
H-8230 Balatonfüred, Galamb u. 12/A ,
H-8230 Balatonfüred, Ady E. u. 12,
8354 Karmacs, hrsz 4150/2

is able to carry out

physico-chemical testing, toxicity studies, mutagenicity studies, environmental toxicity studies on aquatic and terrestrial organisms, studies on behaviour in water, soil and air; bio-accumulation studies, analytical and clinical chemistry, safety pharmacology testing, metabolism and toxico/pharmacokinetics testing, testing of toxicological properties of operative procedures and equipment, reproduction toxicological studies, tolerance studies, inhalation toxicology and in vitro studies

in compliance with the Principles of GLP (Good Laboratory Practice) and also complies with the corresponding OECD/European Community requirements.

Date of the inspection: **18-26. February 2019.**

dr. Mittner András
Digitálisan aláírta: dr. Mittner András
Dátum: 2019.05.23 09:01:24 +02'00'

Dr. András Mittner
Head of Inspectorate
